# The maize (*Zea mays* ssp. *mays* var. B73) genome encodes 33 members of the purple acid phosphatase family

**DOI:** 10.3389/fpls.2015.00341

**Published:** 2015-05-19

**Authors:** Eliécer González-Muñoz, Aida-Odette Avendaño-Vázquez, Ricardo A. Chávez Montes, Stefan de Folter, Liliana Andrés-Hernández, Cei Abreu-Goodger, Ruairidh J. H. Sawers

**Affiliations:** Laboratorio Nacional de Genómica para la Biodiversidad, Centro de Investigación y de Estudios Avanzados del Instituto Politécnico NacionalIrapuato, Mexico

**Keywords:** purple acid phosphatase, maize, *Zea mays*, phosphorus

## Abstract

Purple acid phosphatases (PAPs) play an important role in plant phosphorus nutrition, both by liberating phosphorus from organic sources in the soil and by modulating distribution within the plant throughout growth and development. Furthermore, members of the PAP protein family have been implicated in a broader role in plant mineral homeostasis, stress responses and development. We have identified 33 candidate PAP encoding gene models in the maize (*Zea mays* ssp. *mays* var. B73) reference genome. The maize *Pap* family includes a clear single-copy ortholog of the *Arabidopsis* gene *AtPAP26*, shown previously to encode both major intracellular and secreted acid phosphatase activities. Certain groups of PAPs present in *Arabidopsis*, however, are absent in maize, while the maize family contains a number of expansions, including a distinct radiation not present in *Arabidopsis*. Analysis of RNA-sequencing based transcriptome data revealed accumulation of maize *Pap* transcripts in multiple plant tissues at multiple stages of development, and increased accumulation of specific transcripts under low phosphorus availability. These data suggest the maize PAP family as a whole to have broad significance throughout the plant life cycle, while highlighting potential functional specialization of individual family members.

## Introduction

Phosphorus (P) is an essential nutrient for plant growth: it is required for the majority of metabolic processes, including photosynthesis and respiration, and is a key structural component of macromolecules such as phospholipids and nucleic acids. In agricultural systems P is typically limiting and, as a consequence, large quantities of P additions are required to maintain productivity. It is now widely accepted that global P reserves are limited and need to be better managed to achieve maximum benefit (Schroder et al., 2011). Furthermore, such resources may not be readily available in marginal or remote areas. Among the multiple factors contributing to efficient agricultural P use, the capacity of the crops themselves is of clear importance (Veneklaas et al., 2012).

Plant P Efficiency (PE) can be partitioned between the efficiency of uptake (P Acquisition Efficiency; PAE), and the efficiency of internal use (P Use Efficiency; PUE; reviewed in Veneklaas et al., 2012). One of the greatest obstacles to PE is the relative immobility of P. The ability of a plant to take up and internally distribute P rests largely on the transport of inorganic orthophosphate (Pi) by the members of PHT1 family (Bucher, 2007). Much of the P, however, both in the soil and in the plant is in the form of immobile organic P esters. In the soil, organic P is present not only as the result of direct entry of organic sources into the system, but also as the result of soil microorganisms acting on Pi additions: organic P can account for up to 80% of soil total P (Ticconi and Abel, 2004). In the plant, incorporation of P into organic forms is a natural consequence of P use. Therefore, more active or appropriate mobilization of organic P sources, both external and internal, has the capacity to enhance both PAE and PUE. As such, liberation of Pi from organic sources represents both an intriguing point of convergence in the mechanistic basis of overall PE and an area of considerable research interest.

Acid phosphatases (AP; E.C.3.1.3.2) represent a major route for the liberation of Pi from organic P sources. APs can hydrolyse a broad range of Pi-monoesters, with optimal activity under acidic conditions. While plant genomes encode a variety of putative APs, the largest group, the purple AP (PAP) family, plays the most significant role in P foraging and recycling (Tran et al., 2010a). PAPs belong to the metallophosphoesterase superfamily, and are characterized by 5 conserved blocks of residues (**D**xG-G**D**XX**Y**-G**N**H(D/E)-VXX**H**-G**H**X**H**; metal binding residues are shown in bold; the spacing between blocks is variable) that bind metal ions, in plants typically Fe(III)–Zn(II) or Fe(III)–Mg(II), to form the enzymatic site. While mammalian genomes encode only a small number of *PAP* genes, the family is greatly expanded in plants: in *Arabidopsis* (*A. thaliana)* there are 29 members of the PAP family (Li et al., 2002); in rice (*Oryza sativa*) there are 26 (Zhang et al., 2011); in soybean (*Glycine max*) there are 35 (Li et al., 2012). Plant PAPs have been assigned to three major groups on the basis of predicted protein sequences (Li et al., 2002): groups I and II consist of high molecular weight oligomeric PAPs; group III consists of mammalian-like low molecular weight monomeric PAPs (Li et al., 2002; Tran et al., 2010a).

In *Arabidopsis*, biochemical and genetic evidence have identified the gene *AtPAP26* to encode both the major intracellular (vacuolar) and extracellular (secreted) PAP activities, consistent with a dual role in P foraging and P recycling that has been confirmed by analysis of *Atpap26* mutants (Veljanovski et al., 2006; Hurley et al., 2010; Tran et al., 2010b; Robinson et al., 2012a,b; Wang et al., 2014). Vacuolar and secreted forms of AtPAP26 are processed identically with respect to cleavage of an N-terminal targeting sequence. There are differences, however, in glycosylation, and these have been suggested to play a key role in determining protein localization (Tran et al., 2010b). Despite a predominant role in the P-starvation response, accumulation of *AtPAP26* transcripts is not regulated by P availability, although transcript abundance has been observed to increase in senescing leaves (Gepstein et al., 2003; Robinson et al., 2012a). Identification of *PAP26* orthologs from other plant species and phylogenetic analysis have revealed the PAP26 clade to diverge from other homologous sequences. In addition, with the exception of two sequences identified from each of *Populus* (Tran et al., 2010a) and soybean (Li et al., 2012), PAP26 is encoded by a single-copy gene in all species so far analyzed (Tran et al., 2010a,b; Zhang et al., 2011). When secreted AP activities have been purified from soybean and tomato (*Lycopersicon esculentum*) cell cultures, *PAP26* products have been identified, suggesting that PAP26 plays a key role across plant taxa (Lebansky et al., 1992; Bozzo et al., 2002). In white lupin (*Lupinus albus*), intracellular and extracellular AP activities are largely divided between the products of *SAP1* and *SAP2*, two genes that, if not direct orthologs to *AtPAP26*, are closely related (Tang et al., 2013). Notwithstanding the predominant role of PAP26, further PAPs make important contributions to P foraging and remobilization under Pi deprivation response—most notably AtPAP12, that contributes to secreted AP activity (Tran et al., 2010b), and AtPAP10, that has been localized to the root surface (Wang et al., 2011). AtPAP26, AtPAP12 and AtPAP10 together form a clade distinct from the other 29 *Arabidopsis* PAPs, designated group Ia-2 (Li et al., 2002).

Maize is a crop plant of global importance, and one for which much of the planting area is P limited (Calderon-Vazquez et al., 2011). The capacity of maize to secrete AP from roots has been well characterized at a physiological level. Furthermore, natural variation has been observed in AP production and, in a number of cases, such variation has been mapped genetically (Chen et al., 2008). Intracellular AP activities have been less well characterized, although isozyme variation in seedlings has been used successfully for analyses of genetic diversity (Efron, 1971). The genes encoding maize AP activities, however, remain largely uncharacterized. On the basis of studies in other plants, we hypothesize that the genes encoding the major activities related to the maize Pi deprivation response will belong to the PAP group Ia-2. In this study, we report the identification of 33 candidate PAP-encoding genes from the maize reference genome, and a phylogenetic analysis defining the maize PAP group Ia-2. In addition to identification of clear maize orthologs of *AtPAP26* and *AtPAP10*, we report two further sequences that, while members of group Ia-2, define a radiation that is not present in *Arabidopsis*.

## Materials and methods

### Bioinformatic identification of maize *Pap* genes

The protein sequences (TAIR10 representative gene model) of the 29 *Arabidopsis PAP* genes identified by Li et al. (2002) were retrieved and aligned using MUSCLE version 3.8.31 (Edgar, 2004). The resulting block-alignment file was converted to Stockholm 1.0 format, and used as input to hmmbuild (HMMER suite version 3.1b1; Finn et al., 2011). The resulting hmm file was used to search (hmmsearch) maize primary transcript predicted protein sequences (version 284_6a; Schnable et al., 2009), obtained from Phytozome 10 (Goodstein et al., 2012). A total of 39 protein sequences was selected based on an inclusion threshold of *E*-value <0.01 and aligned with MUSCLE, identifying the five conserved blocks of amino acids reported previously to be present in PAP proteins (GDXG/GDXXY/GNH(D/E)/VXXH/GHXH; Li et al., 2002; Supplementary Table S1). In the case of GRMZM5G831009 the protein sequence predicted from the primary transcript contained only two of the five conserved blocks. A secondary splicing model (GRMZM5G831009_T02), however, was predicted to encode all five conserved blocks and, consequently, was used for subsequent analysis. A second round of searching using hmmsearch identified an additional seven candidate proteins. The resulting set of 46 candidate maize PAPs was inspected and 13 sequences removed on the basis of the absence of conserved residues (GRMZM2G076062, GRMZM2G373887, AC209374.4, GRMZM2G019019, GRMZM2G076989, GRMZM2G143984, GRMZM2G150236, GRMZM2G306712, GRMZM2G342815, GRMZM2G375011, GRMZM2G405770) or a non-canonical order of the conserved blocks (GRMZM2G046436 and GRMZM2G404941). The final list of 33 maize PAP-coding genes is presented in Supplementary Table S2.

### Phylogenetic analyses

**Figure 1:**
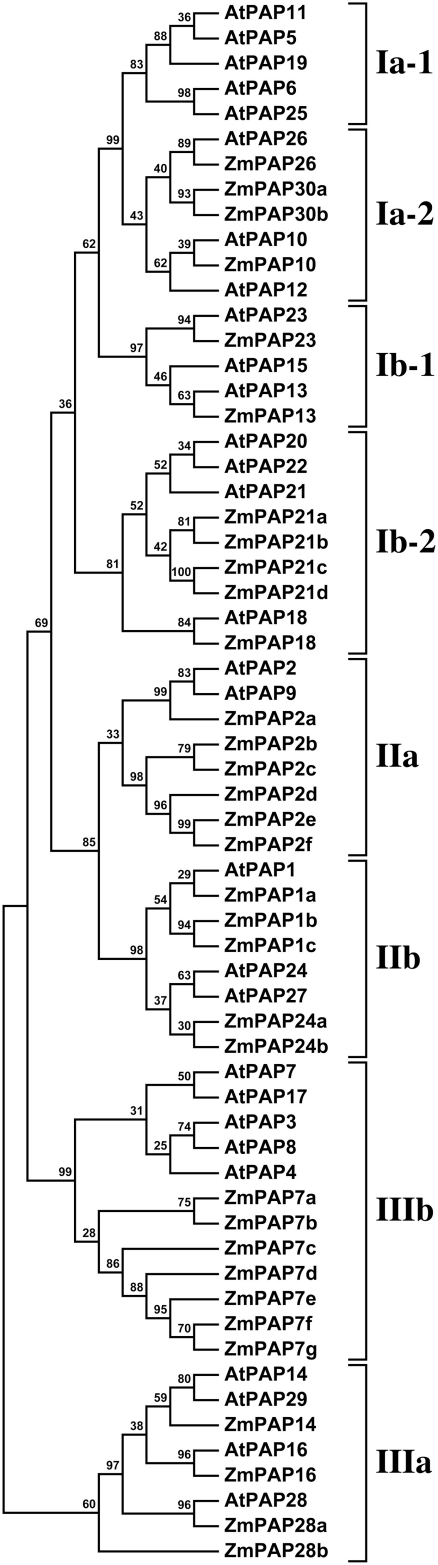
**Phylogenetic analysis of maize and *Arabidopsis* PAP sequences**. A maximum likelihood phylogeny was generated from 33 predicted maize PAP protein sequences and 29 PAP protein sequences from *Arabidopsis*. The eight previously defined subgroups (Li et al., 2002) are labeled to the right of the tree: Groups I and II (I, II) consist of high molecular weight oligomeric PAPs; group III (III) consists of mammalian-like low molecular weight monomeric PAPs. Bootstrap support values are shown as percentage at the nodes.

**Figure 2:**
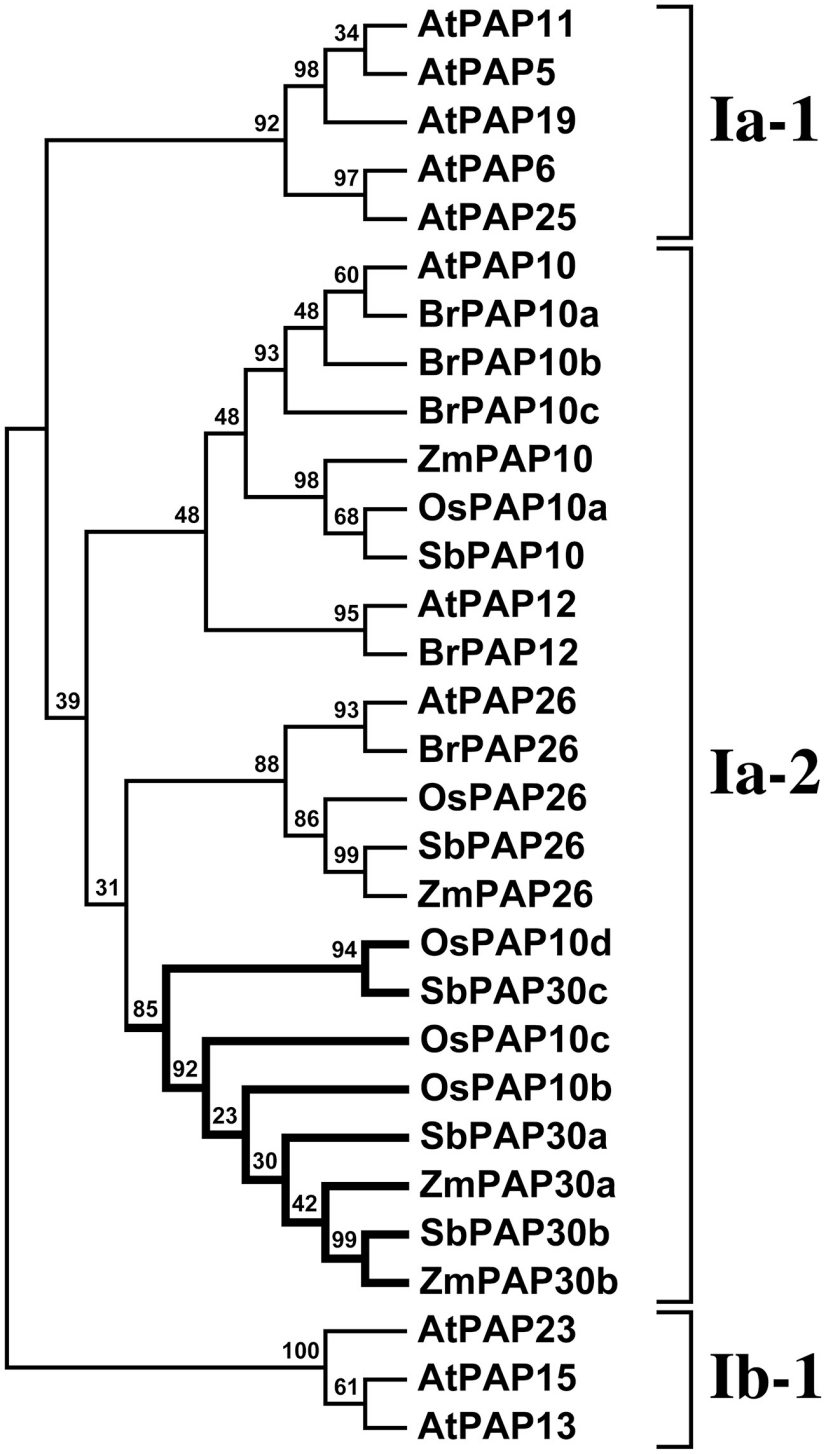
**Phylogenetic analysis of subgroup Ia-2 PAP sequences**. A maximum likelihood phylogeny was generated from subgroup Ia-2 PAP protein sequences from *Arabidopsis thaliana*, canola, sorghum, rice and maize. *Arabidopsis* subgroups Ia-1 and Ib-1 are also shown. A clade that in this analysis is constituted only by grass sequences is highlighted in bold. Bootstrap support values are shown as percentage at the nodes.

Phylogenetic analyses were performed using the MEGA software, version 6.06 (Tamura et al., 2013). Protein sequences were aligned using MUSCLE with manual adjustment, and the resulting alignment used to construct a maximum likelihood tree, bootstrapped using 1000 replicates. Figure 1 was constructed using predicted protein sequences corresponding to 33 candidate maize *PAPs*, together with the previously reported 29 *Arabidopsis* proteins (Li et al., 2002). Figure 2 was constructed using subgroup Ia proteins as defined in *Arabidopsis* (Li et al., 2002) and rice (Zhang et al., 2011) and selected sequences from maize, Canola (*Brassica rapa*) and sorghum (*Sorghum bicolor*). Canola and sorghum sequences were obtained from Phytozome 10 (Goodstein et al., 2012) by BLASTP using ZmPAP26 as the search term. The retrieved sequences were, from canola: Brara.C04146.1 (annotated here BrPAP10a), Brara.I00994.1 (BrPAP10b), Brara.C04145.1 (BrPAP10c), Brara.K01047.1 (BrPAP12), Brara.H00671.1 (BrPAP26); from sorghum: Sobic.003G314400.1 (annotated here SbPAP10; encoded by gene Sb03g036210), Sobic.008G113000.1 (SbPAP30a; Sb08g016690), Sobic.009G250300.1 (SbPAP30b; Sb09g030100), Sobic.008G189500.1 (SbPAP30c; Sb08g023000), Sobic.010G205200.1 (SbPAP26; Sb10g025250). *Arabidopsis* subgroup Ib protein sequences AtPAP13, AtPAP15, and AtPAP23 were also included in the analysis. Sequences were aligned using MUSCLE and a phylogenetic tree was constructed using the MEGA6.06 software with 1000 bootstrap replicates.

### Identification of P1BS PHR1 binding sites

The MYB transcription factor PHOSPHATE STARVATION RESPONSE 1 (PHR1) has been characterized previously to bind the promoter region of its targets at the imperfect palindromic sequence GNATATNC, defined as the PHR1 Binding Sequence (P1BS; Rubio et al., 2001; Zhou et al., 2008). To identify P1BS sequences in the upstream region of *ZmPap* genes, the PLACE Web signal scan (http://www.dna.affrc.go.jp/PLACE/signalscan.html; (Prestridge, 1991; Higo et al., 1999) was used to query the region 2500 bp upstream of the annotated transcriptional start site, using default parameters.

### Prediction of sub-cellular localization

Signal peptide analysis was conducted using the SignalP 4.1 server (http://www.cbs.dtu.dk/services/SignalP/; Petersen et al., 2011) using default settings. Subcelluar localization of ZmPAP proteins was predicted using the TargetP1.1 server (http://www.cbs.dtu.dk/services/TargetP/; Emanuelsson et al., 2007) using default parameters.

### Tissue- and stage-specific *ZmPAP* expression

Normalized expression data for 33 maize PAP-coding genes were retrieved from qTeller (www.qteller.com) using two sources profiling seedling tissues (Wang et al., 2009; Li et al., 2010) and one source profiling reproductive tissues (Davidson et al., 2011). Data was selected for seedling root, seedling shoot, developing leaf, mature leaf (vegetative tissues); and tassel, developing ear, seed 5 days-after-pollination (DAP), seed 10 DAP, embryo 25 DAP and endosperm 25 DAP (reproductive tissues). Data was presented either as absolute (log10 counts) or relative expression across tissue types on a per gene basis. Relative values were calculated as standardized Z-scores obtained by dividing deviations from the gene mean by the gene standard deviation.

### RNA-sequence analysis of maize plants grown under low-phosphate availability

Maize (*Zea mays* ssp. *mays* var. B73) was grown for 21 days post-emergence in ~15 cm diameter × 50 cm height PVC tubes, filled with 9 L of inert sand substrate. From day10, plants were fertilized with Hoagland solution (Hoagland and Broyer, 1936) containing the standard (complete) 1000 μM (+P) or a modified 10 μM (–P) concentration of KH_2_PO_4_. Plants were watered every other day to maintain field-capacity. Watering alternated fertilizer solution and deionized water. The concentration of potassium was maintained constant by addition of KCl as required. The distal 10 cm of the second and third fully expanded leaves and the complete root system were collected for analysis. Two biological replicates were collected for each experimental condition and tissue type. Tissue was ground in liquid nitrogen and total RNA extracted using the PureLink RNA Mini Kit (Ambion by Life Technologies, CA, USA). The integrity, quality and quantity of the RNA was assayed by agarose-electrophoresis, NanoDrop 1000 spectrophotometric analysis (Thermo Fisher Scientific Inc., DE, USA) and by using the Agilent Bioanalyzer system (Agilent Technologies Inc., CA, USA). A sample of 4 μg of total RNA was fragmented, converted to cDNA and enriched by PCR according to the Illumina TruSeq RNA SamplePrep v2 protocol (Illumina, Inc., CA, USA). Libraries were assayed by agarose-electrophoresis and quantified using the microplate based PicoGreen dsDNA quantification assay (Molecular Probes Inc., OR, USA) and Agilent Bioanalyzer system. Illumina barcodes were added and used to pool 10 samples per lane. Libraries were quantified by qPCR, using the Library Quantification kit (Kapa Biosystems, Inc., MA, USA) with the CFX Connect Real-Time PCR Detection System (Bio-rad, CA, USA) and sequenced with the Illumina HiSeq 2500 system using a TruSeq SBS sequencing kit v3 (Illumina, Inc., CA, USA). Reads were mapped to the maize transcript file (version 284_6a, downloaded from Phytozome 10) using bowtie2 v2.2.4 (Langmead and Salzberg, 2012) with the options—very-sensitive and -a (Supplementary Table S3). Bowtie2 Sequence Alignment/Map output files were used as input to eXpress (Roberts and Pachter, 2013) to obtain read counts for all identified *Pap* transcripts. Differential accumulation of transcripts was estimated using EdgeR (Robinson et al., 2010), applying a Bonferroni correction to obtain adjusted *p*-values at the level of the *Pap* family. Transcripts with an adjusted *p*-value of < 0.05 were considered to accumulate differentially between +P and –P conditions (Supplementary Table S4). Raw and processed data are available at NCBI's Gene Expression Omnibus database (http://www.ncbi.nlm.nih.gov/geo/) under accession GSE63779.

## Results

### The maize genome encodes 33 putative PAPs

To identify maize PAP encoding genes, we performed a hidden Markov model search (see Materials and Methods) for maize gene-models whose putative protein products exhibit a high degree of similarity to reported *Arabidopsis* PAPs (Li et al., 2002). A total of 33 putative PAP-encoding genes were identified from the maize B73 set. Predicted maize PAP protein sequences were aligned with the *Arabidopsis* PAPs, and the alignment used to construct a phylogenetic tree that served as a guide to naming the maize PAPs (Figure 1). On the basis of this alignment, maize PAPs were confirmed to encode five previously reported conserved metal binding blocks (Li et al., 2002), with the exception of six proteins (ZmPAP2e, ZmPAP2f, ZmPAP7b, ZmPAP7f, ZmPAP7g, ZmPAP21d) that lacked one or more block (Supplementary Tables S1, S2).

### The maize genome encodes PAPs of groups I, II, and III

The members of the *Arabidopsis* PAP family constitute three major groups (Li et al., 2002): groups I and II comprised of high molecular weight PAPs, and group III comprised of low molecular weight mammalian-like PAPs. All three groups were recovered in our phylogenetic analysis of *Arabidopsis* and maize PAPs (Figure 1). Eleven of a total of 33 maize PAPs were assigned to group I, fewer than the 15 members of the *Arabidopsis* group. Notably, subgroup Ia-1, consisting of AtPAP5, AtPAP6, AtPAP11, AtPAP19 and AtPAP25, was found to be absent from the maize genome, as it is also from rice (Zhang et al., 2011). In contrast, we observed a slight expansion of group II in maize: eleven maize PAPs were assigned to group II, in comparison to 5 *Arabidopsis* PAPs. The expansion of the maize group II resulted from the presence of 5 maize PAPs (ZmPAP2b–2f) that form a sister clade to the *Arabidopsis* group IIa proteins AtPAP2 and AtPAP9, and of a triplication of ZmPAP1 (ZmPAP1a–1c) in group IIb. Group III was comparable in size in maize and *Arabidopsis* (11 and 9 members, respectively). Group IIIa (AtPAP14, AtPAP16, AtPAP28, AtPAP29) was present in maize, although it has been reported to be absent in rice (Zhang et al., 2011). Group IIIb was expanded in maize, notably by the presence of 7 proteins (ZmPAP7a–7g) forming a sister clade to the *Arabidopsis* group IIIb; this maize group likely drives the displacement of group IIIa to a position outside of the other proteins in our analysis.

### The maize PAP subgroup Ia-2 contains four members

It has been shown previously in *Arabidopsis* that the genes encoding the major P responsive AP activities form a phylogenetically distinct clade, subgroup Ia-2, within the PAP group I (Li et al., 2002; Tran et al., 2010b; Wang et al., 2011). The maize PAP subgroup Ia-2 contains 4 proteins (Figure 1). A single maize gene-model (AC211394.4_FG004; Table 1) was identified as a potential ortholog of the key PAP-encoding gene *AtPAP26*, and designated *ZmPap26*. Potential orthology among *Arabidopsis*, rice and maize *PAP26* sequences was supported further by reciprocal BLAST searches (data not shown). A single maize gene-model (GRMZM2G093101; Table 1) was identified also as a potential ortholog of *AtPAP10*, and designated *ZmPap10*. The putative protein ZmPAP10 is most similar to the putative rice protein OsPAP10a. Again, reciprocal BLAST searches (data not shown) supported orthology among *PAP10* sequences. Collectively, our analyses suggest *PAP10* and *PAP26*, genes reported previously to play key roles in the *Arabidopsis* P deprivation response, to be conserved single-copy genes in maize. In contrast, orthology relationships with respect to *AtPAP12* were less clear. A previous analysis in rice identified a triplet of paralagous genes lying adjacent to one another on chromosome 12 that grouped with *OsPAP26, OsPAP10a*, and the *Arabidopsis* subgroup Ia-2 sequences in phylogenetic analysis (Zhang et al., 2011). These three gene-models were designated *OsPAP10b*, *OsPAP10c* and *OsPAP10d*, although their placement with respect to *AtPAP10* and *AtPAP12* was ambiguous. We identified two closely related maize gene-models (GRMZM2G073860 and GRMZM2G077466; Table 1) that show no well-defined greater affinity to either *AtPAP10* or *AtPAP12*, although clearly similar to both, and forming part of groupIa-2. We have designated these sequences *ZmPap30a* and *ZmPap30b*.

**Table 1:** **PHR1 binding sites (P1BS) upstream of *ZmPAP* genes, and predicted subcellular localization for the corresponding ZmPAP proteins**.

**Name**	**P1BS number**	**P1BS location^a^ (bp)**	**Subcellular localization^b^**
*ZmPap26*	1	−2352	S
*ZmPap30a*	1	−256	S
*ZmPap30b*	0		S
*ZmPap10*	2	−232, −2463	S
*ZmPap23*	1	−199	M
*ZmPap13*	1	−412	S
*ZmPap21a*	0		S
*ZmPap21b*	0		O
*ZmPap21c*	4	−326, −1676, −1773, −1819	S
*ZmPap21d*	2	−1462, −2357	S
*ZmPap18*	0		S
*ZmPap2a*	2	−138, −2268	S
*ZmPap2b*	1	−618	M
*ZmPap2c*	2	−2038, −2140	O
*ZmPap2d*	2	−498, −791	C
*ZmPap2e*	1	−824	C
*ZmPap2f*	0		O
*ZmPap1a*	2	−391, −451	M
*ZmPap1b*	1	−2147	M
*ZmPap1c*	1	−191	M
*ZmPap24a*	0		S
*ZmPap24b*	0		S
*ZmPap7a*	0		S
*ZmPap7b*	0		O
*ZmPap7c*	0		S
*ZmPap7d*	4	−436, −666, −708, −1811	S
*ZmPap7e*	1	−446	O
*ZmPap7f*	0		O
*ZmPap7g*	1	−1368	M
*ZmPap14*	0		M
*ZmPap16*	1	−266	M
*ZmPap28a*	0		S
*ZmPap28b*	0		S

a *Location of predicted P1BS relative to predicted translational start*.

b *S, secretory; M, mitochondrial; C, chloroplast; O, other subcellular location*.

### The PAP subgroup Ia-2 in grasses contains lineage specific members

To investigate further orthology relationships among *Arabidopsis* and maize subgroup Ia-2 sequences, we performed a second phylogenetic analysis incorporating subgroup Ia maize PAPs, previously reported subgroup Ia sequences from *Arabidopsi*s (Li et al., 2002) and rice (Zhang et al., 2011), and additional closely related gene-model translations identified from the genome sequences of canola (*Brassica rapa*) and sorghum (*Soghum bicolor*) (Figure 2). This analysis supported the division of subgroups Ia-1 and Ia-2 as previously described (Li et al., 2002). The *Arabidopsis* sequences AtPAP10, AtPAP12, and AtPAP26 defined three distinct groups within subgroup Ia-2. The PAP26 group branched from all other subgroup Ia-2 members and contained sequences unique to each species in the analysis. The PAP10 group contained unique sequences for all species with the exception of canola for which three sequences were identified. The PAP12 group was represented only by *Arabidopsis* and canola sequences. A fourth group within the subgroup Ia-2 was composed specifically of grass sequences in our analysis. We retain the previous annotation of rice PAPs in this grass specific group but extend the PAP30 nomenclature assigned above to maize sequences to sorghum, highlighting the position of this group relative the AtPAP10-AtPAP12 clade.

### Maize PAP transcripts exhibit tissue- and stage- specific patterns of accumulation

**Figure 3:**
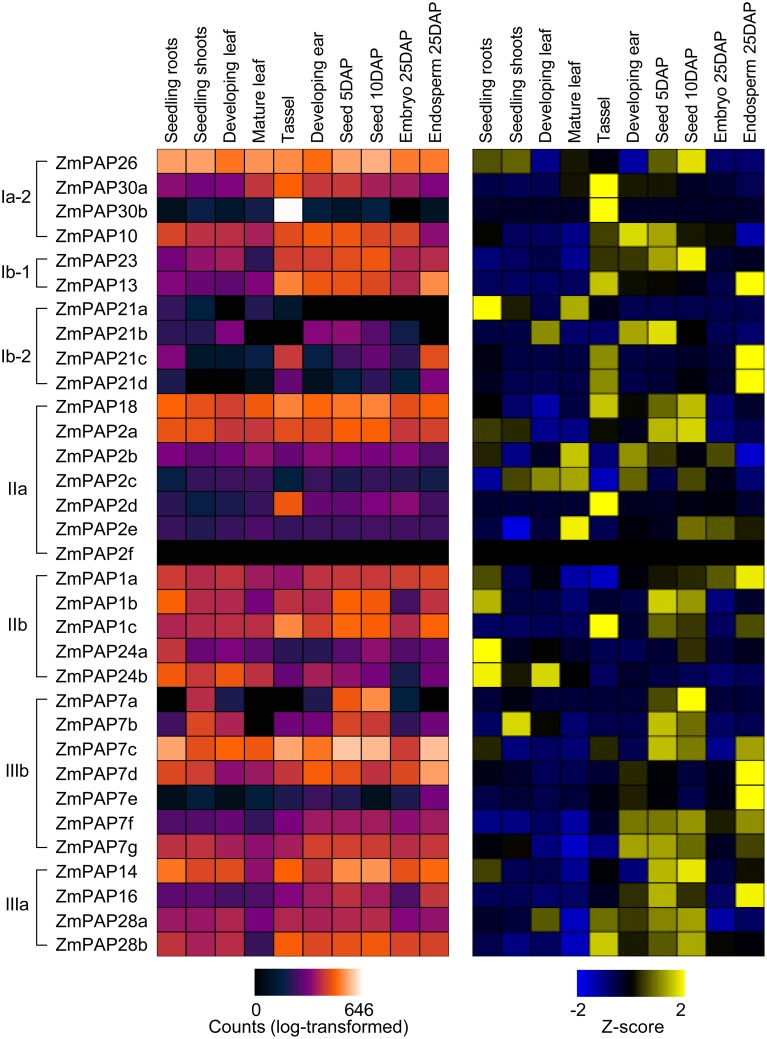
**Tissue- and stage-specific expression of *ZmPap* genes**. Heatmap representation of absolute (left; purple-red scale) and relative (right; blue-yellow scale) accumulation of maize *Pap* transcripts in samples from vegetative (seedling root, seedling shoot, developing leaf and mature leaf) and reproductive (tassel, developing ear, seed 5 days-after-pollination (DAP), seed 10 DAP, embryo 25 DAP, and endosperm 25 DAP) tissues. Primary RNA-sequence transcriptome data from Wang et al. (2009); Li et al. (2010), and Davidson et al. (2011), obtained from qTeller (www.qteller.com). Absolute accumulation is shown as log10-transformed expression values (counts; log-transformed). Relative transcript accumulation across treatments is shown as standardized deviations (Z-score) from the transcript mean expression value. Gene order follows that of the phylogenetic tree in Figure 1.

To begin to investigate functional divergence among maize PAPs, we examined existing transcriptome datasets for evidence of differential accumulation of PAP encoding transcripts. We examined data from two sources profiling seedling tissues (Wang et al., 2009; Li et al., 2010) and one source profiling reproductive tissues (Davidson et al., 2011). Collectively, maize *Pap* transcripts were found to accumulate in all tissues and development stages examined, while the pattern of accumulation varied greatly among individual transcripts, indicating both the importance on the *Pap* gene family throughout the plant lifecycle and a high degree of specialization among family members (Figure 3). Interestingly, we observed no clear relationship between our phylogenetic analysis and expression data, suggesting that functional divergence at the level of transcript accumulation has occurred rapidly during evolution of the maize *Pap* family. In seedlings, there was a broad trend toward greater accumulation of *Pap* transcripts in roots than in the shoot. In later vegetative-stages, however, accumulation increased in the aerial portion of the plant, with a number of *Pap* transcripts accumulating to their highest levels in mature leaves. In reproductive tissues, a number of *Pap* transcripts showed strong accumulation in the tassel. In addition, there was a trend towards increasing *Pap* transcript accumulation in the seed in the days following pollination, and a greater accumulation of *Pap* transcripts in the endosperm than in the embryo. In absolute terms, the accumulation of *Pap* transcripts is dominated by *ZmPap7a, ZmPap26*, *ZmPap18*, and *ZmPap2a* (in descending order of mean counts). Levels of *ZmPap26* transcript accumulation differed little among samples, consistent with previous reports of the importance of post-transcriptional regulation of PAP26 (Tran et al., 2010b). In contrast, transcripts of *ZmPAP7a*, *ZmPAP18*, and *ZmPAP2a* were not only generally abundant, but regulated with tissue type and stage (Figure 3).

### Accumulation of a subset of maize PAP transcripts responds to phosphate availability

**Figure 4:**
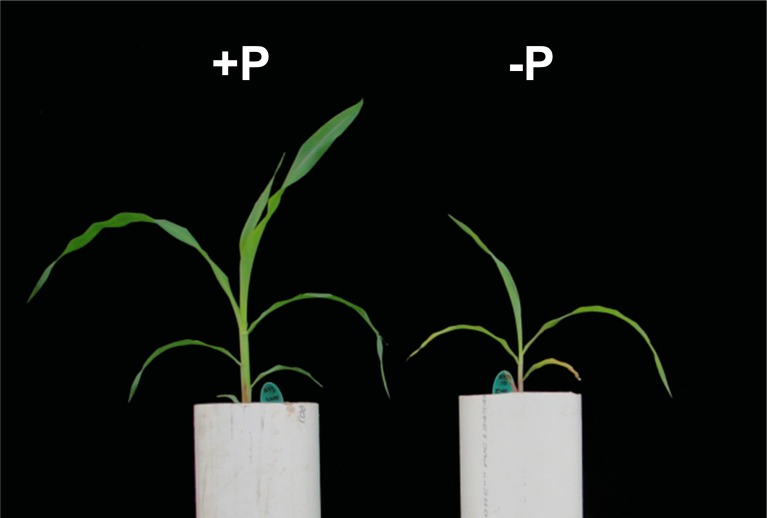
**Plant growth under P sufficient and P limiting conditions**. Maize plants (var. B73) were grown for 21-days post emergence in 9L of inert sand substrate. From day 10, plants were fertilized with Hoagland solution (Hoagland and Broyer, 1936) containing either 1000 μM (left; +P) or 10 μM (right; –P) Pi.

**Figure 5:**
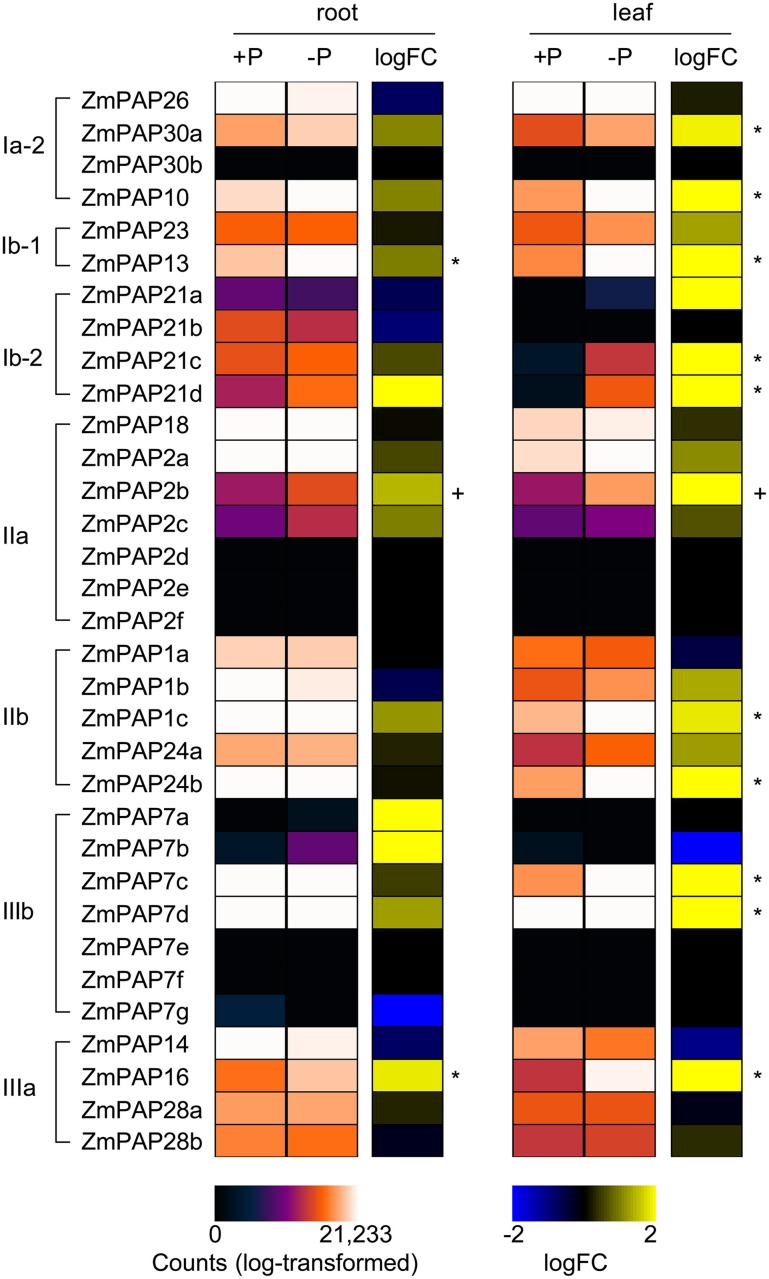
***ZmPap* transcript accumulation in response to low phosphate availability**. Heatmap representation of absolute (purple-red scale) and relative (logFC; blue-yellow scale) accumulation of maize *Pap* transcripts under P sufficiency (+P) and P limitation (–P) (see Materials and Methods). Transcript accumulation was assayed in roots (root) and leaves (leaf) of 21-day-old plants by analysis of RNA-sequence transcriptomes. Absolute accumulation is shown as log10-transformed expression values (counts; log-transformed). Accumulation in –P relative to +P is shown as log2 fold-change (logFC). Asterisks indicate transcripts accumulating differentially between P regimes in a given tissue type (adjusted *p*-value < 0.05). In the case of *ZmPap2b*, data corresponding to the differentially-accumulating secondary transcript GRMZM2G138756_T01, which encodes a protein lacking four of the five conserved blocks, is shown in place of the primary transcript, which did not respond to P availability (indicated “+”). Gene order is the same as in the phylogenetic tree in Figure 1. Quantitative data and statistical analysis are presented in Supplementary Table S4.

To examine the link between transcriptional regulation of maize *Pap* genes and the P deprivation response, we generated root and leaf transcriptomes of young maize seedlings under P sufficient and limiting conditions and quantified *Pap* transcripts. Plants were grown for 21 days post-emergence in an inert sand substrate supplemented with either 1000 μM (P+) or 10 μM (P–) Pi (see Materials and Methods). At harvest, P- plants were chlorotic and markedly smaller than P+ plants, presenting necrosis toward the tips of the oldest leaves (Figure 4). RNA was extracted from roots and leaves, and prepared for sequencing (see Materials and Methods). Mapping of sequencing reads to maize *Pap* gene models revealed a broad trend toward greater transcript accumulation under P limitation (Figure 5). This trend was most marked in leaf tissue, with 11 *ZmPap* transcripts accumulating to significantly higher levels under P− than P+ (Figure 5; Supplemental Table S4). As in the previous analysis of tissue and stage specific expression, transcripts of *ZmPap26* showed no significant difference in the level of accumulation between treatments, although the level of expression was relatively high (Figure 5; Supplemental Table S3). Transcripts encoded by the additional subgroup Ia-2 members *ZmPap10* and *ZmPap30a* were among those found to accumulate to higher levels under P-, consistent with an important role for this group under P deprivation (Figure 5; Supplemental Table S4). Transcripts of *ZmPap30b*, the final member of subgroup Ia-2, were not detected in seedling roots or leaves under either P regime, consistent with the previous tissue- and stage- specific analysis. Further transcripts accumulating to higher levels under P- included members of all the further maize *Pap* groups and subgroups (Figure 5; Supplemental Table S4).

### Promoter regions of strongly P-regulated *ZmPap* genes contain multiple putative PHR1 binding regions

Transcriptional regulation during the plant P deficiency response is a complex process that requires integration of a number of signaling pathways (Rouached et al., 2010). Nonetheless, certain key regulators have been identified, including the MYB transcription factor PHOSPHATE STARVATION RESPONSE 1 (PHR1) first identified in *Chlamydomonas* (Wykoff et al., 1999) and subsequently found to be conserved in *Arabidopsis* (Rubio et al., 2001) and rice (Zhou et al., 2008). In maize, two candidate gene models have been identified to encode putative PHR orthologs (Calderon-Vazquez et al., 2011). PHR1 binds the promoter region of its targets at an imperfect palindromic sequence GNATATNC, defined as the PHR1 Binding Sequence (P1BS; (Rubio et al., 2001; Zhou et al., 2008). P1BS elements have been identified previously in genomic regions 5′ of *OsPAP* coding sequences, and transcripts encoded by the genes related with these elements are found to accumulate to higher levels in *OsPHR2* over-expressing plants (Zhang et al., 2011). To investigate the potential importance of PHR in the transcriptional regulation of maize *Pap* genes, we examined a region 2500 bp upstream of the translational start site of each *ZmPap* for the presence of candidate P1BS elements (Table 1). One or more P1BS elements were identified within 2500bp upstream of 19 of 33 *ZmPaps*. For 15 of these 19 *ZmPaps*, P1BS elements were identified within 1000 bp upstream. A correlation was observed between the presence of P1BS elements and increased transcript accumulation under low P availability (Table 1; Figure 5). Four P1BS sites were found upstream of *ZmPap7d and ZmPap21c*, whose transcripts were strongly induced in response to low P. Similarly, two P1BS were present upstream of each of the genes *ZmPap10* and *ZmPap21d*, both encoding transcripts also induced under low P availability. The gene *ZmPap24b* was remarkable for a strong increase in transcript accumulation under low P, but absence of upstream P1BS sites. In certain instances, pairs of genes encoding closely related proteins differed in both the presence of P1BS and the pattern of transcript accumulation. For example, a P1BS sequence present proximal to the ATG (−256 bp) in *ZmPap30a* is absent from the closely related sequence *ZmPap30b;* accumulation of *ZmPap30a* transcripts was increased under low P in both roots and shoots, while no reads aligning to *ZmPap30b* transcripts could be detected in our transcriptome data (Supplementary Table S4).

### PAP encoding genes co-localize with genetically mapped AP loci

**Figure 6:**
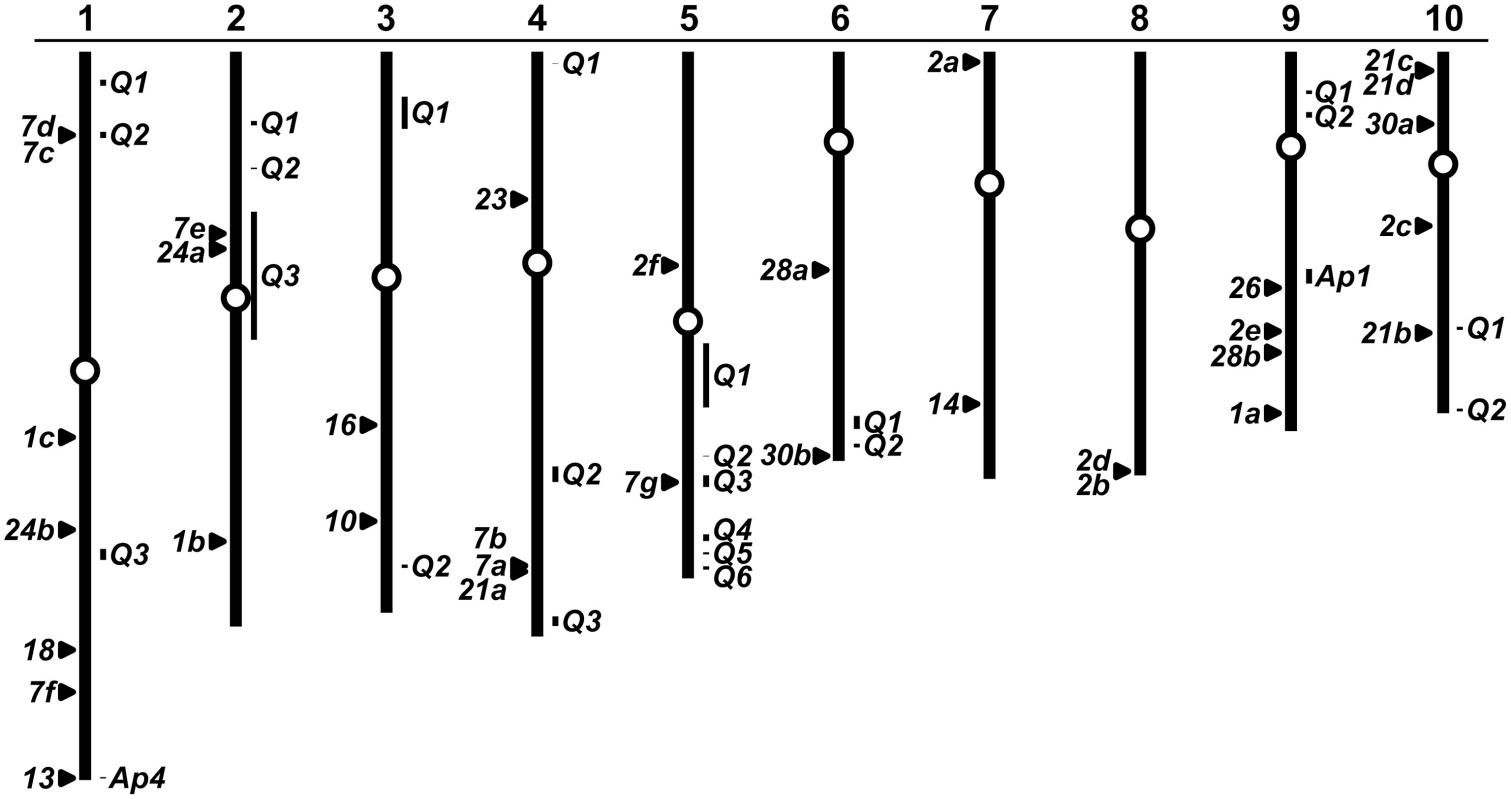
**Genomic location of *ZmPap* genes**. Physical map of the 10 chromosomes of maize (re-drawn from www.maizesequence.org) indicating the position of *ZmPap* genes (numbered filled triangles to the left of the chromosomes). Bars (to the right of the chromosomes) indicate approximate physical positions of the genetically mapped loci *AP1* and *AP4* (AP; maizegdb.org; Efron, 1971; Kahler, 1983) and consensus QTL (cQTL) for low P tolerance (Q numbers refer to cQTL presented in Zhang et al., 2014). Unfilled circles represent centromeres. Where multiple *ZmPap* loci lie in close proximity, triangles have been slightly displaced or labeled with multiple gene names.

A number of studies have been conducted previously to map the genetic basis of variation in the maize P deficiency response, including the secretion of AP activity to the soil under low P availability (for example, Zhu et al., 2005b, 2006; Chen et al., 2008). Efforts to quantitatively map maize tolerance to low P have recently been summarized by a meta-analysis that identified 23 consensus QTL (cQTL; Zhang et al., 2014). To investigate a possible link between maize *Pap* genes, known AP loci and reported cQTL, we mapped the maize *Pap* genes onto the maize physical map (Figure 6). Maize *Pap* loci were located on all 10 chromosomes. The *ZmPap26* locus was found to co-localize with the interval on chromosome 9 defined by the genetic position of the isozyme locus *Ap1* (Efron, 1971, 1973). Similarly, the locus *ZmPap13* co-localizes with the isozyme locus *Ap4* in the telomeric region of the long-arm of chromosome 1 (Kahler, 1983). The authors of the cQTL analysis noted previously the co-localization of *ZmPap21b* (GRMZM5G831009) with a cQTL on chromosome 10 (Figure 5; Zhang et al., 2014). We identified five further *ZmPap* genes to be within or close to cQTL intervals identified by Zhang et al.: *ZmPap7d*, *ZmPap7c* (cQTL1-2); *ZmPap7e*, *ZmPap24a* (cQTL2-3); *ZmPap7g* (cQTL5-3).

## Discussion

The maize (*Zea mays* ssp. *mays* var. B73) genome was found to contain 33 putative PAP encoding genes, a number marginally higher than that identified in the *Arabidopsis* and rice genomes (Li et al., 2002), and comparable to that of soybean (Li et al., 2012). All three PAP groups described previously in *Arabidopsis* (Li et al., 2002) were present in maize although, in common with rice and soybean, the subgroup Ia-1 was found to be absent (Figure 1; Li et al., 2002, 2012). The *Arabidopsis* subgroup Ia-1 consists of five members (*AtPAP11*, *AtPAP19*, *AtPAP6* and *AtPAP25*) encoding oligomeric high-molecular-weight PAPs (Li et al., 2002). AtPAP25 is exclusively synthesized under conditions of low P availability and has been hypothesized to function as a phosphoprotein phosphatase (Del Vecchio et al., 2014). The significance of the absence of the *Arabidopsis* subgroup Ia-1 in other plant species is not evident at this time. In contrast to the absence of subgroups Ia-1, the subgroup Ia-2 was found to be expanded in maize, notably by the presence of a distinct pair of proteins that, in our analysis, were identified only in grass species, being absent from both *Arabidopsis* and canola (Figure 2). In *Arabidopsis*, subgroup Ia-2 has been identified to play a key role in the P deprivation response (Li et al., 2002; Tran et al., 2010b; Wang et al., 2011). In maize, accumulation of transcripts encoding the subgroup Ia-2 PAPs was induced in seedling roots and leaves under low P availability (Figure 5), consistent with a role under P deprivation. Interestingly, transcripts encoding *ZmPap30b* are largely restricted to male reproductive tissues, suggesting a specialized role for the ZmPAP30b protein.

Considering the gene family as a whole, maize *PAP* transcripts accumulate in all vegetative and reproductive tissues examined (Figure 3), as has been previously reported for *Arabidopsis PAPs* under standard growth conditions (Zhu et al., 2005a). A number of specific transcripts, encoding members of each of the PAP groups, were found to accumulate to higher levels under low P availability (Figure 5). It has been demonstrated previously, however, that AtPAP26, the major secreted AP activity in *Arabidopsis*, is regulated largely at the post-transcriptional level (Tran et al., 2010b) and it would be best to be cautious before inferring functional importance from patterns of transcript accumulation alone. Nonetheless, transcriptional regulation of diverse *ZmPap* family members by P availability suggests broad participation in the P deprivation response, possibly in a range of roles.

P re-mobilization during the growing season is an important determinant of PE. Furthermore, in part as a result of the confounding effects of PAE variation, PUE represents a relatively unexplored avenue for crop improvement (Rose et al., 2011; Veneklaas et al., 2012). Significantly, PAPs have been proposed to play a major role in the breakdown of P containing compounds in senescing leaves, facilitating re-mobilization of P to more active photosynthetic tissue (Gepstein et al., 2003; Robinson et al., 2012a; Shane et al., 2014). In maize, the leaf develops basipetally, generating a regular and continuous gradient of maturity from tip to base (Li et al., 2010), and, as a consequence, a potential gradient of P requirement and use. In light of this, we compared transcript accumulation data from whole P starved leaves with published transcriptomes prepared from different positions along the base-tip axis under standard growth conditions (Li et al., 2010). A number of *ZmPap* transcripts that showed differential accumulation between developing and mature leaves (Figure 3), have been reported to also exhibit differential transcript accumulation along the base-tip axis (Li et al., 2010; Sekhon et al., 2011). Notably, *ZmPap10* transcripts were reduced from base to tip, while accumulation of *ZmPap30a* transcripts was increased (Li et al., 2010).

Among the transcripts accumulating to higher levels under low P availability are *ZmPap1c* and *ZmPap2b*, which encode two of six ZmPAPs predicted to be targeted to mitochondria, indicating a possible role in scavenging of reactive oxidative species or in the modulation of carbon metabolism (Li et al., 2008; Sun et al., 2012). Variation in sub-cellular localization has been reported also for PAPs in common bean, further indicating functional divergence (Liang et al., 2012). Additional P-regulated transcripts included *ZmPap13*, part of the subgroup Ib-1 whose members have previously been implicated in P metabolism, Fe/Mn homeostasis, phytic acid breakdown and ascorbate biosynthesis, (Zhu et al., 2005a; Zhang et al., 2008; Kuang et al., 2009), and *ZmPap7c* and *ZmPap17d*, part of group IIIb that includes AtPAP17, a protein previously characterized to play a role in both P mobilization and metabolism of reactive oxygen species (Del Pozo et al., 1999). Notably, the accumulation of *ZmPap7c* in the leaves of plants grown under low P availability was the highest level observed for any transcript in this experiment. Collectively, these observations suggest a broader role for maize PAPs in cellular stress responses beyond P acquisition and remobilization.

Definition of the maize *Pap* family in the B73 reference inbred line provides the basis for future study of *Pap* diversity in further maize inbred lines, landraces and wild-relatives. The co-localization of the physical position of maize *PAP* genes and the genetically defined loci *Ap1* and *Ap4* suggests diversity of PAP sequence, and potentially post-translation modification, to be present in broader maize germplasm (Figure 6). Furthermore, co-localization of *ZmPap* genes with previously defined low-P tolerance cQTL is consistent with the presence of functionally important variation within the maize germplasm pool. The possibility that natural variation in *Pap* sequences presents a valuable resource for crop improvement merits further investigation.

## Concluding remarks

We have defined a family of 33 maize *Pap* genes which we predict, on the basis of transcript accumulation and similarity to proteins characterized in other plants, to be functionally diverse and to play a role in both the P deprivation response and more generally in maize stress responses and development. From what is known of the post-transcriptional regulation of PAPs in other plants, it is probable that the capacity for functional divergence is even greater than revealed by this first characterization. Furthermore, groups of closely related sequences present dramatically different patterns of transcript accumulation, illustrating a capacity for rapid adoption of new biological roles during the radiation of the maize *Pap* family. Ultimately, a more complete understanding of the roles of individual maize PAPs will require functional and biochemical analysis. Given the availability of a reference genome and the increasing availability of resources for reverse genetics, it is now feasible to conduct a functional genomics analysis of a maize gene family. On the basis of the characterization presented here, we have selected a number of candidate genes and initiated a program of reverse screening. We anticipate that functional characterization of maize PAPs will facilitate their use as direct targets of selection and manipulation, as well as valuable reporters of plant nutrient and stress status.

## Author contributions

EG and RC performed gene family identification and phylogenetic analyses. AA prepared plant material and sequencing libraries. LA, RC, and CA performed transcriptomic data analyses. EG, RC, SF, and RS designed the study and prepared the manuscript. All authors read and approved the final manuscript.

### Conflict of interest statement

The authors declare that the research was conducted in the absence of any commercial or financial relationships that could be construed as a potential conflict of interest.
