# Supplementary material for: The maize (Zea mays ssp. mays var. B73) genome encodes 33 members of the purple acid phosphatase family
Source: Front Plant Sci. 2015 May 19;6:341. doi: 10.3389/fpls.2015.00341 (PMC4436580; doi:10.3389/fpls.2015.00341)
Supplement: Supplementary file 1 [file Table1.DOC]

The maize (Zea mays ssp. mays var. B73) genome encodes 33 members of the purple acid phosphatase family

Eliécer González-Muñoz, Aida-Odette Avendaño-Vázquez, Ricardo A. Chávez Montes, Stefan de Folter, Liliana Andrés-Hernández, Cei Abreu-Goodger and Ruairidh J. H. Sawers

Laboratorio Nacional de Genómica para la Biodiversidad (LANGEBIO), Centro de Investigación y de Estudios Avanzados del Instituto Politécnico Nacional (CINVESTAV-IPN), Irapuato C.P. 36821, Guanajuato, México.

TABLE S1: PAP Protein sequences and alignments used to construct Figures 1 and 2

#Sequences and alignment used for Figure 1

>AtPAP1

MRESLVAILVTVISVLGAIHQVKSHEDQPLSGIAVHKITFGLNEKAYVKASPTVLGSNGQ

HSELVLVQYSSPKPSDDDWIGVFSPADFNASTCPGDNKMVQPPRLCSAPVKFQYANFSNP

RYTNTGTGSLKLQLINQRSDFSFALFSGGLLNPKLVAISNKVAFENPNAPVYPRLALGKE

WDEMTVTWTSGYGLNLAEPVVEWGVKGGERKLSPAGTLTFARNSMCGAPARTVGWRDPGY

IHTAFLKELWPNSKYTYRVGHRLSNGALIWSKEYQFKSSPFPGQNSVQQVVIFGDMGKAE

VDGSSEYNDFQRASLNTTKQLIKDLKKTDAVFHIGDICYANGYLSQWDQFIAQIEPIAST

VPYMIASGNHERVWPNSGSFYEGLDSGGECGVPAETMFYVPAQNRAKVWYSSDYGMFRFC

VADTEHDWREGTEQYNFIEHCLASVDRQKQPWLIFLAHRVLGYSSTYFYAEEGSFAEPMG

RESLQKLWQKYKVDIAIYGHAHNYERTCPVYQSVCTSHEKSNYKAPLNGTIHIVAGGGGA

GLAEFSDLQPNWSLFRDYDYGFLKLTAIDHSNLLFEYKKSSDGRVHDSFTISKDYRDILA

CAVDSCPATTLAS

>AtPAP2

MIVNFSFFLLLFVSVFVSSADSKATISISPNALNRSGDSVVIQWSGVDSPSDLDWLGLYS

PPESPNDHFIGYKFLNESSTWKDGFGSISLPLTNLRSNYTFRIFRWSESEIDPKHKDHDQ

NPLPGTKHLLAESEQLTFGSGVGMPEQIHLSFTNMVNTMRVMFVAGDGEERFVRYGESKD

LLGNSAAARGMRYEREHMCDSPANSTIGWRDPGWIFDTVMKNLNDGVRYYYQVGSDSKGW

SEIHSYIARDVTAEETVAFMFGDMGCATPYTTFIRTQDESISTVKWILRDIEALGDKPAM

ISHIGDISYARGYSWVWDEFFAQVEPIASTVPYHVCIGNHEYDFSTQPWKPDWAASIYGN

DGGGECGVPYSLKFNMPGNSSESTGMKAPPTRNLYYSYDMGTVHFVYISTETNFLKGGSQ

YEFIKRDLESVDRKKTPFVVVQGHRPMYTTSNEVRDTMIRQKMVEHLEPLFVKNNVTLAL

WGHVHRYERFCPISNNTCGTQWQGNPVHLVIGMAGQDWQPIWQPRPNHPDLPIFPQPEQS

MYRTGEFGYTRLVANKEKLTVSFVGNHDGEVHDTVEMLASGVVISGSKESTKIPNLKTVP

ASATLMGKSESNALWYAKGAGLMVVGVLLGFIIGFFTRGKKSSSGNRWIPVKNEET

>AtPAP3

MTYIYRDTKITTKSTIPFLIFFLFCFSNLSMATLKHKPVNLVFYVYNLIIIFSSHSSTAE

LRRLLQPSKTDGTVSFLVIGDWGRRGSYNQSQVALQMGEIGEKLDIDFVISTGDNFYDNG

LTSLHDPLFQDSFTNIYTAPSLQKPWYSGNHDYRGDVRAQLSPMLRALDNRWVCMRSFIV

NAEIVDLFFVDTTPFVDKYFIQPNKHVYDWSGVLPRQTYLNNLLKELDVALRESVAKWKI

VIGHHTIKSAGHHGNTIELEKHLLPILQANEVDLYVNGHDHCLEHISSVDSNIQFMTSGG

GSKAWKGGDVNYVEPEEMRFYYDGQGFMSVHVSEAELRVVFYDVFGHVLHHWKKTYKEAL

YFAS

>AtPAP4

MSSKFDIGSLSIVMTLLICFLLLSLAPKLEAELATVQHAPNPDGSISFLVIGDWGRHGLY

NQSQVALQMGRIGEEMDINFVVSTGDNIYDNGMKSIDDPAFQLSFSNIYTSPSLQKPWYL

VLGNHDYRGDVEAQLSPILRSMDSRWICMRSFIVDAEIAELFFVDTTPFVDAYFLSPQDQ

TYDWSGVSPRKSYLQTILTELEMGLRESSAKWKIVVGHHAIKSASIHGNTKELESLLLPI

LEANKVDLYMNGHDHCLQHISTSQSPIQFLTSGGGSKAWRGYYNWTTPEDMKFFYDGQGF

MSVKITRSELSVVFYDVSGNSLHKWDTSKMLDSDFYFPL

>AtPAP5

MSLETFPPPAGYNAPEQVHITQGDHNGRGMIISWVTSLNEDGSNVVTYWIASSDGSDNKS

VIATTSSYRYFDYTSGYLHHAIIKELEYKTKYFYELGTGRSTRQFNLTPPKVGPDVPYTF

GVIGDLGQTYASNQTLYNYMSNPKGQAVLFAGDLSYADDHPNHDQSKWDSYGRFVEPSAA

YQPWIWAAGNHEIDYAQSIGETQPFKPYKNRYHVPYRASQNKYTPQNSWLQDEFKKVNRS

ETPWLIVLVHAPWYNSNNYHYMEGESMRVTFEPWFVENKVDIVFAGHVHAYERSERVSNI

QYNITDGMSTPVKDQNAPVYITIGDGGNIEGIANIFTDPQPSYSAFREASFGHALLEIKN

RTHAHYTWHRNKEDEAVIADSIWLKNRYYLPEEETI

>AtPAP6

MKNLVIFAFLFLSITTVINGGITSKFVRQALPSIEMSLDTFPSPGGYNTPEQVHLTQGDH

DGRGMIVSWVTPLNLAGSNVVTYWIATNGSDVKPAKKRAHASTKSYRFYDYSSGFLHHAT

IKGLEYDTKYIYEVGTDKSVRQFSFTTPPKIGPDVPYTFGIIGDLGQTYASNETLYHYMS

NPKGQAVLFAGDLSYADDHPNHDQRKWDTWGRFMEPCAAYQPFIFAAGNHEIDFVPNIGE

PHAFKPYTHRYPNAYKASQSTSPLWYSVRRASAHIIVLSSYSAYGKYTPQYIWLEQELKN

VNREETPWLIVIVHSPWYNSNNYHYMEGESMRVMFESWLVNSKVDLVLSGHVHAYERSER

ISNIKYNITNGLSSPVKDPNAPIYITIGDGGNIEGIANSFVDPQPSYSAYREASFGHAVL

EIMNRTHAQYTWHRNQDNEPVAADSIMLHNRHFFPVEEIVSSNIRA

>AtPAP7

MKMHVCFSVILMFLSIFFINGALSKLERLKHPVKKKSDGSLSFLVIGDWGRKGGFNQSLV

AHQMGVVGEKLDIDFVISVGDNFYDDGLKGVNDPSFEASFSHIYTHPSLQKQWYSVLGNH

DYRGNVEAQLSKVLTQKDWRWFCRRSFVLSSGMVDFFFADTNPFVEKYFTEPEDHTYDWR

NVLPRNKYISNLLHDLDLEIKKSRATWKFVVGHHGIKTAGNHGVTQELVDQLLPILEENK

VDLYINGHDHCLQHIGSHGKTQFLTSGGGSKAWRGHVQPWDPKELKLYYDGQGFMSLHIT

HSKAKFIYYDVSGNVLHRSSLSKRSAHL

>AtPAP8

MDSLRDVKPIKLIFSIFCLVIILSACNSTAELPRFVQPPEPDGSLSFLVVGDWGRRGSYN

QSQVALQMGKIGKDLNIDFLISTGDNFYDDGIISPYDSQFQDSFTNIYTATSLQKPWYNV

LGNHDYRGNVYAQLSPILRDLDCRWICLRSYVVNAEIVDIFFVDTTPFVDRYFDEPKDHV

YDWRGVLPRNKYLNSLLTDVDVALQESMAKWKIVVGHHTIKSAGHHGNTIELEKQLLPIL

EANEVDLYINGHDHCLEHISSINSGIQFMTSGGGSKAWKGDVNDWNPQEMRFYYDGQGFM

SVYTSEAELRVVFYDGLGHVLHRWSTLKNGVYSDI

>AtPAP9

MIAAVYTLFFFFLLISSVYSKATISISPQTLNRSGDIVVIKWSGVESPSDLDWLGIYSPP

DSPHDHFIGYKFLSDSPTWQSGSGSISLPLTNLRSNYTFRIFHWTQSEINPKHQDHDHNP

LPGTRHLLTESNQLNFRFAVNRPEQIHLSYTDNINEMRVVFVTGDGEEREARYGEVKDKL

DNIAVARGVRYEIEHMCHAPANSTVGWRDPGWTFDAVMKNLKQGIRYYYQVGSDLKGWSE

IHSFVSRNEGSEETLAFMFGDMGCYTPYTTFIRGEEESLSTVKWILRDIEALGDDKPVIV

SHIGDISYARGYSWIWDEFFTQIEPIASKVPYHVCIGNHEYDWPNQPWKPDWAAYVYGKD

SGGECGVPYSVKFNMPGNSTEATGMVKGPQSRNLYYSYDMGSVHFVYISTETDFLKGGKQ

YSFLKSDLESVNRSKTPFVVVQGHRPMYTTSRKIRDAAIREKMIEHLEPLLVKNNVTVAL

WGHVHRYERFCAISNNTCGERWQGNPVHLVIGMAGKDSQPMWEPRANHEDVPIFPQPANS

MYRGGEFGYIRLVANKERLTLSYVGNHDGEVHDVVEILASGEVISGSDDGTKDSNFGSES

DFAVLWYIEGASVMVVGVIFGYFVGFLSRKKKESGVGSSNRSWIQVKNEET

>AtPAP10

MGRVRKSDFGSIVLVLCCVLNSLLCNGGITSRYVRKLEATVDMPLDSDVFRVPCGYNAPQ

QVHITQGDVEGKAVIVSWVTQEAKGSNKVIYWKENSTKKHKAHGKTNTYKFYNYTSGFIH

HCPIRNLEYDTKYYYVLGVGQTERKFWFFTPPEIGPDVPYTFGLIGDLGQSYDSNITLTH

YENNPTKGQAVLFVGDISYADTYPDHDNRRWDSWGRFAERSTAYQPWIWTTGNHELDFAP

EIGENRPFKPFTHRYRTPYRSSGSTEPFWYSIKRGPAYIIVLASYSAYGKYTPQYQWLEE

EFPKVNRTETPWLIVLMHSPWYNSYDYHYMEGETMRVMYEAWFVKYKVDVVFAGHVHAYE

RSERVSNIAYNVVNGICTPVKDQSAPVYITIGDGGNIEGLATKMTEPQPKYSAFREASFG

HAIFSIKNRTHAHYGWHRNHDGYAVEGDRMWFYNRFWHPVDDSPSCNS

>AtPAP11

MELSHLALVCAAIAFSSIFVVSQAGITSTHARVSEPSEEMSLETFPPPAGYNAPEQVHIT

QGDNAGRAMIISWVMPLNEDGSNVVTYWIASSDGSDNKNAIATTSSYRYFNYTSGYLHHA

TIKKLEYDPSKSRSRCSLHIRYYSDLGQTYASNQTLYNYMSNPKGQAVLFVGDLSYADDH

PNHDQRKWDSYGRFVEPSAAYQPWSWAAGNYEIDYAQSISETQPFKPYKNRYHVPYKASQ

STSPLWYSIKRASTYIIVLSSYSAYDKYTPQNSWLQDELKKVNRSETSWLIVLVHAPWYN

SNNYHYMEGESMRVTFEPWFVENKVDIVFAGHVHAYERSKRISNIHYNITDGMSTPVKDQ

NAPIYITIGDGGNIEGIANSFTDPQPSYSAFREASFGHALLEIKNRTHAHYTWHRNKEDE

AVIADSIWLKKRYYLPEEETA

>AtPAP12

MSSRSDLKIKRVSLIIFLLSVLVEFCYGGFTSEYVRGSDLPDDMPLDSDVFEVPPGPNSP

QQVHVTQGNHEGNGVIISWVTPVKPGSKTVQYWCENEKSRKQAEATVNTYRFFNYTSGYI

HHCLIDDLEFDTKYYYEIGSGKWSRRFWFFIPPKSGPDVPYTFGLIGDLGQTYDSNSTLS

HYEMNPGKGQAVLFVGDLSYADRYPNHDNNRWDTWGRFVERSVAYQPWIWTAGNHEIDFV

PDIGEIEPFKPFMNRYHTPHKASGSISPLWYSIKRASAYIIVMSCYSSYGIYTPQYKWLE

KELQGVNRTETPWLIVLVHSPFYSSYVHHYMEGETLRVMYEQWFVKYKVDVVFAGHVHAY

ERSERVSNIAYNIVNGLCEPISDESAPIYITIGDGGNSEGLLTDMMQPQPKYSAFREASF

GHGLLEIKNRTHAYFSWNRNQDGNAVAADSVWLLNRFWRAQKKTWLDAF

>AtPAP13

MVVKYTMSMSFFVIFASTVTIIVHGFPSTLDGPLNPVTAPLDPNLNPIAFDLPESDPSFV

KPISEFLLPEQISVSLSYSFDSVWISWVTGEYQIGEKDSAPLDPNCVQSIVQYREFDVRR

TRKQNATGHSIVYNQQYSSENGFMNYTSGIIHHVQLTGLKPNTLYRYQCGDPSLSAMSKE

YYFRTMPKSTSENYPHRIVVAGDLGLTYNTSTVLGHILSNHPDLVVLLGGFSYADTYLAN

KTKLDCSSCHCDQNGTSSDCGSCYSSGETYQPRWDYWGRFMEPLTANVPTMMVAGEHEIE

PQTENNLTFAAYSSRFAFPSNESADQYIWLESDLIKINRSETPWVVATWSLPWYSTFKGH

YREAESMRIHLEDLLYNYRVDIVFNSHVDAYERSNRVYNYTLDQCGPVYITTGAGGAGKL

ETQHVDDPGNIPDPSQNYSCRSSGLNSTLEPVKDETCPVKQPEYSAYRESSFGFGILEVK

NETHALWSWNRNQDLYYLAADVIHIVRQPEMCSVCN

>AtPAP14

MEETRRRFVISSVLSVSLIYLCLSTCHVSAFDFGRRQLRFNTDGRFKILQVSDMHYGFGK

ETQCSDVSPAEFPYCSDLNTTSFLQRTIASEKPDLIVFSGDNVYGLCETSDVAKSMDMAF

APAIESGIPWVAILGNHDQESDMTRETMMKYIMKLPNSLSQVNPPDAWLYQIDGFGNYNL

QIEGPFGSPLFFKSILNLYLLDGGSYTKLDGFGYKYDWVKTSQQNWYEHTSKWLEMEHKR

WPFPQNSTAPGLVYLHIPMPEFALFNKSTEMTGVRQESTCSPPINSGFFTKLVERGEVKG

VFSGHDHVNDFCAELHGINLCYAGGAGYHGYGQVGWARRVRVVEAQLEKTMYGRWGAVDT

IKTWKRLDDKNHSLIDTQLLWTKNTTLEPNFGFSCSTIPQH

>AtPAP15

MTFLLLLLFCFLSPAISSAHSIPSTLDGPFVPVTVPLDTSLRGQAIDLPDTDPRVRRRVI

GFEPEQISLSLSSDHDSIWVSWITGEFQIGKKVKPLDPTSINSVVQFGTLRHSLSHEAKG

HSLVYSQLYPFDGLLNYTSGIIHHVRITGLKPSTIYYYRCGDPSRRAMSKIHHFRTMPVS

SPSSYPGRIAVVGDLGLTYNTTDTISHLIHNSPDLILLIGDVSYANLYLTNGTSSDCYSC

SFPETPIHETYQPRWDYWGRFMENLTSKVPLMVIEGNHEIELQAENKTFEAYSSRFAFPF

NESGSSSTLYYSFNAGGIHFVMLGAYIAYDKSAEQYEWLKKDLAKVDRSVTPWLVASWHP

PWYSSYTAHYREAECMKEAMEELLYSYGTDIVFNGHVHAYERSNRVYNYELDPCGPVYIV

IGDGGNREKMAIEHADDPGKCPEPLTTPDPVMGGFCAWNFTPSDKFCWDRQPDYSALRES

SFGHGILEMKNETWALWTWYRNQDSSSEVGDQIYIVRQPDRCPLHHRLVNHC

>AtPAP16

MKKPSLFQIIIIVLSIPTTTGRTVGNLRVREGSPFKIAIFADLHFGEDTWTDWGPGQDVN

SVNVMSAVLDAETPDFVVYLGDVVTANNIAIQNASLFWDKAISPTRDRGIPWATLFGNHD

DASFVWPLDWLSSSGIPPLRCPAASDDDGCTFRGTTRVELIQEEIKSSNALSYSMISPKE

LWPSVSNYVLLVESSDHSKPPVALLYFLDSGGGSYPEVISNAQVEWFKTKSNTLNPYLRI

PELIFWHIPSKAYKKVAPRLWITKPCVGSINKEKVVAQEAENGMMRVLENRSSVKAVFVG

HNHGLDWCCPYKDKLWLCFARHTGYGGYGNWPRGSRILEISEMPFRIKTWIRMEDGSVHS

EVNLTYD

>AtPAP17

MNSGRRSLMSATASLSLLLCIFTTFVVVSNGELQRFIEPAKSDGSVSFIVIGDWGRRGSF

NQSLVAYQMGKIGEKIDLDFVVSTGDNFYDNGLFSEHDPNFEQSFSNIYTAPSLQKQWYS

VLGNHDYRGDAEAQLSSVLREIDSRWICLRSFVVDAELVEMFFVDTTPFVKEYYTEADGH

SYDWRAVPSRNSYVKALLRDLEVSLKSSKARWKIVVGHHAMRSIGHHGDTKELNEELLPI

LKENGVDLYMNGHDHCLQHMSDEDSPIQFLTSGAGSKAWRGDINPVTINPKLLKFYYDGQ

GFMSARFTHSDAEIVFYDVFGEILHKWVTSKQLLHSSV

>AtPAP18

MEKWGILLLVTLSVSIIFTSAAADDYVRPKPRETLQFPWKQKSSSVPEQVHISLAGDKHM

RVTWVTNDKSSPSFVEYGTSPGKYSYLGQGESTSYSYIMYRSGKIHHTVIGPLEADTVYY

YRCGGEGPEFHLKTPPAQFPITFAVAGDLGQTGWTKSTLDHIDQCKYAVHLLPGDLSYAD

YMQHKWDTFGELVQPLASVRPWMVTQGNHEKESIPFIVDEFVSFNSRWKMPYEESGSNSN

LYYSFEVAGVHAIMLGSYTDYDRYSDQYSWLKADLSKVDRERTPWLIVLFHVPWYNSNNA

HQHEGDEMMAEMEPLLYASGVDIVFTGHVHAYERTKRVNNGKSDPCGPVHITIGDGGNRE

GLARKYKDPSPEWSVFREASFGHGELQMVNSTHALWTWHRNDDDEPTRSDEVWLNSLVNS

GCLKKRPQELRKMLLEP

>AtPAP19

MGLNHLTLVCSAIALLSIFVVSQAGVTSTHVRVSEPSEEMPLETFPPPACYNAPEQVHIT

QGDHAGRGMIISWVTPLNEDGSNVVTYWIANSDGSDNKSALATTSSYRYFNYTSGYLYHA

TIKGLETLYNYMSNPKGQAVLFAGDLSYADDHPNHDQRKWDSYGRFVEPSAAYQPWIWAA

GNHEIDYAESIPHKVHLHFGTKSNELQLTSSYSPLTQLMDELKKVNRSETPWLIVLVHAP

WYNSNNYHYMEGESMRVTFEPWFVENKVDIVFAGHVHAYERSERISNIQYNITDGMSTPV

KDQNAPVYITIGDGGNIEGIANNFIDPQPSYSAFREASFGHAILEIKNRTHAHYTWHRNK

EDEFIPEAVIADSIWLKNRYYLREEETS

>AtPAP20

MVKVLGLVAILLIVLAGNVLSYDRQGTRKNLVIHPTNEDDPTFPDQVHISLVGPDKMRIS

WITQSSISPSVVYGTVSGKYEGSANGTSSSYHYLLIYRSGQINDVVIGPLKPNTVYYYKC

GGPSSTQEFSFRTPPSKFPIKFAVSGDLGTSEWSKSTLEHVSKWDYDVFILPGDLSYANM

YQPLWDTFGRLVQPLASQRPWMVTHGNHELEKIPILHSNPFTAYNKRWRMPFEESGSSSN

LYYSFNVYGVHIIMLGSYTDFEPGSEQYQWLENNLKKIDRKTTPWVVAVVHAPWYNSNEA

HQGEKESVEMKESMETLLYKARVDLVFAGHVHAYERFSRVYQDKFDKCGPVYINIGDGGN

LEGLATKYRDPNPEISLFREASFGHGQLVVENATHARWEWHRNDDDVSVEKDSVWLTSLL

ADSSCKI

>AtPAP21

MKKMKIFGFLISFSLFFLSPFVCQANYDSNFTRPPPRPLFIVSHGRPKFYPQQVHISLAG

KDHMRVTYTTDDLNVASMVEYGKHPKKYDKKTAGESTSYTYFFYNSGKIHHVKIGPLKPN

TKYYYRCGGHGDEFSFKTPPSKFPIEFAVAGDLGQTDWTVRTLDQIRKRDFDVFLLPGDL

SYADTHQPLWDSFGRLLETLASTRPWMVTEGNHEIESFPTNDHISFKSYNARWLMPHAES

LSHSNLYYSFDVAGVHTVMLGSYTPYESHSDQYHWLQADLRKVDRKKTPWLVVVMHTPWY

STNKAHYGEGEKMRSALESLLYRAQVDVVFAGHVHTYERFKPIYNKKADPCGPMYITIGD

GGNREGLALRFKKPQSPLSEFRESSFGHGRLRIIDHKRAHWSWHRNNDEMSSIADEVSFE

SPRTSSHCHSNRYRGEI

>AtPAP22

MKLFGLFLSFTLLFLCPFISQADVPELSRQPPRPIVFVHNDRSKSDPQQVHISLAGKDHM

RVTFITEDNKVESVVEYGKQPGKYDGKATGECTSYKYFFYKSGKIHHVKIGPLQANTTYY

YRCGGNGPEFSFKTPPSTFPVEFAIVGDLGQTEWTAATLSHINSQDYDVFLLPGDLSYAD

THQPLWDSFGRLVEPLASKRPWMVTEGNHEIEFFPIIEHTTFKSYNARWLMPHTESFSTS

NLYYSFDVAGVHTVMLGSYTDFDCESDQYQWLQADLAKVDRKTTPWVVVLLHAPWYNTNE

AHEGEGESMREAMESLLFNARVDVVFSGHVHAYERFKRVYNNKADPCGPIHITIGDGGNR

EGLALSFKKPPSPLSEFRESSFGHGRLKVMDGKRAHWSWHRNNDSNSLLADEVWLDSLST

SSSCWPSSRSNDEL

>AtPAP23

MTLLIMITLTSISLLLAAAETIPTTLDGPFKPLTRRFEPSLRRGSDDLPMDHPRLRKRNV

SSDFPEQIALALSTPTSMWVSWVTGDAIVGKDVKPLDPSSIASEVWYGKEKGNYMLKKKG

NATVYSQLYPSDGLLNYTSGIIHHVLIDGLEPETRYYYRCGDSSVPAMSEEISFETLPLP

SKDAYPHRIAFVGDLGLTSNTTTTIDHLMENDPSLVIIVGDLTYANQYRTIGGKGVPCFS

CSFPDAPIRETYQPRWDAWGRFMEPLTSKVPTMVIEGNHEIEPQASGITFKSYSERFAVP

ASESGSNSNFYYSFDAGGVHFVMLGAYVDYNNTGLQYAWLKEDLSKVDRAVTPWLVATMH

PPWYNSYSSHYQEFECMRQEMEELLYQYRVDIVFAGHVHAYERMNRIYNYTLDPCGPVYI

TIGDGGNIEKVDVDFADDPGKCHSSYDLFFFNSLNLSN

>AtPAP24

MARVLGVLLCLLALFSSSLCLDHANGRGDQALAQINVYETSLALDSSVKLHASPQVLGSQ

GEDTEWVNLAISNPKPTSDDWIGVFSPAKFDSGNCWPTSGGKEKTPYICSSPIKYMYCNS

HPDYMKSGNVTLKFQIINQRADVSFALFSNGVQEPHLLGVSNPVAFFNPKAPVYPRLALG

KNWDEMTVTWTSGYNIDEAVPFIEWSAKGLPARRSPAGTLTFNRNSMCGNPARGVGWRDP

GFFHTSFLKELWPNREYIYRLGHDLVNGSTIWSKNYTFVSSPYPGQDSKQRVIIFGDMGK

GERDGSNEYNDYQPGSLNTTDQVIKDLKDIDIVFHIGDLTYSNGYLSQWDQFTAQVQPIA

STVPYMIASGNHERDWPDTGSFYAGTDSGGECGVPAETMFYFPAENRAKFWYKTDYGMFR

FCVADSEHDWREGTEQYKFIENCLATVDRKTQPWLIFIAHRVLGYSTNDWYGKEGTFEEP

MGRESLQKLWQKYKVDLAFYGHVHNYERTCPIYESQCVNNDKDHYSGTFKGTIHVVVGGA

GSHLSPFSSLVPKWSLVRDYDFGFVKLTASDHSSLLFEYKKSSTGQVYDSFNISRDYRDV

LACTHDSCEPTTSAG

>AtPAP25

MRMNKILLVFVFLSIATVINSGTTSNFVRTAQPSTEMSLETFPSPAGHNAPEQVHIVQGD

YNGRGIIISWVTPLNLAGSNVVTYWKAVDGDVKPKKKRGHASTSSYRFYDYTSGFLHHAT

IKGLEYDTKYIYEVGTDGSVRQFSFTSPPKVGPDVPYTFGIIGDLGQTLASNETLYHYMS

NPKGQAVLFPGDLSYADDHPNHDQRKWDSWGRFVEPCAAYQTFIYAAGNHEIDFVPNIGE

PHAFKPYIHRYHNAYKASKSISPLWYSIRRASAHIIVLSSYSAYGKYTPQYVWLEQELKK

VNREETPWLIVMVHSPWYNSNNYHYMEGESMRAMFESWFVNSKVDLVLSGHVHSYERSER

VSNIKYNITNGLSYPVKDPSAPIYITIGDGGNIEGIANSFTDPQPSYSAYREASFGHAVL

EIYNRTHAYYTWHRNQDNEPVAADSIMLHNRYFFPVEELESGNTRA

>AtPAP26

MNHLVIISVFLSSVLLLYRGESGITSSFIRSEWPAVDIPLDHHVFKVPKGYNAPQQVHIT

QGDYDGKAVIISWVTPDEPGSSQVHYGAVQGKYEFVAQGTYHNYTFYKYKSGFIHHCLVS

DLEHDTKYYYKIESGESSREFWFVTPPHVHPDASYKFGIIGDMGQTFNSLSTLEHYMESG

AQAVLFLGDLSYADRYQYNDVGVRWDSWGRFVERSTAYQPWLWSAGNHEVDYMPYMGEVT

PFRNYLQRYTTPYLASKSSSPLWYAVRRASAHIIVLSSYSPFVKYTPQWHWLSEELTRVD

REKTPWLIVLMHVPIYNSNEAHFMEGESMRAAFEEWFVQHKVDVIFAGHVHAYERSYRIS

NVRYNVSSGDRYPVPDKSAPVYITVGDGGNQEGLAGRFTEPQPDYSAFREASYGHSTLDI

KNRTHAIYHWNRNDDGKKVATDEFVLHNQYWGKNIRRRKLKKHYIRSVVGGWIAT

>AtPAP27

MARNFLLVLLWFIVQVSSSHENGRGDQALSQIDIYAINLAQHHSAFIHVSPLVLGSQGQD

TEWVNVVISNPEPSSDDWVGVFSPAKFDSSSCAPTDDKEIAPFICSAPVKYMYAKSSPDY

MKTGNAVLKFMLINQRADFSFALFTGGLSNPTLVSVSNHVSFINPKAPVYPRLALGKKWD

EMTVTWTSGYNIGEAVPFVEWSRKGTRSRRSPAGTLTFTRNSMCGAPARTVGWRDPGFIH

TASLKDLWPNLKYTYRMGHELMNGSIVWSKNFTFKSSPYPGQDSLQRVIIFGDMGKGERD

GSNEYNDYQPGSLNTTDQLIKDLKNIDIVFHIGDITYANGYISQWDQFTAQVEPIASTVP

YMVASGNHERDWPDSGSFYGGKDSGGECGVPAETMFDFPAENKAKFWYSADYGMFRFCVA

DTEHDWREGSEQYQFIERCLASVDRRAQPWLIFIAHRVLGYSTNDWYGQEGSFEEPMGRE

SLQKLWQKYKVDIAFYGHVHNYERTCPIYQNQCMDNEKSHYSGAFKGTIHVVVGGAGSHL

SSFSSLKPKWSIFRDYDYGFVKLTAFDHSSLLFEYKKSSNGAVHDSFTIFREYRDVLACV

RDSCEPTTLAS

>AtPAP28

MNCSIGNWKHTVLYLTLIVSLLYFIESLISHKLHINYNKIRLKRSPNLPLRFRDDGTFKI

LQVADMHFGMGMITRCRDVLDSEFEYCSDLNTTRFLRRMIESERPDLIAFTGDNIFGSST

TDAAESLLEAIGPAIEYGIPWAAVLGNHDHESTLNRLELMTFLSLMDFSVSQINPLVEDE

TKGDTMRLIDGFGNYRVRVYGAPGSVLANSTVFDLFFFDSGDREIVQGKRTYGWIKESQL

RWLQDTSIQGHSQRIHVNPPALAFFHIPILEVRELWYTPFIGQFQEGVACSIVQSGVLQT

FVSMGNVKAAFMGHDHVNDFCGTLKGVWFCYGGGFGYHAYGRPNWHRRARVIEAKLGKGR

DTWEGIKLIKTWKRLDDEYLSKIDEQVLWETSDSFLK

>AtPAP29

MADNRRRRSLFDFLLFSVFLGLACLCLSPIPATAQRRKLRFSVNGEFKILQVADMHFANG

AKTQCQNVLPSQRAHCSDLNTTIFMSRVIAAEKPDLIVFTGDNIFGFDVKDALKSINAAF

APAIASKIPWVAILGNHDQESTFTRQQVMNHIVKLPNTLSQVNPPEAAHYIDGFGNYNLQ

IHGAADSKLQNKSVLNLYFLDSGDYSSVPYMEGYDWIKTSQQFWFDRTSKRLKREYNAKP

NPQEGIAPGLAYFHIPLPEFLSFDSKNATKGVRQEGTSAASTNSGFFTTLIARGDVKSVF

VGHDHVNDFCGELKGLNLCYGGGFGYHAYGKAGWERRARVVVVDLNKKRKGKWGAVKSIK

TWKRLDDKHLSVIDSQVLWNNSANKLVVR

>ZmPAP26 (AC211394.4_FGP004)

MRGWGLLVLSLHVLSCLVSGVASGRTSSYVRTEFPSTDIPLESEWFAIPKGYNAPQQVHI

TQGDYDGKAVIVSWVTPEEPGPSEVFYGKEKLYDQKAEGTTTNYTFYDYKSGYIHHCLVD

GLEYNTKYYYKIGSGNSAREFWFETPPAIDPDASYTFGIIGDLGQTFNSLSTLQHYEKTG

GQTVLFVGDLSYADRYEHNDGIRWDSWGRFVEHSTAYQPWIWNTGNHEIEYRPDLGETSV

FKPYLHRYMTPYLASKSSSPMWYAVRRASAHIIVLSSYSPFVKYTPQWLWLKNEFKRVDR

EKTPWLIVLMHSPMYNSNEAHYMEGESMRAAFEKWFVKYKVDLVFAGHVHAYERSYRISN

VNYNITSGNRYPVPDKSAPVYITVGDGGNQEGLASRFYNPQPDYSAFREASYGHSVLQLK

NRTHAIYQWNRNDDGNPVPADTVMFHNQYWTSSTRRRRLKKNHLHLEDLEDLISLL

>ZmPAP10 (GRMZM2G093101_P01)

MGRHGVDQIGAVAAFVWVTLLPLLLVCVVWPGAQAGHTSEYRRQLGSAIDMPLDADVFRP

PPGYNAPEQVHITQGNHDGTAMIISWVTTSEPGSSTVIYGTSEDNLNYTANGKHTQYTFY

NYTSGYIHHCTIKKLEFDTKYYYAVGIGQTVRKFWFLTPPKSGPDVPYTLGLIGDLGQSF

DSNVTLTHYESNAKAQAVLFVGDLSYADNYPYHDNVRWDTWARFVERSVAYQPWIWTAGN

HEIDFAPELGETKPFKPFSHRYPTPYKASGSTAPYWYSIKRASAYIIVLASYSAYGKYTP

QYKWLEAEFPKVNRSETPWLVVLMHAPWYNSYNYHYMEGETMRVMYEPWFVKYKVDVVFA

GHVHAYERTHRISNVAYNVVNGLCTPIPDQSAPVYITIGDGGNQEGLATNMSQPQPSYSA

FREASFGHAILDIKNRTHAYYTWHRNQDGSAVAADSMWFTNRYWEPTDDSADDFQ

>ZmPAP30a (GRMZM2G073860_P01)

MMGPLPLRRRVGVLLFLALVLLGGGGGADAGTTSSYRRKLEATVEMPLDADVFRVPPGYN

APQQVHITLGDQEGTAMIVSWVTASEPGNSTVAYGEDPARMERRADGAHTRYDYFNYTSG

FIHHCTLRNLKHATKYYYAMGFGHTVRTFWFTTPPKPGPDVPFKFGLIGDLGQTFDSNIT

LSHYESNGGDAVLYVGDLSYADNHPLHDNNRWDTWARFVERSVAYQPWVWTAGNHELDFA

PELGETTPFKPFAHRYPTPYRAAGSTEPFWYSVKVASAHVVVLASYSAYGKYTPQWAWLQ

AELARVDRKTTPWLVVLTHSPWYNSNNYHYMEGETMRVQFERWLVDAKVDLVLAGHVHSY

ERSHRVSNVAYDIVNGKSTPVRSADAPVYVTIGDGGNIEGIADNFTRPQPGYSAFREASF

GHATLDIKNRTHAYYSWHRNHDGAKVVADGVWFTNRYWMPTDDDTN

>ZmPAP30b (GRMZM2G077466_P01)

MSNVLRARRRLDLLQVVLFFVAVLLVVVADAGVTSQYRRKLEATVEMPLDADVFRVPPGY

NAPQQVHITLGDQEGTAMIVSWVTANELGSSTVMYSEASPDPEKMELRAEGTHTRYDYFN

YTSGFIHHCTLTNLKHSTKYYYAMGFGHTVRSFCFTTPPMPGPDVPFKFGLIGDLGQTFD

SNTTLSHYEANGGDAVLYVGDLSYADNHPLHDNTRWDTWARFVERSAAHQPWVWTAGNHE

LDLAPELGEHVPFKPFAHRYPTPFWYSVRVASAHVVVLASYSAYGKYTAQWEWLRAELAR

VDRAATPWLIVLVHSPWYSSNGYHYMEGETMRVQFERWIVAAKADLVVAGHVHAYERSHR

VSNVAYDIINARCTPVRTRDAPVYVTVGDGGNIEGIADNFTQPQPSYSAFREASFGHATL

EIRNRTHAYYAWHRNQDGAKVVADGVWLTNRYWMPTDDDIN

>ZmPAP18 (GRMZM2G174549_P01)

MAAPSPLPRFLILLLAVTSSAFAAAAATGAPVVGEDYVRPPPARCHRKALLSLFPWSKKE

ESAASSDPQQVHISLAGEKHMRITWVTNDNSVPSVVDYGTKESTYTMKSQGESTSYSYLL

YSSGKIHHVVIGPLEDNTIYYYRCGGQGPEFQFKTPPSQFPLSLAVVGDLGQTSWTTSTL

NHIKQCEHDMLLLPGDLSYADYMQHLWDSFGTLVEPLASNRPWMVTEGNHEKEHIPFFES

GFQSYNARWKMPYEESGSRSNLYYSFEVAGAHIIMLGSYTDYDDSSDQYAWLKADLAKVD

RKRTPWLIVLLHVPWYNSNWAHQGEGDSMMASMEPLLYAAHVDMVIAGHVHAYERAERVY

NSRPDPCGAVHITIGDGGNREGLARRYRNPKPAWSVFREASFGHGELKIVNSTHAHWTWH

RNDDEEPVRTDDVWINSLAGSGCIQEGSRDRESRKILMSP

>ZmPA21b (GRMZM5G831009_P02)

MRTTRCMLMVAQALVVVSALLAAAAAATAAEYVRPPPGRIILTEHTEPAAHPQQVHVSAV

GEKHVRVSWVTDDMRAQSVVDYGKASRNYTASATGEHTSYRYFLYSSGKIHHVSIGPLEP

STVYYYRCGKAGKEFSLRTPPAALPIELALVGDLGQTEWTASTLAHASKTGHDMLLVPGD

LSYADTQQALWDSFGRFVQRHASRRPWMVTQGNHEVEAPPLPVPAGSPPPFAAYGARWRM

PHEESGSPSNLYYSFGAAGGAVHVVMLGSYAPFNASSDQYRWLARDLAAVDRRATPWLVV

LLHAPWYNTNAAHQGEGEAMRKAMERLLFQARVDVVFAGHVHAYERFARVYDNEANPCGP

VYITIGDGGNREGLAFNFDKNHTLAPLSMTREASFGHGRLRVVNTTSAHWAWHRNDDADS

VVRDELWLESLAAKASCRQHADPAVVDWDDEL

>ZmPAP21c (GRMZM2G434170_P01)

MATAALALALLVLALPASLAVTSTYVRPPPRATLSSLLEDDAGADGQTPQQVHISLVGPD

KVRVSWITAADAPATVDYGTDPGQYPFSATGNTTAYSYVLYQSGSIHDAVIGPLQPSTNY

YYRCSGSSSRELSFRTPPAALPFRFVVVGDLGQTGWTESTLKHVAAADYDALLLPGDLSY

ADLVQPRWDSYGRLVEPLASARPWMVTQGNHEVERLPLLEPRPFKAYNARWRMPYDYAAA

DSVAAAPPSDDNLFYSFDVAGGAVHVLMLGSYADYAAGSAQLRWLRADLAALRRRGTPPA

FVLALVHVPWYSSNEAHQGEGDAMRDAMEALLYHGARVDAVFAGHVHAYERFHRVYAGKE

DPCGPVYVTIGDGGNREGLANKFIDPQPSISAFREASFGHGRLEVVNATHALWTWHRNDD

NQPVVADQVWINSLAANPTCNRSIKMM

>ZmPAP21a (GRMZM2G111425_P01)

MTPSVHADEYVRPPQRPLALMAHDKPASHPQQVHISAVGAHHIRITWITDDRSAPSVVDY

GTSPGQYDASETGYQATYQFLSYTSGAIHHVTIGPLEPSTTYYYRCGSAGDEFSFRAPPA

TLPIDFVVIGDVGQTEWAASTLSQIGAADHDMMLLPGDLSYADRQQVLWDSWGRLVQPLA

SARPWMVTEGNHEKETLRELGTVRRFVAYNARWRMPHEESGSRSNLYYSFDASGGAVHVV

MLGSYADLEEGWSEQHAWLRRDLAAVDRRRTPWLLVLMHVPWYNTNRAHQGEAEAMRRDM

ESLLYEARVDVVFACHTHAYERFARVYDKKANSQGPMYITIGDAGNNKAEKFMSGHELAH

LSLFREPSFGYGRLRIIDNRRAVWTWHRNNDKDAQVSDEVWLESLATSP

>ZmPAP23 (GRMZM2G014193_P01)

MATPTSTVTRGGNRHWHCTQVLPLLLLVPLCFALLVESGGIPTTLDGPFPPATRAFDRAL

RQGSNDVPLTDPRLAPRVQPPAPEQIALAASADADSLWVSWVTGRARVGSSNLAPLDPAA

AGSEVWYGERSAADAASYPHVVTGSAEVYSQLYPYPGLLNYTSGAIHHVRLRGLRPATRY

YYRCGDSSLPGGLSDEHSFTTLPATGAGCYPRRVAVVGDLGLTGNSTATVDHLARNDPSL

VLMVGDMTYANQYLTTGGKGVPCFSCSFPKAPIRESYQPRWDGWGRFMEPITSKIPLMVI

EGNHEIEPQGHGGEVTFASYLARFAVPSKESGSNTKFYYSFNAGGIHFIMLGAYIDYNRT

GVQYSWLEKDLQRVDRRATPWVVAAWHPPWYNSYSSHYQEFECMRQEMEELLYEYQVDIV

FSGHVHAYERMDRVFNYTLDPCGPIYIGIGDGGNIEKIDMDHADDPGKCPSPSDNHPEFG

GLCHLNFTSGPAKGKFCWDRQPEWSAYRESSFGHGILEVLNSTYALWTWHRNQDAYAENS

VGDQIYIVRQPDKCLLQPATPLEDALL

>ZmPAP13 (GRMZM2G134054_P01)

MRRGSLSLLLLAAVAAVAATAVPAEPASTLSGPSRPVTVAIGDRGHAVDLPDTDPRVQRR

VTGWAPEQIAVALSASPTSAWVSWITGDYQMGGAVEPLDPGAVGSVVRYGLAADALDHEA

TGESLVYSQLYPFEGLQNYTSGIIHHVRLQGLEPGTRYLYRCGDPAIPDAMSDVHAFRTM

PAVGPGSYPGRIAVVGDLGLTYNTTSTVDHLVRNRPDLVLLLGDVCYANLYLTNGTGADC

YSCAFAKSTPIHETYQPRWDYWGRYMEPVTSSIPMMVVEGNHEIEQQIHNRTFAAYSSRF

AFPSEESGSSSPFYYSFDAGGIHFVMLASYADYSRSGAQYKWLEADLEKVDRSVTPWLIA

GWHAPWYTTYKAHYREAECMRVEMEELLYAYGVDVVFTGHVHAYERSNRVFNYTLDACGP

VHISVGDGGNREKMATAHADEAGHCPDPASTPDPFMGGRLCAANFTSGPAAGRFCWDRQP

EYSAYRESSFGHGVLEVRNDTHALWRWHRNQDLHAAAAANVAADEVYIVREPDKCLAKTA

RLLAY

>ZmPAP21d (GRMZM5G881649_P01)

MATAALALLVLALPASLAVTSTYVRPPPRATLSLLQDAGSDGQTPQQVHISLVGPDKVRV

SWITAADAPATVDYGTASGQYPFSATGNTTSYSYVLYHSGSIHDAVIGPLQPSTTYYYRC

SGSASRDLSFRTPPAVLPFRFVVVGDLGQTGWTESTLKHVAAADYDALLLPGDLSYADFV

QPRWDSYGRLVEPLASARPWMVTQGNHEVERLPLLEPRPFKAYNARWRMPYDYAADGTPP

SDDNLFYSFDVAGGAVHVLMLGSYADYAAGSAQLRWLRADLAALRRRGTPPAFVLALVHA

PWYSSNKVHQGEGDAMRDAMEALLYHGASTRCSRATSTRTSGSTASTPARRIPAAPSTSP

SATAATGRG

>ZmPAP2d (GRMZM2G138698_P03)

MGLSGSGAVASIVFLGLCATVSCWPAPPPPEMLHESFAGKSEFRTVNRRRLSSCSNPSPY

LSINVSSGGAPLPDEAFLTVTVAGVLRPDADDWVAMITPCSSSVSGCPLSGVNYVQTGDL

AHLPLLCHYPVKAQYMKRDPGYLGCKTAACQKRDASGACSVRTCAATVTFHVINFRTDVE

FVLFSGGFRTPCVLKRSGALRFANPASPLYGHLSSTDSTATSMRLTWVSGDGRPQQVQYG

GGKSATSQVATFTRNDMCSSPLLPSPAKDFGWHDPGYIHTAVMTGLQPSQSYTYRYGSDS

VGWSDTNTFRMPPAAGSDETSFVIYGDMGKAPLDPSVEHYIQPGSISVVKAVAKEIQTGK

VNSVFHIGDISYATGFLVEWDFFLNLIAPLASRVPYMTAIGNHERDYAESGSVYVTPDSG

GECGVAYESYFRMPAVSKDKPWYSIEQGSVHFVVMSTEHKWSEMSEQYKWMNQDLSSVNR

SRTPWVIFIGHRPMYSSHVGIPVNVDLAFVASVEPLLLKHQVDLVFFGHVHNYERTCAIY

KNICKGKPKKDESGIDTYDNSKYTAPVHATVGAGGFSLDKFPRIVLNKWSLSRVSEFGYA

RVHATRGDMLVQFVSSSTMEVLDQFRFVKPDPARRLRNKPV

>ZmPAP2b (GRMZM2G138756_P02)

MMAFFVVQRGSSQLTPLLALALLGLLRCAAVSCSAAAAATTLLHRESYAGKSEFRTVNRK

PLGSCVDPSPYLAIDVGAAGPIPDEAFLQVTVSGVQRPDPSDWVAMITPSNSSVAGCPLS

EVNYVETGDLANLPLLCHYPVKAQYLTSDPGYLGCKNAGCGKRDASGACTARTCAATLTF

HVVNFRTDVEFVLFSGGFKAPCLLKRSGARRFANPASPLYGHLSSTDSKATSMRLTWVSG

DGNPQRVQYGDGKSSTSEVATFTQDDMCSISVLPSPAKDFGWHDPGYIHSAVMTGLQPSQ

SYTYRYGSDSVGWSDTVKFRTAPAAGSDELSFVIYGDMGKAPLDPSVEHYIQPGSVSVAK

AVAKEIQTGNVDSIFHIGDISYATGFLVEWDFFLHLITPLASQVPYMTAIGNHERDYASS

ASVYVTPDSGGECGVAYESYFPMPAVSKDKPWYSIEQGTVHFIVMSTEHEWSEKSEQYNW

MDEDLSSVDRSRTPWVIFIGHRPMYSSHGGILPNVDSNFVASVEPLLLNYQVDLVFFGHV

HNYERTCAVYQGNCKGMPTTDKSGIDVYDNSNYTAPVHVIVGAGGFSLDSFPNKGEAWSL

SRVSEFGYGKVHATRTDMLVQFVNSSSMEVRDQFRIVKGAPANKSLSLIIQ

>ZmPAP24a (GRMZM2G157027_P01)

MRPAEGAARLVCLLLLAAVAAGHAGVQPLARIAIHRARFALDASAAVRASPELLGTKGED

TAWVKVDLVTPHPSADDWVGVFSPSKFNASTCLGSHGSGPGPVICSAPIKYQFANYSSGY

GESGKGALQFQLINQRQDFSFALFTGGLSNPKLIAVSNAIAFANPKAPVYPRLAQGKSWN

EMTVTWTSGYESDEAYPFVEWGMKWSPPVRSAAGTVTFDRESVCGEPARSVGWRDPGFIH

TAFLTDLWPNKEYYYKIGHMLPDGSVVWGKLSSFKAPPFPGQKSLQRVVIFGDMGKAERD

GSNEYSNYQPGSLNTTDTLVKDLDNIDMVFHIGDITYANGYISQWDQFTQQVEEITSRVP

YMVASGNHERDWPNSGSFFNGTDSGGECGVVAETMYYTPTENRANYWYSADYGMFRFCVA

DSEHDWREGTEQYEFIESCLATVDRKKQPWLVFIAHRVLGYSSGFFYGVDGSFAEPMSRQ

SLQKLWQKYRVDLAFYGHVHNYERTCPVYEEQCMSSEKSHYSGTMNGTIHVVVGGGGSHL

SNFTAQVPPWSVYREMDYGFVKLTAFNYSSLLYEYKRSSDGQVYDSFTMHREYRDLLACV

KDSCPPTSPAT

>ZmPAP2c (GRMZM2G326625_P01)

MKGSSCNIFLYDATGMLCQRGDSVGWSDTVKFRTAPAAGSDELSFVIYGDMGKAPLGPSV

EHYIQPGSVSVAKAVAKEIQTGNVDSIFHIGDISYATGFLVEWDFFLHLITPLASQVPYM

TAIGNHERDYVNSASVYVTPDSGGECGVAYESYFPMPAVSKDKPWYSIEQGTVHFIVMST

EHEWSEKSEQYNWMDEDLSSVDRSRTPWVIFIGHRPMYSSYGVILPNVDSNFVASVEPLL

LNYQVDLVFFGHVHNYERTCAVYQGNCKGMPTSDKSGIDVYDNNNYTAPVHVIVGVGGFS

LDNFPNKGEAWSLSRISEFGYGKVHATRTDMLVQFVNSSSMEIRDQFRIVKGAPANKSLS

LII

>ZmPAP1c (GRMZM2G315848_P01)

MRAPLLLWAAATWLAVSAVAHPGRRGGGGGEQPLSRIAVESAVLAVDDAAHVRASPLVLG

LKGESSEWVDVEFFHPDPSSDDWIGVFSPADFSAAICEPENPRQSPPVLCSAPIKYQFAT

FKNDGYNKTGKGYLKLQLINQRGDFSFALFSGGLLKPKLIAVSNKVAFANPKAPVYPRLA

QGKSWNEMTVTWTSGYDITEAVPFVEWGEKGGRRFLAPAGTLTFDRNSMCGAPARTVGWR

HPGYIHTSYLKDLWPDSRYTYRLGHRLMNGTRVWSKSYSFRASPYPGQDSLQRVVIFGDM

GKAEADGSNEFNNFQPGSLNTTYQITSDIENIDMVVHIGDICYANGYLSQWDQFTAQIEP

IASTVPYMVGSGNHERDWPGTGSFYGNLDSGGECGVPAQTVFYTPAENRAKFWYATDYGM

FRFCVAHTEEDWRPGTEQYRFIERCLSSVDRQKQPWLVFLAHRVLGYSSCAYYESEGTFE

EPMGREALQELWQKYKVDLAFYGHVHSYERTCPVYQSQCVVDASDHYSGPFQATTHVVVG

GAGASLSEFAASKIQWSHFTDFDHGFVKLTAFNHSSLLFEYKKSRDGNVYDRFTISRDYR

DVLACSVDNCPRTTLAS

>ZmPAP1b (GRMZM5G868679_P01)

MKGRLLLLLWAWAAATWVSVSAVTRLGRGVAGEQPLSRIAVERVVLAVNDAAYVKASPLV

LGHKGENSEWADVEFFHPNPSDDDWIGVFSPANFSDAICEAENTGTPVLCTAPIKYQFAN

FENDGYNKTGKGYLKLQLINQREDFSFALFSGGLSKPKLISVSNKVAFANPKAPVYPRLA

QGKSWNEMTVTWTSGYDITEAVPFVEWGEKGGRRLLAPAGTLTFDRNSMCGSPARTVGWR

HPGYIHTSFLKDLWPDSPYTYRLGHRLMNGTRVWSKSYSFKASPYPGQDSLQRVVVFGDM

GKAEADGSNEFSDFQPGSLNTTYQIIRDLEDIDMVVHIGDICYADGYLSQWDQFTAQIEP

IASRVPYMIGLGNHERDWPGTGSFYGNLDSGGECGVPAQTVFYTPAENRAKFWYATDYGM

FRFCIANTEEDWRPGTEQYKFIEQCLSSVDRQKQPWLIFLAHRVLGYSSCTYYESEGTFE

EPMGREALQELWQKYKVDIAFYGHVHSYERTCPVYQSQCVVDGSDHYSGPFQATTHVVVG

GAGASILDSEFTTSKIQWSHFTDFDHGFVKLTALNHSSLLFEYKKSRDGNVYDHFTISRD

YRDILACSIDNCPRSTLAS

>ZmPAP2a (GRMZM2G106600_P01)

MYPENPHLRFLLFLAVAAVAAGGAAANTTLTASLSGNQIKIIWSGLPAPDGLDYVAIYSP

PSSRDRDFLGYLFLNGSASWRGGSGELSLPLLPTLRAPYQFRLFRWPAKEYSYHHVDHDQ

NPLPHGKHRVAVSADVSVGDPARPEQLHLAFADEVDEMRVLFVCGDRGERVVRYGLQKED

DKEWKEVGTDVSTYEQRHMCDWPANSSVAWRDPGFVFDGLMKGLEPGRRYFYKVGSDTGG

WSEIYSFISRDSEASETNAFLFGDMGTYVPYNTYIRTQSESLSTVKWILRDIEALGDKPA

FISHIGDISYARGYSWVWDHFFSQIEPIAANTPYHVCIGNHEYDWPSQPWKPWWATYGTD

GGGECGIPYSVKFRMPGNSILPTGNGGPDTRNLYYSFDSGVVHFVYMSTETNFVQGSDQH

NFLKTDLEKVNRSRTPFVVFQGHRPMYTSSDETRDAALKQQMLQNLEPLLVTYNVTLALW

GHVHRYERFCPMKNSQCVNTSSSFQYSGAPVHLVIGMGGQDWQPVWQPRPDHPDVPIFPQ

PERSMYRGGEFGYARLVATREKLTLTYVGNHDGQVHDMVEIFSGLVSPSNSSVAEAVDGT

KLGTGVSTVRKISPLYLEIGGSVMFALLLGFSFGILVRRKKEAAQWTQVKNEES

>ZmPAP1a (GRMZM2G386998_P01)

MVALAQFARQVRPSPICNLDVTLWFHNLLARFYSTKMVYRKREKSVCWSQPSRMLSLPRT

AAAVTVYALVALIAGAAAAGGGEQPLSRIAIHRATVAPQPGAFVDASPALLGREGEDREW

VTVTYSNPRPSKDDWIGVFSPANFNDSICPPENEWVEPPLLCTAPIKFQFANYTNRDYGN

TGKGSLRLQLINQREGFSFALFSGGLSNPKLIAHSKSVTFINPKTPVYPRLAQGKSWNEI

TVTWTSGYGTNEATPFVRWGIEGQIQTLSPAGTLTFSRDTMCGPPARTVGWRDPGFIHTS

FLKDLWPNLLYTYQVGHRIFNGSIVWGHQYSFKAPPYPGEDSLQRVVILGDMGKAEVDGS

NEFNDFEPGSLNTTNQLIKDLKNIDVVFHIGDITYANGYLSQWDQFTAQVEPIASTVPYM

VGSGNHERDWPGSGSFYGNLDSGGECGVPAQNMFYVPAENREQFWYSTDYGMFRFCVANT

ELDWRPGTEQYRFIEHCLSSVDRQKQPWLIFLAHRVLGYSSATFYADEGTTEEPMGRESL

QPLWQKYKVDIAMYGHVHGYERTCPVYENACVAKGSDLYAGAFTATTHVVVGGGGASLAD

YTAARARWSHVRDRDFGFVKLTAFNHTRLLLEYKKSRDGSVHDHFTISRDYRDVLACGVD

NCPSTTLAS

>ZmPAP24b (GRMZM2G136453_P01)

MGREAPAMAVALLAALVASAAMFMLAGTASASPAEGIQPLSKIAVHRATVEMQPSAYVRA

TPSLLGEQGEDTEWVTVKFGWKEPSEDDWIGVFSPSEFNSSATCPNPWPAEEPYLCTAPI

KYQFANYSANYIYWGKGSIRLQLINQRSDFSFALFTGGLSNPRLIAVSEPISFKNPKAPV

FPRLAQGTSHDEMTVTWTSGYAIDEAYPFVEWGALVAGGVRHTARAPAGTLTFNRGSMCG

EPARTVGWRDPGFIHTAFLRDLWPNKEYHYRIGHELPDGSVVWGKPYSFRAPPSPGQPSL

QRVIVFGDMGKAERDGSNEYAAYQPGSLNTTDALIADLDNYDIVFHIGDMPYANGYISQW

DQFTAQVAPITARKPYMVGSGNHERDWPDTAAFWDVMDSGGECGVPAETYYYYPAENRAN

FWYKVDYGMFRFCVGDSEHDWRVGTPQYDFIEHCLSTVDRKHQPWLIFATHRVLGYSSNA

WYAGEGSFEEPEGRENLQRLWQKYRVDIAFFGHVHNYERTCPMYQSQCMTSEKTHYSGTM

NGTIFVVAGGGGCHLSSYTTAIPKWSIYRDYDFGFVKLTAFNHSSLLFEYKKSSDSKVYD

SFTIDRDYRDVLRCVHDSCFPTTLAT

>ZmPAP2e (GRMZM2G007754_P01)

MEPDPVAAGASSSPARLALHLIYSSSFFPAGFLLCEKKQVTQLAWCASSPQMTWRLVEWD

FFLNLIAPVASRVPYMTAIGNHERDYVESGSVYVTPDLGGECGVAYESYFCMPAISKDKP

WYSIEQGSVHFVVMSTEHKWSEMSEQYKWMNQDLSSVNRSRTPWIIFIGHRPMYSSHVGI

PVNVDLTFVASVEPLLLKHQVDLVFFGHVHNYERTCVVYKNRCKGKPKKDASGIDTYDNN

KYTAPVHATVRAGGFSLDKFPRIVLNKWSLSRVSEFGYARVHATRGDMLVQFKKPGDDMN

GDMASLSTLLANRKKGHFMKGDAVIVIKGDLKNLEGWVEKVEDETVHIRPKISDLPKTLA

FNEKELCKYFKPGDHVKVILGVQEGATGMVVKVEGHVLIILSDTTKEHVLCSSMEAWTNS

SMWSFRSSRKLSVLAGDFLLSRACVALAALGNTEVVSLMATAVEHLVTGETMQISTSRNK

RVFKRTVSGLVGALFNIILHLQSPYYTA

>ZmPAP2f (AC207043.3_FGP004)

MEPDLVAAGASSSPARLALHLIYSSSFFPAGFLLCEKKQVTQLAWCASSPQTTWRLVEWD

FFLNLIAPVASRVPYMTTIGNHERDYAETGSVYVTPDSGGECEVAYESYFCMPAVSKDKP

WYSIEQGSVHFVVMSTEHKWSEMSEQYKWMNQDLSSVNRSRTPWIIFIGHRPMYSSHVGI

PVNVDLTFVASVEPLLLKHQVDLVFFGHVHNYERTCAVYKNRCKGKPKKDASGIDTYDKC

KYTAPVHATVRAGGFSLDKFPRIVLNKWSLSRVSEFGYARVHATRGDMLVQFVSSRTMEV

LDQFRIVKPDPARRLRNKPV

>ZmPAP7d (GRMZM2G152447_P01)

MANRSVAMGLALAFVAMTALCCAPGAAELPRLDHPARSDGSLKLLVVGDWGRKGTHNQSR

VADQMGRVGEKLDIDFVISTGDNFYKNGLKGVRDQAFEESFVDIYTAQSLQKPWYSVLGN

HDYRGNALAQLSPVLRKIDDRFICMRSFIVNAELVDFFFVDTTPFQLEYWTHPGKHRYDW

RGVAPRGNYLANLLKDLDVAMKKSTARWKIVVGHHTMRSVSEHRDTEELLELLLPVLKDN

GVDFYINGHDHCLEHISSRDSPLQYFTSGGGSKAWRGVFHPNKDKLRFFYDGQGFMSLQL

NQDQAHFIFYDVFGNILYRWSSRHPQSSTYLDEE

>ZmPAP7c (GRMZM2G152477_P01)

MAQGSRSAAVPAAASFFALLAVAAPLLLCCAPAAAELARLEHPPKDGGSLSLLVVGDWGR

KGTFNQSRVAHQMGRVGEQLSIDFVISTGDNFYENGLTGTDDEAFEQSFTDIYTAKSLQK

PWYLVLGNHDYRGDALAQLSPVLRKIDSRFICIKSFVVNAEIVEFFFVDTTPFQLKYWTH

PKDDHYDWRGVAPRENYINNLLKDLDGAMKTSTAAWKVAVGHHTMRSVSDHGDTKELLQL

LLPVLQANGVDFYINGHDHCLEHISSRDSPIQYFTSGGGSKAWRGVQNPTEDDLKFFYDG

QGFMSLQLDRSQAKFTFYDVDGKALYSYTRSSLMETGHHLQASGYVNEE

>ZmPAP7e (AC202435.3_FGP003)

MGNSLVTATYCDLNPMAMQVVQGKWPPPVSYTVIVARLTQHALAKSQPAREQPPYQAGKR

NCVKEIPHLLTKEYITCLALICLVYLLPVVPYQHFSQELKQKHNDRTENEVAESNDWMSP

GYANAGSSPVPTPPSGKGLKASTKPKATKGQKSGPQTPLGFGSPGNPSTPVGGCRYDSSL

GYCCLLGLLTKFLNLLKGAPGGIVDLNNAAETLELITAEIGVDDSRHGEVSDDMSILQMG

RVGEKLDIDFVISTGDNFYKNGLKGVHDQAFKESFMDIYTAQSLQKPWYSVLGNHDYRGN

ALAQLSPVLRKIDDRFICMRSFIVNAELVDFFFIDTTPFQLEYWTHPGKHRYDWRGVAPR

GKYIANLLKDMDVAMKRSTARWKIVVGHHTMRSVSEHGDTEELLELLLPVLKDNGVDFYI

NGHDHYLEHISSRDSPLQYFTSGGGSKAWRGVFHPNKDKLRFFYDGQGFMSLQLNQDQAH

FIFYDVFGNILYRWSSRHPQSSTYLDEE

>ZmPAP7a (GRMZM2G141584_P01)

MARVASLLLTVIVAAAASLLLLLAPSAAELARVEHPPKNDGSLAILAVGDWGRRGQFNQT

LVAQQMGVVGEKLDIDFVISTGDNIYDDGIANTSDPLFKECFTNIYTAQSLQTPWYIVLG

NHDYTGNALAQQDPAIREVDSRYLNLAKSFIVNSGIADFFLLDTSPFYLKYWNSSKYDWR

DVAPRDTYIQNLLDDLDDALVQSEAPWKIVVGHHPISSGCEHGNTTELQELLRPILEARG

VDMYVNGHDHCLQHISSRNSPVQFMTSGGGSKAWAGKFKTTSDKIEFIYDGQGFMSMQLS

STEAHLVFYDVAGNVLHSYDSTKNEEEDDGWPAGQQAAVRGSIDRGNL

>ZmPAP7f (GRMZM2G351232_P01)

MHGVTLIPGVPEKDYARARYRYQNLSKLLPLFLTEQSLHEAKADPAKATTNGVPNGTGHS

YSGTVPSQQNSALTPDGNYRAPLLGGVPSAGYLDTTYGYDSTWAQFAWYDGSAYANGQQR

TTTTNRFPSSSFSGNGSSARYQNKSSTTQQMLHSCFCPLCIVFKQAPTYPNRVYPSPRPY

TQYENSVKTGLPYGSNGYDSRIYGRCGLGMDNRYRPRACSGYYGYGNESQDGTIELNRGP

RSGRFKNQKLYGHTVTIDPVMVQKRRIYDITNVLEGIGLIEKKLKNNIRWKGVDDSRPGE

FSDDMSILQMGRVGEKLDIDFVISTGDNFYKNGLKGVRDQAFKELFMDIYIAQSLQKPWY

SELVDFFFVDTTPFQLEYWTHPGKHRYDWRGVAPRGKYIANLLKDMDVAMKRSTARWKIV

VGHHTMRSVSEHGDTEELLELLLPVLKDNGVDFYINGHDHCLEHISSRDSPLQYFTSGGG

SKAWRGVFHPNKDKLRFFYDGQGFMSLQLNQDQAHFIFYDVFGNILYRWSSRHPQSSTYL

NEE

>ZmPAP7g (GRMZM2G404769_P01)

MNRKCLYSRLGTTTLSLRTMKAQISNGRGGCLALKLAEHEGRGGNLSKLLPLFLTEQSLH

EAKADPAKATANGVPNGTGHSYSGTVPSQQNSALTPDGNYRAPLLGGVPSAGYLDTTYGY

DSTGAQFAWYDGSAYANGQQRTTTTNRFPSSSFSGNGSSARYQNKSSTTQQMGMQNRRPT

TTSAAPTYPNRMYPSPRPYTQYGNSVKTGLPYGSNGYDSRINGRCGLGMDNRYRPRACSG

YYGYGNESQDGTIELNRGPRSGRFKNQKLYGHTVTIDPVMVQKRRIYDITNVLEGIGLIE

KKLKNNIRWKGVDDSRPGEFSDDMSILQMGRVREKLDIDFVISTGDNFYKNGLKGVRDQA

FKESFMDIYIAQSLQKPWYSELVDFFFVDTTPFQLEYWTHPGKHRYDWRGVAPRGKYIAN

LLKDMDVAMKRSTARWKIVVGHHTMRSVSEHGDTEELLELLLPVLKDNGVDFYINGHDHC

LEHISSRDSPLQYLTSRGGSKAWRGVFHPNKDKLRFFYDGQGFMSLQLNQDQTHFIFYDV

FGNILYRWSSRHPQSSTYLDEE

>ZmPAP7b (GRMZM2G109071_P02)

MGVVGQKLDIDFVISTGDNIYDDGIANTSDPLFKESFSNIYTANSLQKPWYLVLGNHDYT

GNALAQLDPAIRKVDSRYTAIAKSFIVNSGIADFFLVDTTPFIVHYWNNTKFDWRGVAPR

DTYIANLLKDLKCALTASKAPWKIVVGHHPISSACGHGNNTELEELLLPVLTTHGVDMYL

NGHDHCLQRVSSRDSRLQLLTSGGGSKAWAGKFKPTLDKVEFLYDGQGFMSMRLSRTEAR

LAFFDVAGSVLHCWTMHAQKLTTTTTAAAARH

>ZmPAP28a (GRMZM2G096363_P01)

MEVAERPMASSHLAALVSLLIPCLLALLLLRLAAVLDPDPDAAVPRIKAAAPLPLRFRHD

GAFKILQVADMHFGNGATTRCRDVGPEGGGARCSDLNTTRFLRRVIEAEKPDLIAFTGDN

IFGGSATDAAESLLRAISPAIEYRVPWAAILGNHDQESTMTREELMTFMSLMDYSVSQVN

PPGFLVHGFGNYHVGIHGPFGSELVNTSLLNLYFLDSGDREMVNGVKTYGWIRESQLAWL

RSTSLELQKKIHAPALAFFHIPIPEVRGLWYSGFKGQYQEGVACSSVHSGVLGTLVSMGD

VKSVFLGHDHLNDFCGNLNGIWFCYGGGFGYHAYGRPHWPRRARIIYSELKKGQRSWLEV

DSISTWKLLDDEKLSKIDEQVIWRHSTDDSDHRVYL

>ZmPAP14 (GRMZM2G109405_P01)

MGGCGLGRGRRTACLLLLLPPLLLFAVADAAASGKEKQGVSGKLRFRRESGTFKVVQVAD

MHYADGRSTACEDVLPSQVAGCTDLNTTAFLYRVFRAEDPDLVVFTGDNIYGADSTDAAK

SMDAAIAPAIDMKLPWAAVIGNHDQEGTLSREGVMRHLVGMKNTLASFNPEGIEIDGYGN

YNLEVSGVEGTSMDEKSVLNLYFLDSGDYSTVPSINGYGWIKASQQVWFQQTSSSLQAKY

MNKNPKQKEPAPGLVFFHIPLPEFSSFTASNFTGVKQEGISSASINSGFFASMVEAGDVR

AAFVGHDHINDFCGKLSGIQLCYAGGFGYHAYGKAGWSRRARVLSVQLEKTDSGEWRGVK

SIKTWKRLDDKHLSTIDSEVLWNRGSNGRRGKNPDGS

>ZmPAP16 (GRMZM2G366607_P01)

MHKWWRSPGPALLAAALAIVVAALRPATEHAAAKGRPPLRFGPGGAFKVALFADLHYGED

AWTDWGPAQDAASDRVMAAVLDAENPDLVVYLGDLVTANNLPVPNASLYWDRAVSAARGR

GVPWATVFGNHDDMAFEWPPEWFSPDGVPPLRWPPGPGSGCGFRGTPRTDLMAAETGANR

LLSYSSSGPRELWPGVSNYVLQVLSRGRRARGDGHDHDPALLMYFLDSGGGSYTEVVSSA

QVRWFHTQSQFLNPDGRIPELIFWHIPSTAYAKVAPKAKSEIRKPCVGSINEEEVAPQAA

EWGMMDALAKRSSVKAVFVGHNHGLDWCCPYDGEEREQELWLCFARHTGYGGYGDWPRGA

RILEVTEEPFSAVSWIRMENGTRHSDVTLTS

>ZmPAP28a (GRMZM2G104676_P01)

MQSATRLTLLLCAAWAAALLYGEMGAYWVSYLACSWPSSSSSPPNNHVKVAVVADPQLMD

STSLGLPSSSVALQAAEFYTDLNMRRSFQSTILPFKPDVVLFLGDHFDGGPYMPDEEWQE

SLFRFKHIFGLNDQRTKQQIPIYYLPGNHDIGYSAFHSVHPEVLSRYEKEFGSRNYQFSA

GKVDFVVVDAQTLDGAKQSKERSSSWEFIKTLSPGNTSNPKVLLTHIPLYRPDNSPCGPH

RSSPVINQRVSYAALDQGITYQNYLTKETSDLLLSLLKPILVLSGHDHDQCTVVHSTP

>AtPAP1

------------------------------------------------MRESLVAIL-----VTVISVLGAIHQVK---SHEDQPLSGIAVHKITFGLNEKAYVKASPTVLGSNGQHSELVLVQYSS-PKPSDDDWIGVFSPADFNASTCPGDN-KMVQPP------RLCSAPVKFQYANFSNPRY------------------TNTGTGSLKLQLINQRSDFSFALFSGGLLNPKLVAISNKVAFENPNAPVYPRL-ALGKEWDEMTVTWTSGYGLNL-----------AEPVVEWGVK--GGERK-----LSPAGTLTFA----------------------RNSMCG-----APARTVGWRDPGYIHTAFLKELWPNSKYTYRV-GHRLSN-------------GALIWSKEYQFKSSPFPGQN-SVQQVV--------------------IFGDMG---KAEVDGSSEYNDF---QRASLNTTKQLIKDLK------KTDAVFHIGDICYAN----------------------------------GYLSQWD-------QFIAQIEPIASTVPYMIASGNHE---RVWPNS------GSFYEGLDSGGE--C----------------GVPAETMFYVPAQNRA------------KVWYSS-------------------DYG----MFRFCVADTEHDWREG--TEQYNFIEHCLASVD----RQK---QP--WL---------IFLAHRVL-----------GY----SSTY-FYA--------------EEGSFAEPMGRESLQKLWQKY-KVDIAIYGHAHNYERTCPVYQSV-----CTSHEKSNYKA-------PLNGTIHIVAGGGGAG-L--AEFSDLQ-----------------------------------------PNWSLFRDYDYGFLKLTAIDHSNLLFEYKKSSD-----G------------------------------------RVHDSFTIS---------KDY---------------RDILAC----------------------------------------------AVDSCPATTLAS-----------------------------------------------------------------------------------

>AtPAP2

---------------------------------------------------------------------------------MIVNFSFFLLLFVSVFVSSADSKATISISPNALNRSGDSVVIQWSGVDSPSDLDWLGLYSPPESPNDHFIGYKFLNESSTWKDGFGSISLP-LTNLRSNYTFRIF--------RWSESEIDPKHKDHDQNPLPGTKHLLAESEQLTFGSGVGMPEQIHLS-----------------FTN-MVNTMRVMFVAGDGEER--------------FVRYG-----ESKDLLGNSAAARGMR-YE----------------------REHMCDS----PANSTIGWRDPGWIFDTVMKNLNDGVRYYYQV-GSD-----------------SKGWSEIHSYIARDVTAEETVAF-----------------------MFGDMG--------CATPYTTFIRTQDESISTVKWILRDIEALGD--KPAMISHIGDISYAR----------------------------------GYSWVWD-------EFFAQVEPIASTVPYHVCIGNHE---YDFSTQPWKPDWAASIYGNDGGGE--C----------------GVPYSLKFNMPGNSSESTGMKAPPTR--NLYYSY-------------------DMG----TVHFVYISTETNFLKG--GSQYEFIKRDLESVD----RKK---TP--FV---------VVQGH-RP-----------MY----TTSN-EVR--------------DTM--IRQKMVEHLEPLFVKN-NVTLALWGHVHRYERFCPISNNT--------------CG-----TQWQGNPVHLVIGMAGQD-W--QPIWQPRPNHPDLPIFPQ------------------------------PEQSMYRTGEFGYTRL-VANKEKLTVSFVGNHD-----G------------------------------------EVHDTVEML---------ASGVVISGSKESTKIPNLKTVPASATLMGKSESNALWYAKGAGLMVVGVLLGFIIGFFTRGKKSSS----GNRWIPVKNEET-----------------------------------------------------------------------------------

>AtPAP3

-----------------------------------------------------------------------------------------------------------MTYIYRDTKITTKSTIPFLI-----------------------------------------------------------------------------------------------------------------------------------------------------------------------------------------------------------------------------------------FFLFCFSNLSMATLKHKPVNLVFYVYN-----------------LIIIFSSHSSTAELRRLLQPSKTDGTVSFL--------------------VIGDWG-------------------RRGSYNQSQVALQMGEIGEKL-DIDFVISTGDNFYDN-----------------------------GLTSLHDPLFQD-------SFTNIYTAPSLQKPWYS--GNHD-----------------YRGDVRAQLS--P---------------MLRALDNRWVCMRSFIVNAEIV-------DLFFV--------------------DTTPFVDKYFIQPNKHVYDWSGV--LPRQTYLNNLLKELDVALRESV---AK--WK---------IVIGH--------------------HTIK-SAG--------------HHG--NTIELEKHLLPILQAN-EVDLYVNGHDHCLEHISSVDSNI----------------------------QFMTSGGGSKA-W--KGGDVNYVE---------------------------------------PEEMRFYYDGQGFMSVHVSEAELRVVFYDVFGH------------------------------------------VLHH--WKK---------------------------TYKEAL----------------------------------------------YFAS-------------------------------------------------------------------------------------------

>AtPAP4

----------------------------------------------------------------------------------------------------------MSSKFDIGSLSIVMTLLICFL------------------------------------------------------------------------------------------------------------------------------------------------------------------------------------------------------------------------------------------------------LLSLAPKLEAELAT------------------------------VQHAPNPD---GSISFL--------------------VIGDWG-------------------RHGLYNQSQVALQMGRIGEEM-DINFVVSTGDNIYDN-----------------------------GMKSIDDPAFQL-------SFSNIYTSPSLQKPWYLVLGNHD-----------------YRGDVEAQLS--P---------------ILRSMDSRWICMRSFIVDAEIA-------ELFFV--------------------DTTPFVDAYFLSPQDQTYDWSGV--SPRKSYLQTILTELEMGLRESS---AK--WK---------IVVGH--------------------HAIK-SAS--------------IHG--NTKELESLLLPILEAN-KVDLYMNGHDHCLQHISTSQSPI----------------------------QFLTSGGGSKA-W--RGYYNWTT----------------------------------------PEDMKFFYDGQGFMSVKITRSELSVVFYDVSGN------------------------------------------SLHK--WDT---------------------------SKMLDS----------------------------------------------DFYFPL-----------------------------------------------------------------------------------------

>AtPAP5

-------------------------------------------------------------------------------------------------------------------------------------------------------------------------------------------------------------------------MSLET--FPPPAGY-NAPEQVHIT-----------------QGDHNGRGMIISWVTSLNEDG------------SNVVTYWI-ASSDGSD----NKSVIATTSSY----------------------R---------------YFDYTSGYLHHAIIKELEYKTKYFYEL-GTG-------------------RSTRQFNL-TPPKVGPD-VPYTFG--------------------VIGDLG---------------------QTYASNQTLYNYMSNP----KGQAVLFAGDLSYAD------------------------------DHPNHDQSKWD-------SYGRFVEPSAAYQPWIWAAGNHE---IDYAQS-------------IGETQ--P----------------FKPYKNRYHVPYRASQ---------------------------------------------------------NKY--TPQNSWLQDEFKKVN----RSE---TP--WL---------IVLVH-AP-----------WY----NSNN-YHY--------------MEG--ES--MRVTFEPWFVEN-KVDIVFAGHVHAYERSERVSNIQYNITDGMSTPVKDQNA-----------PVYITIGDGG-N-I--EGIANIFTDPQ-------------------------------------PSYSAFREASFGHALLEIKNRTHAHYTWHRNKE-----D----------------------------------EAVIADSIWLK---------N-----------------RYYLPE----------------------------------------------EETI-------------------------------------------------------------------------------------------

>AtPAP6

----------------------------------------------------------------------------------------------------------------------MKNLVIFAF----------------------------LFLSIT------TVI--------NGGITSKF----------------------VRQALPSIEMSLDT--FPSPGGY-NTPEQVHLT-----------------QGDHDGRGMIVSWVTPLNLAG------------SNVVTYWI-A-TNGSDVKPAKKRAHASTKSY----------------------R---------------FYDYSSGFLHHATIKGLEYDTKYIYEV-GTD-------------------KSVRQFSFTTPPKIGPD-VPYTFG--------------------IIGDLG---------------------QTYASNETLYHYMSNP----KGQAVLFAGDLSYAD------------------------------DHPNHDQRKWD-------TWGRFMEPCAAYQPFIFAAGNHE---IDFVPN-------------IGEPH--A----------------FKPYTHRYPNAYKASQSTS---------PLWYSV-------------------RRA----SAHIIVLSSYSAYGKY--TPQYIWLEQELKNVN----REE---TP--WL---------IVIVH-SP-----------WY----NSNN-YHY--------------MEG--ES--MRVMFESWLVNS-KVDLVLSGHVHAYERSERISNIKYNITNGLSSPVKDPNA-----------PIYITIGDGG-N-I--EGIANSFVDPQ-------------------------------------PSYSAYREASFGHAVLEIMNRTHAQYTWHRNQD-----N----------------------------------EPVAADSIMLH---------N-----------------RHFFPV----------------------------------------------EEIVSSNIRA-------------------------------------------------------------------------------------

>AtPAP7

-----------------------------------------------------------------------------------------------------------------MKMHVCFSVILMFL-------------------------------------------------------------------------------------------------------------------------------------------------------------------------------------------------------------------------------------------------------------SIFFING-----------------------ALSKLERLKHPVKKKSD-GSLSFL--------------------VIGDWG-------------------RKGGFNQSLVAHQMGVVGEKL-DIDFVISVGDNFYDD-----------------------------GLKGVNDPSFEA-------SFSHIYTHPSLQKQWYSVLGNHD-----------------YRGNVEAQLS--K---------------VLTQKDWRWFCRRSFVLSSGMV-------DFFFA--------------------DTNPFVEKYFTEPEDHTYDWRNV--LPRNKYISNLLHDLDLEIKKSR---AT--WK---------FVVGH--------------------HGIK-TAG--------------NHG--VTQELVDQLLPILEEN-KVDLYINGHDHCLQHIGSHGKTQ-----------------------------FLTSGGGSKA-W--RGHVQPWD----------------------------------------PKELKLYYDGQGFMSLHITHSKAKFIYYDVSGN------------------------------------------VLHRSSLSK---------------------------RSAHL----------------------------------------------------------------------------------------------------------------------------------------------

>AtPAP8

---------------------------------------------------------------------------------------------------------------MDSLRDVKPIKLIFSI------------------------------------------------------------------------------------------------------------------------------------------------------------------------------------------------------------------------------------------------FCLVIILSACN--------------------------------STAELPRFVQPPEPD---GSLSFL--------------------VVGDWG-------------------RRGSYNQSQVALQMGKIGKDL-NIDFLISTGDNFYDD-----------------------------GIISPYDSQFQD-------SFTNIYTATSLQKPWYNVLGNHD-----------------YRGNVYAQLS--P---------------ILRDLDCRWICLRSYVVNAEIV-------DIFFV--------------------DTTPFVDRYFDEPKDHVYDWRGV--LPRNKYLNSLLTDVDVALQESM---AK--WK---------IVVGH--------------------HTIK-SAG--------------HHG--NTIELEKQLLPILEAN-EVDLYINGHDHCLEHISSINSGI----------------------------QFMTSGGGSKA-W--KGDVNDWN----------------------------------------PQEMRFYYDGQGFMSVYTSEAELRVVFYDGLGH------------------------------------------VLHR--WST-----------------------------LKNG----------------------------------------------VYSDI------------------------------------------------------------------------------------------

>AtPAP9

---------------------------------------------------------------------------------MIA--AVYTLFFFFLLISSVYSKATISISPQTLNRSGDIVVIKWSGVESPSDLDWLGIYSPPDSPHDHFIGYKFLSDSPTWQSGSGSISLP-LTNLRSNYTFRIF--------HWTQSEINPKHQDHDHNPLPGTRHLLTESNQLNFRFAVNRPEQIHLS-----------------YTD-NINEMRVVFVTGDGEER--------------EARYG-----EVKDKLDNIAVARGVR-YE----------------------IEHMCHA----PANSTVGWRDPGWTFDAVMKNLKQGIRYYYQV-GSD-----------------LKGWSEIHSFVSRNEGSEETLAF-----------------------MFGDMG--------CYTPYTTFIRGEEESLSTVKWILRDIEALGDD-KPVIVSHIGDISYAR----------------------------------GYSWIWD-------EFFTQIEPIASKVPYHVCIGNHE---YDWPNQPWKPDWAAYVYGKDSGGE--C----------------GVPYSVKFNMPGNSTEATGMVKGPQSR-NLYYSY-------------------DMG----SVHFVYISTETDFLKG--GKQYSFLKSDLESVN----RSK---TP--FV---------VVQGH-RP-----------MY----TTSR-KIR--------------DAA--IREKMIEHLEPLLVKN-NVTVALWGHVHRYERFCAISNNT--------------CG-----ERWQGNPVHLVIGMAGKD-S--QPMWEPRANHEDVPIFPQ------------------------------PANSMYRGGEFGYIRL-VANKERLTLSYVGNHD-----G------------------------------------EVHDVVEIL---------ASGEVISGS---------DDGTKDSNFGSESDFAVLWYIEGASVMVVGVIFGYFVGFLSRKKKESGVGSSNRSWIQVKNEET-----------------------------------------------------------------------------------

>AtPAP10

---------------------------------------------------------------------------------------------------------------MGRVRKSDFGSIVLVL----------------------------CCVLNS------LLC--------NGGITSRY----------------------VRKLEATVDMPLDSDVFRVPCGY-NAPQQVHIT-----------------QGDVEGKAVIVSWVTQ-EAKG------------SNKVIYWK----ENSTK---KHKAHGKTNTY----------------------K---------------FYNYTSGFIHHCPIRNLEYDTKYYYVL-GVG-------------------QTERKFWFFTPPEIGPD-VPYTFG--------------------LIGDLG---------------------QSYDSNITLTHYENNPT---KGQAVLFVGDISYAD------------------------------TYPDHDNRRWD-------SWGRFAERSTAYQPWIWTTGNHE---LDFAPE-------------IGENR--P----------------FKPFTHRYRTPYRSSGSTE---------PFWYSI-------------------KRG----PAYIIVLASYSAYGKY--TPQYQWLEEEFPKVN----RTE---TP--WL---------IVLMH-SP-----------WY----NSYD-YHY--------------MEG--ET--MRVMYEAWFVKY-KVDVVFAGHVHAYERSERVSNIAYNVVNGICTPVKDQSA-----------PVYITIGDGG-N-I--EGLATKMTEPQ-------------------------------------PKYSAFREASFGHAIFSIKNRTHAHYGWHRNHD-----G----------------------------------YAVEGDRMWFY---------N-----------------RFWHPV------------------------------------------------DDSPSCNS-------------------------------------------------------------------------------------

>AtPAP11

------------------------------------------------------------------------------------------------------------------MELSHLALVCAAI----------------------------AFSSIF------VVSQAGITSTHARVSEPSE--------------------------------EMSLETFPPPAGY-NAPEQVHIT-----------------QGDNAGRAMIISWVMPLNEDG------------SNVVTYWIASSDGSD-----NKNAIATTSSY-------------------------------------RYFNYTSGYLHHATIKKLEYD-----------------------------------------PSKSRSRCSLHIR--------------------YYSDLG---------------------QTYASNQTLYNYMSNP----KGQAVLFVGDLSYAD------------------------------DHPNHDQRKWD-------SYGRFVEPSAAYQPWSWAAGNYE---IDYAQS-------------ISETQ--P----------------FKPYKNRYHVPYKASQSTS---------PLWYSI-------------------KRA----STYIIVLSSYSAYDKY--TPQNSWLQDELKKVN----RSE---TS--WL---------IVLVH-AP-----------WY----NSNN-YHY--------------MEG--ES--MRVTFEPWFVEN-KVDIVFAGHVHAYERSKRISNIHYNITDGMSTPVKDQNA-----------PIYITIGDGG-N-I--EGIANSFTDPQ-------------------------------------PSYSAFREASFGHALLEIKNRTHAHYTWHRNKE-----D----------------------------------EAVIADSIWLK---------K-----------------RYYLPE----------------------------------------------EETA-------------------------------------------------------------------------------------------

>AtPAP12

------------------------------------------------------------------------------------------------------------MSSRSDLKIKRVSLIIFLL----------------------------SVLVE--------FC--------YGGFTSEY----------------------VRGSDLPDDMPLDSDVFEVPPGP-NSPQQVHVT-----------------QGNHEGNGVIISWVTP-VKPG------------SKTVQYWC----ENEKS---RKQAEATVNTY----------------------R---------------FFNYTSGYIHHCLIDDLEFDTKYYYEI-GSG-------------------KWSRRFWFFIPPKSGPD-VPYTFG--------------------LIGDLG---------------------QTYDSNSTLSHYEMNPG---KGQAVLFVGDLSYAD------------------------------RYPNHDNNRWD-------TWGRFVERSVAYQPWIWTAGNHE---IDFVPD-------------IGEIE--P----------------FKPFMNRYHTPHKASGSIS---------PLWYSI-------------------KRA----SAYIIVMSCYSSYGIY--TPQYKWLEKELQGVN----RTE---TP--WL---------IVLVH-SP-----------FY----SSYV-HHY--------------MEG--ET--LRVMYEQWFVKY-KVDVVFAGHVHAYERSERVSNIAYNIVNGLCEPISDESA-----------PIYITIGDGG-N-S--EGLLTDMMQPQ-------------------------------------PKYSAFREASFGHGLLEIKNRTHAYFSWNRNQD-----G----------------------------------NAVAADSVWLL---------N-----------------RFWRAQ----------------------------------------------KKTWLDAF---------------------------------------------------------------------------------------

>AtPAP13

-----------------------------------------------------------------------------------------------------------------MVVKYTMSMSFFVI----------------------------FASTVT------IIVHGFP-STLDGPLNPVT------------------APLDPNLNPIAFDLPESDPSFVKPISEFLLPEQISVS-----------------LSY-SFDSVWISWVTGEYQIGEKDSAPLDPNCVQSIVQYRE--FDVRRTR---KQNATGHSIVY----------------------NQQY-------SSENGFMNYTSGIIHHVQLTGLKPNTLYRYQC-GDPSL----------------SAMSKEYYFRTMPKSTSENYPHRIV--------------------VAGDLG---------------------LTYNTSTVLGHILSN-----HPDLVVLLGGFSYADTYLA-NKTKLDCSSCHCDQNGTSSDCGSCYSSGETYQPRWD-------YWGRFMEPLTANVPTMMVAGEHE-----------------IEPQTENNLT----------------------------------------------------------------------------------FAAYSSRFAFPSNESADQYIWLESDLIKIN----RSE---TP--WV---------VATWS-LP-----------WY----STFK-GHY--------------REA--ES--MRIHLEDLLYNY-RVDIVFNSHVDAYERSNRVYNYT-----------LDQCG-----------PVYITTGAGGAGKLETQHVDDPGNIPDPSQNYSCRSSGLNSTL--EPVKDETCPVKQ-------PEYSAYRESSFGFGILEVKNETHALWSWNRNQD---------------------------------------LYYLAADVIHIV---------------------------RQPEMC------------------------------------------------SVCN-----------------------------------------------------------------------------------------

>AtPAP14

----------------------------------------------------------------------------------------------------------MEETRRRFVISSVLSVSLIYL------------------------------------------------------------------------------------------------------------------------------------------CLSTCHVSAFDFGR-------------------------------------------------------------------------------RQLRFNTDGRFKILQVSDMHYGFGKETQC--------------------------------------SDVSPAEFP--------------------YCSDLN-------------------------TTSFLQRTIASE----KPDLIVFSGDNVYGL--------------------------------CETSDVAKS-------MDMAFAPAIESGIPWVAILGNHD--QESDMTRETMMKYIMKLPNSLSQVN--P------PDAWLYQIDGFGNYNLQIEGPFGSPLFFKSIL------NLYLL--------------------DGG---SYTKLDGFGYKYDWVK---TSQQNWYEHTSKWLE----MEH---KR--WPFPQNSTAPGLVYLH-IPMPEFAL----FNK----STEMTGVR--------------QESTCSPPINSGFFTKLVERG-EVKGVFSGHDHVNDFCAELHGIN-------------------------------LCYAGGAG-Y--HGYGQVGW----------------------------------------ARRVRVVEAQLEKTMYGRWGAVDTIKTWKRLDD-------------------------------------KNHSLIDTQLLWTK---------NTTLEP------------NFGFSC----------------------------------------------STIPQH-----------------------------------------------------------------------------------------

>AtPAP15

---------------------------------------------------------------------------------------------------------------------MTFLLLLLFC----------------------------FLSPAI------SSAHSIP-STLDGPFVPVT------------------VPLDTSLRGQAIDLPDTDPRVRRR-VIGFEPEQISLS-----------------LSS-DHDSIWVSWITGEFQIG-KKVKPLDPTSINSVVQFGT----LRHSL---SHEAKGHSLVY----------------------SQLY-------PFD-GLLNYTSGIIHHVRITGLKPSTIYYYRC-GDPS----------------RRAMSKIHHFRTMPVSSPSSYPGRIA--------------------VVGDLG---------------------LTYNTTDTISHLIHN-----SPDLILLIGDVSYANLYLT-NGTSSDCYSCSFPE-TPIHE---------TYQPRWD-------YWGRFMENLTSKVPLMVIEGNHE-------------------IELQAENK--T----------------FEAYSSRFAFPFNESGSSS---------TLYYSF-------------------NAG----GIHFVMLGAYIAYDKS--AEQYEWLKKDLAKVD----RSV---TP--WL---------VASWH-PP-----------WY----SSYT-AHY--------------REA--EC--MKEAMEELLYSY-GTDIVFNGHVHAYERSNRVYNYE-----------LDPCG-----------PVYIVIGDGGNR----EKMAIEHADDPGKCPEPLTTPDPVMGGFCAWNFTPSDKFCWDRQ----PDYSALRESSFGHGILEMKNETWALWTWYRNQD---------------------------------------SSSEVGDQIYIV---------------------------RQPDRC----------------------------------------------PLHHRLVNHC-------------------------------------------------------------------------------------

>AtPAP16

-----------------------------------------------------------------------------------------------------------------MKKPSLFQIIIIVL------------------------------------------------------------------------------------------------------------------------------------------------------------------------------------------------------------------------------------------------------------------------------------------SIPTTTGRTVGNLRVREG-SPFKIA--------------------IFADLH---------FGEDTWTDWGPGQDVNSVNVMSAVLDAE----TPDFVVYLGDVVTAN-------------------------------NIAIQNASLF-------WDKAISPTRDRGIPWATLFGNHDDASFVWPLDWLSSSGIPPLRCPAASDDDGC---------------TFRGTTRVELIQEEIKSSNALSYSMISPKELWPSVSNYVLLVESS---------DHSKPPVALLYFLDSGGGSYPEVISNAQVEWFKTKSNTLN----PYL---RI--PE---------LIFWH-IPSKAYKKVAPRLW--ITKPCVG-SIN--------------KEKVVAQEAENGMMRVLENRS-SVKAVFVGHNHGLDWCCPYKDKL-----------------------WLCFARHTGYGGYG-N-W--------------------------------------------------PR---------GSRILEISEMPFRIKTWIRMED-----G------------------------------------SVHSEVNLT---------YD-------------------------------------------------------------------------------------------------------------------------------------------------------------------

>AtPAP17

---------------------------------------------------------------------------------------------------------MNSGRRSLMSATASLSLLLCIF--------------------------------------------------------------------------------------------------------------------------------------------------------------------------------------------------------------------------------------------------------------TTFVVV-----------------------SNGELQRFIEPAKSD---GSVSFI--------------------VIGDWG-------------------RRGSFNQSLVAYQMGKIGEKI-DLDFVVSTGDNFYDN-----------------------------GLFSEHDPNFEQ-------SFSNIYTAPSLQKQWYSVLGNHD-----------------YRGDAEAQLS--S---------------VLREIDSRWICLRSFVVDAELV-------EMFFV--------------------DTTPFVKEYYTEADGHSYDWRAV--PSRNSYVKALLRDLEVSLKSSK---AR--WK---------IVVGH--------------------HAMR-SIG--------------HHG--DTKELNEELLPILKEN-GVDLYMNGHDHCLQHMSDEDSPI----------------------------QFLTSGAGSKA-W--RGDINPVTIN--------------------------------------PKLLKFYYDGQGFMSARFTHSDAEIVFYDVFGE------------------------------------------ILHK--WVT---------------------------SK--------------------------------------------------QLLHSSV----------------------------------------------------------------------------------------

>AtPAP18

--------------------------------------------------------------------------------------------------------------------MEKWGILLLVT----------------------------LSVSII--------------------------------------------FTSAAADDYVRPKPRETLQFPWKQKSSSVPEQVHIS-------------------LAGDKHMRVTWVTNDKSSP-------------SFVEYGT----SPGKY---SYLGQGESTSY-------------------------------------SYIMYRSGKIHHTVIGPLEADTVYYYRCGGEG----------------------PEFHLKTPPAQ----FPITFA--------------------VAGDLG---------------------QTGWTKSTLDHIDQC-----KYAVHLLPGDLSYAD----------------------------------YMQHKWD-------TFGELVQPLASVRPWMVTQGNHE---KESIP--------------FIVDE-------------------FVSFNSRWKMPYEESGSNS---------NLYYSF-------------------EVA----GVHAIMLGSYTDYDRY--SDQYSWLKADLSKVD----RER---TP--WL---------IVLFH-VP-----------WY----NSNN-AHQ--------------HEG--DE--MMAEMEPLLYAS-GVDIVFTGHVHAYERTKRVNNGK-----------SDPCG-----------PVHITIGDGG-N-R--EGLARKYKDPS-------------------------------------PEWSVFREASFGHGELQMVNSTHALWTWHRNDD-----D----------------------------------EPTRSDEVWLN---------S-----------------LVNSGC---------------------------------LKK--RPQEL---RKMLLEP----------------------------------------------------------------------------------------

>AtPAP19

------------------------------------------------------------------------------------------------------------------MGLNHLTLVCSAI----------------------------ALLSIF------VVS--------QAGVTSTH----------------------VRVSEPSEEMPLET--FPPPACY-NAPEQVHIT-----------------QGDHAGRGMIISWVTPLNEDG------------SNVVTYWI-ANSDGSD----NKSALATTSSY----------------------R---------------YFNYTSGYLYHATIKGLE--TLYNY---------------------------------MSNP-------------------------------------------------------------------------------KGQAVLFAGDLSYAD------------------------------DHPNHDQRKWD-------SYGRFVEPSAAYQPWIWAAGNHE---IDYAE--------------------------------------SIPHKVHLHFGTKSNE-----------------------------------------------LQLTSS---------YSPLTQLMDELKKVN----RSE---TP--WL---------IVLVH-AP-----------WY----NSNN-YHY--------------MEG--ES--MRVTFEPWFVEN-KVDIVFAGHVHAYERSERISNIQYNITDGMSTPVKDQNA-----------PVYITIGDGG-N-I--EGIANNFIDPQ-------------------------------------PSYSAFREASFGHAILEIKNRTHAHYTWHRNKE-----DEFIP------------------------------EAVIADSIWLK---------N-----------------RYYLRE----------------------------------------------EETS-------------------------------------------------------------------------------------------

>AtPAP20

----------------------------------------------------------------------------------------------------------------MVKVLGLVAILLIVL-------------------------------------------------------------------------------AGNVLSYDRQGTRKNLVIHPTNEDDPTFPDQVHIS-------------------LVGPDKMRISWITQSSISP--------------SVVYGT----VSGKY---EGSANGTSSSY------------------------------------HYLLIYRSGQINDVVIGPLKPNTVYYYKC-GGP-------------------SSTQEFSFRTPPSK----FPIKFA--------------------VSGDLG---------------------TSEWSKSTLEHVSKW-----DYDVFILPGDLSYAN----------------------------------MYQPLWD-------TFGRLVQPLASQRPWMVTHGNHE---LEKIP--------------ILHSN--P----------------FTAYNKRWRMPFEESGSSS---------NLYYSF-------------------NVY----GVHIIMLGSYTDFEPG--SEQYQWLENNLKKID----RKT---TP--WV---------VAVVH-AP-----------WY----NSNE-AHQ--------------GEK--ESVEMKESMETLLYKA-RVDLVFAGHVHAYERFSRVYQDK-----------FDKCG-----------PVYINIGDGG-N-L--EGLATKYRDPN-------------------------------------PEISLFREASFGHGQLVVENATHARWEWHRNDD-----D----------------------------------VSVEKDSVWLT---------S-----------------LLADSS---------------------------------CKI---------------------------------------------------------------------------------------------------------

>AtPAP21

-----------------------------------------------------------------------------------------------------------------MKKMKIFGFLISFS----------------------------LFFLSP------FVCQANYDSNFTRPPPRPL-------------------------------------FIVSHGRPKFYPQQVHIS-------------------LAGKDHMRVTYTTDDLNVA-------------SMVEYGK----HPKKY---DKKTAGESTSY-------------------------------------TYFFYNSGKIHHVKIGPLKPNTKYYYRCGGHG----------------------DEFSFKTPPSK----FPIEFA--------------------VAGDLG---------------------QTDWTVRTLDQIRKR-----DFDVFLLPGDLSYAD----------------------------------THQPLWD-------SFGRLLETLASTRPWMVTEGNHE---IESFP--------------TNDHI--S----------------FKSYNARWLMPHAESLSHS---------NLYYSF-------------------DVA----GVHTVMLGSYTPYESH--SDQYHWLQADLRKVD----RKK---TP--WL---------VVVMH-TP-----------WY----STNK-AHY--------------GEG--EK--MRSALESLLYRA-QVDVVFAGHVHTYERFKPIYNKK-----------ADPCG-----------PMYITIGDGG-N-R--EGLALRFKKPQ-------------------------------------SPLSEFRESSFGHGRLRIIDHKRAHWSWHRNND---------------------------------------EMSSIADEVSFE---------SP----------------RTSSHC-------------------------------------------------HSNRYRGEI-----------------------------------------------------------------------------------

>AtPAP22

------------------------------------------------------------------------------------------------------------MKLFGLFLSFTLLFLCPFI--------------------------------------------------------------------------------SQADVPELSRQPPRPIVFVHNDRSKSDPQQVHIS-------------------LAGKDHMRVTFITEDNKVE-------------SVVEYGK----QPGKY---DGKATGECTSY-------------------------------------KYFFYKSGKIHHVKIGPLQANTTYYYRC----------------------GGNGPEFSFKTPPST----FPVEFA--------------------IVGDLG---------------------QTEWTAATLSHINSQ-----DYDVFLLPGDLSYAD----------------------------------THQPLWD-------SFGRLVEPLASKRPWMVTEGNHE---IEFFP--------------IIEHT--T----------------FKSYNARWLMPHTESFSTS---------NLYYSF-------------------DVA----GVHTVMLGSYTDFDCE--SDQYQWLQADLAKVD----RKT---TP--WV---------VVLLH-AP-----------WY----NTNE-AHE--------------GEG--ES--MREAMESLLFNA-RVDVVFSGHVHAYERFKRVYNNK-----------ADPCG-----------PIHITIGDGG-N-R--EGLALSFKKPP-------------------------------------SPLSEFRESSFGHGRLKVMDGKRAHWSWHRNNDSNS---------------------------------------LLADEVWLD---------SL----------------STSSSC-------------------------------------------------WPSSRSNDEL----------------------------------------------------------------------------------

>AtPAP23

--------------------------------------------------------------------------------------------------------------------MTLLIMITLTS----------------------------ISL-LL------AAAETIP-TTLDGPFKPLT------------------RRFEPSLRRGSDDLPMDHPRLRKRNVSSDFPEQIALA-----------------LST-PT-SMWVSWVTGDAIVG-KDVKPLDPSSIASEVWYGK----EKGNY---MLKKKGNATVY----------------------SQLY-------PSD-GLLNYTSGIIHHVLIDGLEPETRYYYRC-GDSSV----------------PAMSEEISFETLPLPSKDAYPHRIA--------------------FVGDLG---------------------LTSNTTTTIDHLMEN-----DPSLVIIVGDLTYANQYRTIGGKGVPCFSCSFPD-APIRE---------TYQPRWD-------AWGRFMEPLTSKVPTMVIEGNHE-------------------IEPQASGI--T----------------FKSYSERFAVPASESGSNS---------NFYYSF-------------------DAG----GVHFVMLGAYVDYNNT--GLQYAWLKEDLSKVD----RAV---TP--WL---------VATMH-PP-----------WY----NSYS-SHY--------------QEF--EC--MRQEMEELLYQY-RVDIVFAGHVHAYERMNRIYNYT-----------LDPCG-----------PVYITIGDGG-N-I--EKVDVDFADD--------------------------------------PGKCHSSYDLFFFNSLNLSN----------------------------------------------------------------------------------------------------------------------------------------------------------------------------------------------------------------------------------------------

>AtPAP24

-------------------------------------------------MARVLGVL--LCLLALFSSSLCLDHAN---GRGDQALAQINVYETSLALDSSVKLHASPQVLGSQGEDTEWVNLAISN-PKPTSDDWIGVFSPAKFDSGNCWPTSGGKEKTP------YICSSPIKYMYCN-SHPDY------------------MKSGNVTLKFQIINQRADVSFALFSNGVQEPHLLGVSNPVAFFNPKAPVYPRL-ALGKNWDEMTVTWTSGYNIDE-----------AVPFIEWSAK--GLPAR-----RSPAGTLTFN----------------------RNSMCG-----NPARGVGWRDPGFFHTSFLKELWPNREYIYRL-GHDLVN-------------GSTIWSKNYTFVSSPYPGQD-SKQRVI--------------------IFGDMG---KGERDGSNEYNDY---QPGSLNTTDQVIKDLK------DIDIVFHIGDLTYSN----------------------------------GYLSQWD-------QFTAQVQPIASTVPYMIASGNHE---RDWPDT------GSFYAGTDSGGE--C----------------GVPAETMFYFPAENRA------------KFWYKT-------------------DYG----MFRFCVADSEHDWREG--TEQYKFIENCLATVD----RKT---QP--WL---------IFIAHRVL-----------GY----STND-WYG--------------KEGTFEEPMGRESLQKLWQKY-KVDLAFYGHVHNYERTCPIYESQ-----CVNNDKDHYSG-------TFKGTIHVVVGGAGSH-L--SPFSSLV-----------------------------------------PKWSLVRDYDFGFVKLTASDHSSLLFEYKKSST-----G------------------------------------QVYDSFNIS---------RDY---------------RDVLAC----------------------------------------------THDSCEPTTSAG-----------------------------------------------------------------------------------

>AtPAP25

---------------------------------------------------------------------------------------------------------------------MRMNKILLVF----------------------------VFLSIA------TVI--------NSGTTSNF----------------------VRTAQPSTEMSLET--FPSPAGH-NAPEQVHIV-----------------QGDYNGRGIIISWVTPLNLAG------------SNVVTYWK-A-VDG-DVKPKKKRGHASTSSY----------------------R---------------FYDYTSGFLHHATIKGLEYDTKYIYEV-GTD-------------------GSVRQFSFTSPPKVGPD-VPYTFG--------------------IIGDLG---------------------QTLASNETLYHYMSNP----KGQAVLFPGDLSYAD------------------------------DHPNHDQRKWD-------SWGRFVEPCAAYQTFIYAAGNHE---IDFVPN-------------IGEPH--A----------------FKPYIHRYHNAYKASKSIS---------PLWYSI-------------------RRA----SAHIIVLSSYSAYGKY--TPQYVWLEQELKKVN----REE---TP--WL---------IVMVH-SP-----------WY----NSNN-YHY--------------MEG--ES--MRAMFESWFVNS-KVDLVLSGHVHSYERSERVSNIKYNITNGLSYPVKDPSA-----------PIYITIGDGG-N-I--EGIANSFTDPQ-------------------------------------PSYSAYREASFGHAVLEIYNRTHAYYTWHRNQD-----N----------------------------------EPVAADSIMLH---------N-----------------RYFFPV----------------------------------------------EELESGNTRA-------------------------------------------------------------------------------------

>AtPAP26

--------------------------------------------------------------------------------------------------------------------MNHLVIISVFL----------------------------SSVLLL------YRG--------ESGITSSF----------------------IRSEWPAVDIPLDHHVFKVPKGY-NAPQQVHIT-----------------QGDYDGKAVIISWVTP-DEPG------------SSQVHYGA----VQGKY---EFVAQGTYHNY----------------------T---------------FYKYKSGFIHHCLVSDLEHDTKYYYKI-ESG-------------------ESSREFWFVTPPHVHPD-ASYKFG--------------------IIGDMG---------------------QTFNSLSTLEHYMES-----GAQAVLFLGDLSYADR-----------------------------YQYNDVGVRWD-------SWGRFVERSTAYQPWLWSAGNHE---VDYMPY-------------MGEVT--P----------------FRNYLQRYTTPYLASKSSS---------PLWYAV-------------------RRA----SAHIIVLSSYSPFVKY--TPQWHWLSEELTRVD----REK---TP--WL---------IVLMH-VP-----------IY----NSNE-AHF--------------MEG--ES--MRAAFEEWFVQH-KVDVIFAGHVHAYERSYRISNVRYNVSSGDRYPVPDKSA-----------PVYITVGDGGNQ----EGLAGRFTEPQ-------------------------------------PDYSAFREASYGHSTLDIKNRTHAIYHWNRNDD---------------------------------GKKVATDEFVLHNQYWGK------------------------NIRRRKLKK----------------------------------------------HYIRSVVGGWIAT----------------------------------------------------------------------------------

>AtPAP27

-------------------------------------------------MAR--NFL--LVLL-WFIVQVSSSHEN---GRGDQALSQIDIYAINLAQHHSAFIHVSPLVLGSQGQDTEWVNVVISN-PEPSSDDWVGVFSPAKFDSSSCAPTD-DKEIAP------FICSAPVKYMYAK-SSPDY------------------MKTGNAVLKFMLINQRADFSFALFTGGLSNPTLVSVSNHVSFINPKAPVYPRL-ALGKKWDEMTVTWTSGYNIGE-----------AVPFVEWSRK--GTRSR-----RSPAGTLTFT----------------------RNSMCG-----APARTVGWRDPGFIHTASLKDLWPNLKYTYRM-GHELMN-------------GSIVWSKNFTFKSSPYPGQD-SLQRVI--------------------IFGDMG---KGERDGSNEYNDY---QPGSLNTTDQLIKDLK------NIDIVFHIGDITYAN----------------------------------GYISQWD-------QFTAQVEPIASTVPYMVASGNHE---RDWPDS------GSFYGGKDSGGE--C----------------GVPAETMFDFPAENKA------------KFWYSA-------------------DYG----MFRFCVADTEHDWREG--SEQYQFIERCLASVD----RRA---QP--WL---------IFIAHRVL-----------GY----STND-WYG--------------QEGSFEEPMGRESLQKLWQKY-KVDIAFYGHVHNYERTCPIYQNQ-----CMDNEKSHYSG-------AFKGTIHVVVGGAGSH-L--SSFSSLK-----------------------------------------PKWSIFRDYDYGFVKLTAFDHSSLLFEYKKSSN-----G------------------------------------AVHDSFTIF---------REY---------------RDVLAC----------------------------------------------VRDSCEPTTLAS-----------------------------------------------------------------------------------

>AtPAP28

----------------------------------------------------------------------------------------------------------MNCSIGNWKHTVLYLTLIVSL--------------------------------------------------------------------------------------LYFIESLISHKLHINYNKIRLKRSPNLP-----------------------------------------------------------------------------------------------------------------------LRFRDDGTFKILQVADMHFGMGMITRC--------------------------------------RDVLDSEFE--------------------YCSDLN-------------------------TTRFLRRMIESE----RPDLIAFTGDNIFGS---------------------------------STTDAAES-------LLEAIGPAIEYGIPWAAVLGNHD--HESTLNRLELMTFLSLMDFSVSQIN--PLVEDETKGDTMRLIDGFGNYRVRVYGAPGSVLANSTVF------DLFFF--------------------DSG----DREIVQGKRTYGWIK---ESQLRWLQDTSIQGH----SQRIHVNP--PA---------LAFFH-IPILEVRE----LWY----TPFI-GQF--------------QEGVACSIVQSGVLQTFVSMG-NVKAAFMGHDHVNDFCGTLKGVW-------------------------------FCYGGGFG-Y--HAYGRPNW----------------------------------------HRRARVIEAKLGKGR-DTWEGIKLIKTWKRLDD-------------------------------------EYLSKIDEQVLWET---------------------------SDSFLK---------------------------------------------------------------------------------------------------------------------------------------------

>AtPAP29

-------------------------------------------------------------------------------------------------------------MADNRRRRSLFDFLLFSV-------------------------------------------------------------------------------------------------FLGLACLCLSPIPATAQ--------------------------------------------------------------------------------------------------------------------RRKLRFSVNGEFKILQVADMHFANGAKTQC--------------------------------------QNVLPSQRA--------------------HCSDLN-------------------------TTIFMSRVIAAE----KPDLIVFTGDNIFGF---------------------------------DVKDALKS-------INAAFAPAIASKIPWVAILGNHD--QESTFTRQQVMNHIVKLPNTLSQVN--P-------PEAAHYIDGFGNYNLQIHGAADSKLQNKSVL------NLYFL--------------------DSG----DYSSVPYMEGYDWIK---TSQQFWFDRTSKRLK----REY---NA--KPNPQEGIAPGLAYFH-IPLPEF-------LSFDSKNATK-GVR--------------QEGTSAASTNSGFFTTLIARG-DVKSVFVGHDHVNDFCGELKGLN-------------------------------LCYGGGFG-Y--HAYGKAGW----------------------------------------ERRARVVVVDLNKKRKGKWGAVKSIKTWKRLDD-------------------------------------KHLSVIDSQVLWNN-------------------------------------------------------------------------------SANKLVVR---------------------------------------------------------------------------------------

>ZmPAP26

--------------------------------------------------------------------------------------------------------------------MRGWGLLVLSL----------------------------HVLSCL------VSG-------VASGRTSSY----------------------VRTEFPSTDIPLESEWFAIPKGY-NAPQQVHIT-----------------QGDYDGKAVIVSWVTP-EEPG------------PSEVFYGK-----EKLY---DQKAEGTTTNY----------------------T---------------FYDYKSGYIHHCLVDGLEYNTKYYYKI-GSG-------------------NSAREFWFETPPAIDPD-ASYTFG--------------------IIGDLG---------------------QTFNSLSTLQHYEKT-----GGQTVLFVGDLSYAD------------------------------RYEHNDGIRWD-------SWGRFVEHSTAYQPWIWNTGNHE---IEYRPD-------------LGETS--V----------------FKPYLHRYMTPYLASKSSS---------PMWYAV-------------------RRA----SAHIIVLSSYSPFVKY--TPQWLWLKNEFKRVD----REK---TP--WL---------IVLMH-SP-----------MY----NSNE-AHY--------------MEG--ES--MRAAFEKWFVKY-KVDLVFAGHVHAYERSYRISNVNYNITSGNRYPVPDKSA-----------PVYITVGDGGNQ----EGLASRFYNPQ-------------------------------------PDYSAFREASYGHSVLQLKNRTHAIYQWNRNDD---------------------------------GNPVPADTVMFHNQYWTS------------------------STRRRRLKK----------------------------------------------NHLHLEDLEDLISLL--------------------------------------------------------------------------------

>ZmPAP10

---------------------------------------------------------------------------------------------------------------MGRHGVDQIGAVAAFV----------------------------WVTLLP------LLLVCVVWPGAQAGHTSEY----------------------RRQLGSAIDMPLDADVFRPPPGY-NAPEQVHIT-----------------QGNHDGTAMIISWVTT-SEPG------------SSTVIYGT----SEDNL---NYTANGKHTQY----------------------T---------------FYNYTSGYIHHCTIKKLEFDTKYYYAV-GIG-------------------QTVRKFWFLTPPKSGPD-VPYTLG--------------------LIGDLG---------------------QSFDSNVTLTHYESNA----KAQAVLFVGDLSYAD------------------------------NYPYHDNVRWD-------TWARFVERSVAYQPWIWTAGNHE---IDFAPE-------------LGETK--P----------------FKPFSHRYPTPYKASGSTA---------PYWYSI-------------------KRA----SAYIIVLASYSAYGKY--TPQYKWLEAEFPKVN----RSE---TP--WL---------VVLMH-AP-----------WY----NSYN-YHY--------------MEG--ET--MRVMYEPWFVKY-KVDVVFAGHVHAYERTHRISNVAYNVVNGLCTPIPDQSA-----------PVYITIGDGG-N-Q--EGLATNMSQPQ-------------------------------------PSYSAFREASFGHAILDIKNRTHAYYTWHRNQD-----G----------------------------------SAVAADSMWFT---------N-----------------RYWEPT----------------------------------------------DDSADDFQ---------------------------------------------------------------------------------------

>ZmPAP30a

-------------------------------------------------------------------------------------------------------------MMGPLPLRRRVGVLLFLA-----------------------------------------LVLLGGGGGADAGTTSSY----------------------RRKLEATVEMPLDADVFRVPPGY-NAPQQVHIT-----------------LGDQEGTAMIVSWVTASEPGN-------------STVAYGE----DPARM---ERRADGAHTRY-------------------------------------DYFNYTSGFIHHCTLRNLKHATKYYYAM-GFG-------------------HTVRTFWFTTPPKPGPD-VPFKFG--------------------LIGDLG---------------------QTFDSNITLSHYESN-----GGDAVLYVGDLSYAD------------------------------NHPLHDNNRWD-------TWARFVERSVAYQPWVWTAGNHE---LDFAPE-------------LGETT--P----------------FKPFAHRYPTPYRAAGSTE---------PFWYSV-------------------KVA----SAHVVVLASYSAYGKY--TPQWAWLQAELARVD----RKT---TP--WL---------VVLTH-SP-----------WY----NSNN-YHY--------------MEG--ET--MRVQFERWLVDA-KVDLVLAGHVHSYERSHRVSNVAYDIVNGKSTPVRSADA-----------PVYVTIGDGG-N-I--EGIADNFTRPQ-------------------------------------PGYSAFREASFGHATLDIKNRTHAYYSWHRNHD-----G----------------------------------AKVVADGVWFT---------N-----------------RYWMPT----------------------------------------------DDDTN------------------------------------------------------------------------------------------

>ZmPAP30b

----------------------------------------------------------------------------------------------------------MSNVLRARRRLDLLQVVLFFV----------------------------AVLLV---------V------VADAGVTSQY----------------------RRKLEATVEMPLDADVFRVPPGY-NAPQQVHIT-----------------LGDQEGTAMIVSWVTA-NELG------------SSTVMYSE-ASPDPEKM---ELRAEGTHTRY----------------------D---------------YFNYTSGFIHHCTLTNLKHSTKYYYAM-GFG-------------------HTVRSFCFTTPPMPGPD-VPFKFG--------------------LIGDLG---------------------QTFDSNTTLSHYEAN-----GGDAVLYVGDLSYAD------------------------------NHPLHDNTRWD-------TWARFVERSAAHQPWVWTAGNHE---LDLAPE-------------LGEHV--P----------------FKPFAHRYPT------------------PFWYSV-------------------RVA----SAHVVVLASYSAYGKY--TAQWEWLRAELARVD----RAA---TP--WL---------IVLVH-SP-----------WY----SSNG-YHY--------------MEG--ET--MRVQFERWIVAA-KADLVVAGHVHAYERSHRVSNVAYDIINARCTPVRTRDA-----------PVYVTVGDGG-N-I--EGIADNFTQPQ-------------------------------------PSYSAFREASFGHATLEIRNRTHAYYAWHRNQD-----G----------------------------------AKVVADGVWLT---------N-----------------RYWMPT----------------------------------------------DDDIN------------------------------------------------------------------------------------------

>ZmPAP18

--------------------------------------------------------------------------------------------------------------MAAPSPLPRFLILLLAV---------------------------------------TSSAFAAAAATGAPVVGEDY------------------VRPPPARCHRKALLSLFPWSKKEESAASSDPQQVHIS-------------------LAGEKHMRITWVTNDNSVP-------------SVVDYGT-------KESTYTMKSQGESTSY-------------------------------------SYLLYSSGKIHHVVIGPLEDNTIYYYRC----------------------GGQGPEFQFKTPPSQ----FPLSLA--------------------VVGDLG---------------------QTSWTTSTLNHIKQC-----EHDMLLLPGDLSYAD----------------------------------YMQHLWD-------SFGTLVEPLASNRPWMVTEGNHE---KEHIPF-------------FESG--------------------FQSYNARWKMPYEESGSRS---------NLYYSF-------------------EVA----GAHIIMLGSYTDYDDS--SDQYAWLKADLAKVD----RKR---TP--WL---------IVLLH-VP-----------WY----NSNW-AHQ--------------GEG--DS--MMASMEPLLYAA-HVDMVIAGHVHAYERAERVYNSR-----------PDPCG-----------AVHITIGDGG-N-R--EGLARRYRNPK-------------------------------------PAWSVFREASFGHGELKIVNSTHAHWTWHRNDD-----E----------------------------------EPVRTDDVWIN---------S-----------------LAGSGC---------------------------------IQEGSRDRES---RKILMSP----------------------------------------------------------------------------------------

>ZmPAP21b

------------------------------------------------------------------------------------------------------------------MRTTRCMLMVAQA----------------------------------------LVVVSALLAAAAAATAAEY-----------------------------VRPPPGRIILTEHTEPAAHPQQVHVS-------------------AVGEKHVRVSWVTDDMRAQ-------------SVVDYGK-------ASRNYTASATGEHTSY-------------------------------------RYFLYSSGKIHHVSIGPLEPSTVYYYRC----------------------GKAGKEFSLRTPPAA----LPIELA--------------------LVGDLG---------------------QTEWTASTLAHASKT-----GHDMLLVPGDLSYAD----------------------------------TQQALWD-------SFGRFVQRHASRRPWMVTQGNHE---VEAPPL----------PVPAGSPP--P----------------FAAYGARWRMPHEESGSPS---------NLYYSF-------------------GAA--GGAVHVVMLGSYAPFNAS--SDQYRWLARDLAAVD----RRA---TP--WL---------VVLLH-AP-----------WY----NTNA-AHQ--------------GEG--EA--MRKAMERLLFQA-RVDVVFAGHVHAYERFARVYDNE-----------ANPCG-----------PVYITIGDGG-N-R--EGLAFNFDKNHTL-----------------------------------APLSMTREASFGHGRLRVVNTTSAHWAWHRNDD---------------------------------------ADSVVRDELWLE---------SLAAKASCR---------QHADPA----------------------------------------------VVDWDDEL---------------------------------------------------------------------------------------

>ZmPAP21c

----------------------------------------------------------------------------------------------------------------MATAALALALLVLAL----------------------------PASLAV----------------------------------------------TSTYVRPPPRATLSSLLEDDAGADGQTPQQVHIS-------------------LVGPDKVRVSWITAADAPA--------------TVDYGT----DPGQY---PFSATGNTTAY-------------------------------------SYVLYQSGSIHDAVIGPLQPSTNYYYRCSG---------------------SSSRELSFRTPPAA----LPFRFV--------------------VVGDLG---------------------QTGWTESTLKHVAAA-----DYDALLLPGDLSYAD----------------------------------LVQPRWD-------SYGRLVEPLASARPWMVTQGNHE---VERLP--------------LLEPR--P----------------FKAYNARWRMPYDYAAADSVAAAPPSDDNLFYSF-------------------DVA--GGAVHVLMLGSYADYAAG--SAQLRWLRADLAALR----RRG---TPPAFV---------LALVH-VP-----------WY----SSNE-AHQ--------------GEG--DA--MRDAMEALLYHGARVDAVFAGHVHAYERFHRVYAGK-----------EDPCG-----------PVYVTIGDGG-N-R--EGLANKFIDPQ-------------------------------------PSISAFREASFGHGRLEVVNATHALWTWHRNDD---------------------------------------NQPVVADQVWIN---------S-----------------LAANPT--------------------------------------------------CNRSIKMM-----------------------------------------------------------------------------------

>ZmPAP21a

--------------------------------------------------------------------------------------------------------MTPSVHADEYVRPPQRPLALMAH-----------------------------------------------------------------------------------------------------DKPASHPQQVHIS-------------------AVGAHHIRITWITDDRSAP-------------SVVDYGT----SPGQY---DASETGYQATY-------------------------------------QFLSYTSGAIHHVTIGPLEPSTTYYYRC----------------------GSAGDEFSFRAPPAT----LPIDFV--------------------VIGDVG---------------------QTEWAASTLSQIGAA-----DHDMMLLPGDLSYAD----------------------------------RQQVLWD-------SWGRLVQPLASARPWMVTEGNHE---KETLRE-------------LGTVR--R----------------FVAYNARWRMPHEESGSRS---------NLYYSF-------------------DAS--GGAVHVVMLGSYADLEEG-WSEQHAWLRRDLAAVD----RRR---TP--WL---------LVLMH-VP-----------WY----NTNR-AHQ--------------GEA--EA--MRRDMESLLYEA-RVDVVFACHTHAYERFARVYDKK-----------ANSQG-----------PMYITIGDAG-N-N--KAEKFMSGHEL-------------------------------------AHLSLFREPSFGYGRLRIIDNRRAVWTWHRNND-----K----------------------------------DAQVSDEVWLE---------S-----------------LATSP----------------------------------------------------------------------------------------------------------------------------------------------

>ZmPAP23

--------------------------------------------------------------------------------------------------MATPTSTVTRGGNRHWHCTQVLPLLLLVP----------------------------LCFALL------VESGGIP-TTLDGPFPPAT------------------RAFDRALRQGSNDVPLTDPRLAPR-VQPPAPEQIALA-----------------ASA-DADSLWVSWVTGRARVGSSNLAPLDPAAAGSEVWYGERSAADAASY---PHVVTGSAEVY----------------------SQLY-------PYP-GLLNYTSGAIHHVRLRGLRPATRYYYRC-GDSSL---------------PGGLSDEHSFTTLPATGAGCYPRRVA--------------------VVGDLG---------------------LTGNSTATVDHLARN-----DPSLVLMVGDMTYANQYLTTGGKGVPCFSCSFPK-APIRE---------SYQPRWD-------GWGRFMEPITSKIPLMVIEGNHE---IEPQG--------------HGGEV--T----------------FASYLARFAVPSKESGSNT---------KFYYSF-------------------NAG----GIHFIMLGAYIDYNRT--GVQYSWLEKDLQRVD----RRA---TP--WV---------VAAWH-PP-----------WY----NSYS-SHY--------------QEF--EC--MRQEMEELLYEY-QVDIVFSGHVHAYERMDRVFNYT-----------LDPCG-----------PIYIGIGDGG-N-I--EKIDMDHADDPGKCPSPSDNHPEFGGL--CHLNFTSGPAKGKFCWDRQPEWSAYRESSFGHGILEVLNSTYALWTWHRNQD-------------------------------------AYAENSVGDQIYIV---------------------------RQPDKC----------------------------------------------LLQPATPLEDALL----------------------------------------------------------------------------------

>ZmPAP13

------------------------------------------------------------------------------------------------------------------MRRGSLSLLLLAA----------------------------VAAVAA------TAVPAEPASTLSGPSRPVT------------------VAIGD--RGHAVDLPDTDPRVQRR-VTGWAPEQIAVA-----------------LSA-SPTSAWVSWITGDYQMG-GAVEPLDPGAVGSVVRYGL----AADAL---DHEATGESLVY----------------------SQLY-------PFE-GLQNYTSGIIHHVRLQGLEPGTRYLYRC-GDPAI---------------PDAMSDVHAFRTMPAVGPGSYPGRIA--------------------VVGDLG---------------------LTYNTTSTVDHLVRN-----RPDLVLLLGDVCYANLYLT-NGTGADCYSCAFAKSTPIHE---------TYQPRWD-------YWGRYMEPVTSSIPMMVVEGNHE---------------------IEQQIHNRT----------------FAAYSSRFAFPSEESGSSS---------PFYYSF-------------------DAG----GIHFVMLASYADYSRS--GAQYKWLEADLEKVD----RSV---TP--WL---------IAGWH-AP-----------WY----TTYK-AHY--------------REA--EC--MRVEMEELLYAY-GVDVVFTGHVHAYERSNRVFNYT-----------LDACG-----------PVHISVGDGGNR----EKMATAHADEAGHCPDPASTPDPFMGGRLCAANFTSGPAAGRFCWDRQPEYSAYRESSFGHGVLEVRNDTHALWRWHRNQD----------------------------------LHAAAAANVAADEVYIV---------------------------REPDKC----------------------------------------------LAKTARLLAY-------------------------------------------------------------------------------------

>ZmPAP21d

------------------------------------------------------------------------------------------------------------------MATAALALLVLAL----------------------------PASLAV----------------TSTYVRPPP--------------------------------RATLSLLQDAGSDGQTPQQVHIS-------------------LVGPDKVRVSWITAADAPA--------------TVDYGT----ASGQY---PFSATGNTTSY-------------------------------------SYVLYHSGSIHDAVIGPLQPSTTYYYRCSG---------------------SASRDLSFRTPPAV----LPFRFV--------------------VVGDLG---------------------QTGWTESTLKHVAAA-----DYDALLLPGDLSYAD----------------------------------FVQPRWD-------SYGRLVEPLASARPWMVTQGNHE---VERLP--------------LLEPR--P----------------FKAYNARWRMPYDYAADGTPPSDD----NLFYSF-------------------DVA--GGAVHVLMLGSYADYAAG--SAQLRWLRADLAALR----RRG---TPPAFV---------LALVH-AP-----------WY----SSNK-VHQ--------------GEG--DA--MRDAMEALLYHG------------ASTRCSRATSTR-------------------------------TSGSTAST-P--ARRIPAA-----------------------------------------PSTSPSATAATGRG----------------------------------------------------------------------------------------------------------------------------------------------------------------------------------------------------------------------------------------------------

>ZmPAP2d

-------------------------------------------MGLSGSGAVASIVFLGLCATVSCWPAPPPPEMLHESFAGKSEFRTVNRRRLSSCSNPSPYLSINVSSGGAPLPDEAFLTVTVAGVLRPDADDWVAMITPCSSSVSGCPLSGVNYVQTGDLAHLPLLCHYPVKAQYMK-RDPGYLGCKTAACQKRDASGACSVRTCAATVTFHVINFRTDVEFVLFSGGFRTPCVLKRSGALRFANPASPLYGHLSSTDSTATSMRLTWVSGDGRPQ--------------QVQYGG------------GKSATSQVATF---------------------TRNDMCSSPLLPSPAKDFGWHDPGYIHTAVMTGLQPSQSYTYRY-GSD-----------------SVGWSDTNTFRMPPAAGSD--ETSFV--------------------IYGDMG---KAPLDPSVEHYI----QPGSISVVKAVAKEIQTG----KVNSVFHIGDISYAT----------------------------------GFLVEWD-------FFLNLIAPLASRVPYMTAIGNHE---RDYAES------GSVYVTPDSGGE--C----------------GVAYESYFRMPAVSKD------------KPWYSI-------------------EQG----SVHFVVMSTEHKWSEM--SEQYKWMNQDLSSVN----RSR---TP--WV---------IFIGH-RP-----------MY----SSHV-GIP--------------VNV--DL-AFVASVEPLLLKH-QVDLVFFGHVHNYERTCAIYKNI-----CKGKPKKDESGIDTYDNSKYTAPVHATVGAGGFS-L--DKFPRIVL----------------------------------------NKWSLSRVSEFGYARVHATRGDMLVQFVSSSTM------------------------------------------EVLDQFRFV----------------------------KPDPA----------------------------------------------RRLRNKPV---------------------------------------------------------------------------------------

>ZmPAP2b

--------------------------------MMAFFVVQRGSSQLTPLLALALLGLLRCAAVSCSAAAAATTLLHRESYAGKSEFRTVNRKPLGSCVDPSPYLAIDVGAAGPIPDEAFLQVTVSGV-QRPDPSDWVAMITPSNSSVAGCPLSEVNYVETGDLANLPLLCHYPVKAQYLT-SDPGYLGCKNAGCGKRDASGACTARTCAATLTFHVVNFRTDVEFVLFSGGFKAPCLLKRSGARRFANPASPLYGHLSSTDSKATSMRLTWVSGDGNPQ--------------RVQYGD------------GKSSTSEVATF---------------------TQDDMCSISVLPSPAKDFGWHDPGYIHSAVMTGLQPSQSYTYRY-GSD-----------------SVGWSDTVKFRTAPAAGSD--ELSFV--------------------IYGDMG---KAPLDPSVEHYI----QPGSVSVAKAVAKEIQTG----NVDSIFHIGDISYAT----------------------------------GFLVEWD-------FFLHLITPLASQVPYMTAIGNHE---RDYASS------ASVYVTPDSGGE--C----------------GVAYESYFPMPAVSKD------------KPWYSI-------------------EQG----TVHFIVMSTEHEWSEK--SEQYNWMDEDLSSVD----RSR---TP--WV---------IFIGH-RP-----------MY----SSHG-GIL--------------PNV--DS-NFVASVEPLLLNY-QVDLVFFGHVHNYERTCAVYQGN-----CKGMPTTDKSGIDVYDNSNYTAPVHVIVGAGGFS-L--DSFPNKG-----------------------------------------EAWSLSRVSEFGYGKVHATRTDMLVQFVNSSSM------------------------------------------EVRDQFRIV---------------------------KGAPAN----------------------------------------------KSLSLIIQ---------------------------------------------------------------------------------------

>ZmPAP24a

-------------------------------------------------MRPAEGAARLVCLLLLAAV--------AAGHAGVQPLARIAIHRARFALDASAAVRASPELLGTKGEDTAWVKVDLVT-PHPSADDWVGVFSPSKFNASTCLGSH-GSGPGP------VICSAPIKYQFANYSS-GY------------------GESGKGALQFQLINQRQDFSFALFTGGLSNPKLIAVSNAIAFANPKAPVYPRL-AQGKSWNEMTVTWTSGYESDE-----------AYPFVEWGMKWSPPV-------RSAAGTVTFD----------------------RESVCG-----EPARSVGWRDPGFIHTAFLTDLWPNKEYYYKI-GHMLPD-------------GSVVWGKLSSFKAPPFPGQK-SLQRVV--------------------IFGDMG---KAERDGSNEYSNY---QPGSLNTTDTLVKDLD------NIDMVFHIGDITYAN----------------------------------GYISQWD-------QFTQQVEEITSRVPYMVASGNHE---RDWPNS------GSFFNGTDSGGE--C----------------GVVAETMYYTPTENRA------------NYWYSA-------------------DYG----MFRFCVADSEHDWREG--TEQYEFIESCLATVD----RKK---QP--WL---------VFIAHRVL-----------GY----SSGF-FYG--------------VDGSFAEPMSRQSLQKLWQKY-RVDLAFYGHVHNYERTCPVYEEQ-----CMSSEKSHYSG-------TMNGTIHVVVGGGGSH-L--SNFTAQV-----------------------------------------PPWSVYREMDYGFVKLTAFNYSSLLYEYKRSSD-----G------------------------------------QVYDSFTMH---------REY---------------RDLLAC----------------------------------------------VKDSCPPTSPAT-----------------------------------------------------------------------------------

>ZmPAP2c

------------------------------------------------------------------------------------------------------------------MKGSSCNIFLYDA---------------------------------------------------------------------------------------------------------------------------------------------------------------------------------------------------------------------------------------------------TGMLCQRGDSV-----------------------------GWSDTVKFRTAPAAGSD--ELSFV--------------------IYGDMG---KAPLGPSVEHYI----QPGSVSVAKAVAKEIQTG----NVDSIFHIGDISYAT----------------------------------GFLVEWD-------FFLHLITPLASQVPYMTAIGNHE---RDYVNS------ASVYVTPDSGGE--C----------------GVAYESYFPMPAVSKD------------KPWYSI-------------------EQG----TVHFIVMSTEHEWSEK--SEQYNWMDEDLSSVD----RSR---TP--WV---------IFIGH-RP-----------MY----SSYG-VIL--------------PNV--DS-NFVASVEPLLLNY-QVDLVFFGHVHNYERTCAVYQGN-----CKGMPTSDKSGIDVYDNNNYTAPVHVIVGVGGFS-L--DNFPNKG-----------------------------------------EAWSLSRISEFGYGKVHATRTDMLVQFVNSSSM------------------------------------------EIRDQFRIV---------------------------KGAPAN----------------------------------------------KSLSLII----------------------------------------------------------------------------------------

>ZmPAP1c

------------------------------------------------MRAPLLL----WAAATWLAVSAVAHPGRRGGGGGEQPLSRIAVESAVLAVDDAAHVRASPLVLGLKGESSEWVDVEFFH-PDPSSDDWIGVFSPADFSAAICEPEN-PRQSPP------VLCSAPIKYQFATFKNDGY------------------NKTGKGYLKLQLINQRGDFSFALFSGGLLKPKLIAVSNKVAFANPKAPVYPRL-AQGKSWNEMTVTWTSGYDITE-----------AVPFVEWGEK--GGRRF-----LAPAGTLTFD----------------------RNSMCG-----APARTVGWRHPGYIHTSYLKDLWPDSRYTYRL-GHRLMN-------------GTRVWSKSYSFRASPYPGQD-SLQRVV--------------------IFGDMG---KAEADGSNEFNNF---QPGSLNTTYQITSDIE------NIDMVVHIGDICYAN----------------------------------GYLSQWD-------QFTAQIEPIASTVPYMVGSGNHE---RDWPGT------GSFYGNLDSGGE--C----------------GVPAQTVFYTPAENRA------------KFWYAT-------------------DYG----MFRFCVAHTEEDWRPG--TEQYRFIERCLSSVD----RQK---QP--WL---------VFLAHRVL-----------GY----SSCA-YYE--------------SEGTFEEPMGREALQELWQKY-KVDLAFYGHVHSYERTCPVYQSQ-----CVVDASDHYSG-------PFQATTHVVVGGAGAS-L--SEFAASK-----------------------------------------IQWSHFTDFDHGFVKLTAFNHSSLLFEYKKSRD-----G------------------------------------NVYDRFTIS---------RDY---------------RDVLAC----------------------------------------------SVDNCPRTTLAS-----------------------------------------------------------------------------------

>ZmPAP1b

------------------------------------------------MKGRLLLLLWAWAAATWVSVSAVTRLGR--GVAGEQPLSRIAVERVVLAVNDAAYVKASPLVLGHKGENSEWADVEFFH-PNPSDDDWIGVFSPANFSDAICEAEN---TGTP------VLCTAPIKYQFANFENDGY------------------NKTGKGYLKLQLINQREDFSFALFSGGLSKPKLISVSNKVAFANPKAPVYPRL-AQGKSWNEMTVTWTSGYDITE-----------AVPFVEWGEK--GGRRL-----LAPAGTLTFD----------------------RNSMCG-----SPARTVGWRHPGYIHTSFLKDLWPDSPYTYRL-GHRLMN-------------GTRVWSKSYSFKASPYPGQD-SLQRVV--------------------VFGDMG---KAEADGSNEFSDF---QPGSLNTTYQIIRDLE------DIDMVVHIGDICYAD----------------------------------GYLSQWD-------QFTAQIEPIASRVPYMIGLGNHE---RDWPGT------GSFYGNLDSGGE--C----------------GVPAQTVFYTPAENRA------------KFWYAT-------------------DYG----MFRFCIANTEEDWRPG--TEQYKFIEQCLSSVD----RQK---QP--WL---------IFLAHRVL-----------GY----SSCT-YYE--------------SEGTFEEPMGREALQELWQKY-KVDIAFYGHVHSYERTCPVYQSQ-----CVVDGSDHYSG-------PFQATTHVVVGGAGAS-ILDSEFTTSK-----------------------------------------IQWSHFTDFDHGFVKLTALNHSSLLFEYKKSRD-----G------------------------------------NVYDHFTIS---------RDY---------------RDILAC----------------------------------------------SIDNCPRSTLAS-----------------------------------------------------------------------------------

>ZmPAP2a

---------------------------------------------------------------------------------MYPE-NPHLRFLLFLAVAAVAAGGAAANTTLTASLSGNQIKIIWSGLPAPDGLDYVAIYSPPSSRDRDFLGYLFLNGSASWRGGSGELSLPLLPTLRAPYQFRLF--------RWPAKEYSYHHVDHDQNPLPHGKHRVAVSADVSVGDPA-RPEQLHLA-----------------FAD-EVDEMRVLFVCGDRGER--------------VVRYGL----QKEDDKEWKEVGTDVSTYE----------------------QRHMCDW----PANSSVAWRDPGFVFDGLMKGLEPGRRYFYKV-GSD-----------------TGGWSEIYSFISRDSEASETNAF-----------------------LFGDMG--------TYVPYNTYIRTQSESLSTVKWILRDIEALGD--KPAFISHIGDISYAR----------------------------------GYSWVWD-------HFFSQIEPIAANTPYHVCIGNHE---YDWPSQPWKPWWATY--GTDGGGE--C----------------GIPYSVKFRMPGNSILPTGNGGPDTR--NLYYSF-------------------DSG----VVHFVYMSTETNFVQG--SDQHNFLKTDLEKVN----RSR---TP--FV---------VFQGH-RP-----------MY----TSSD-ETR--------------DAA--LKQQMLQNLEPLLVTY-NVTLALWGHVHRYERFCPMKNSQ-----CVNTSSSFQYS---------GAPVHLVIGMGGQD-W--QPVWQPRPDHPDVPIFPQ------------------------------PERSMYRGGEFGYARL-VATREKLTLTYVGNHD-----G------------------------------------QVHDMVEIFSGLVSPSNSSVAEAVDGTKLGTGVSTVRKISPL-------------------YLEIGGSVMFALLLGFSFGILVRRKKEAAQWTQVKNEES-----------------------------------------------------------------------------------

>ZmPAP1a

MVALAQFARQVRPSPICNLDVTLWFHNLLARFYSTKMVYRKREKSVCWSQPSRMLSLPRTAAAVTVYALVALIAGAAAAGGGEQPLSRIAIHRATVAPQPGAFVDASPALLGREGEDREWVTVTYSN-PRPSKDDWIGVFSPANFNDSICPPENEWVEPPL-------LCTAPIKFQFANYTNRDY------------------GNTGKGSLRLQLINQREGFSFALFSGGLSNPKLIAHSKSVTFINPKTPVYPRL-AQGKSWNEITVTWTSGYGTNE-----------ATPFVRWGI-------EGQIQTLSPAGTLTFS----------------------RDTMCG-----PPARTVGWRDPGFIHTSFLKDLWPNLLYTYQV-GHRIFN-------------GSIVWGHQYSFKAPPYPGED-SLQRVV--------------------ILGDMG---KAEVDGSNEFNDF---EPGSLNTTNQLIKDLK------NIDVVFHIGDITYAN----------------------------------GYLSQWD-------QFTAQVEPIASTVPYMVGSGNHE---RDWPGS------GSFYGNLDSGGE--C----------------GVPAQNMFYVPAENRE------------QFWYST-------------------DYG----MFRFCVANTELDWRPG--TEQYRFIEHCLSSVD----RQK---QP--WL---------IFLAHRVL-----------GY----SSAT-FYA--------------DEGTTEEPMGRESLQPLWQKY-KVDIAMYGHVHGYERTCPVYENA-----CVAKGSDLYAG-------AFTATTHVVVGGGGAS-L--ADYTAAR-----------------------------------------ARWSHVRDRDFGFVKLTAFNHTRLLLEYKKSRD-----G------------------------------------SVHDHFTIS---------RDY---------------RDVLAC----------------------------------------------GVDNCPSTTLAS-----------------------------------------------------------------------------------

>ZmPAP24b

----------------------------------------------MGREAPAMAVALLAALVASAAMFMLAGTASASPAEGIQPLSKIAVHRATVEMQPSAYVRATPSLLGEQGEDTEWVTVKFGW-KEPSEDDWIGVFSPSEFNSSATCPNP-WPAEEP------YLCTAPIKYQFANYSA-NY------------------IYWGKGSIRLQLINQRSDFSFALFTGGLSNPRLIAVSEPISFKNPKAPVFPRL-AQGTSHDEMTVTWTSGYAIDE-----------AYPFVEWGALVAGGVRHT---ARAPAGTLTFN----------------------RGSMCG-----EPARTVGWRDPGFIHTAFLRDLWPNKEYHYRI-GHELPD-------------GSVVWGKPYSFRAPPSPGQP-SLQRVI--------------------VFGDMG---KAERDGSNEYAAY---QPGSLNTTDALIADLD------NYDIVFHIGDMPYAN----------------------------------GYISQWD-------QFTAQVAPITARKPYMVGSGNHE---RDWPDT------AAFWDVMDSGGE--C----------------GVPAETYYYYPAENRA------------NFWYKV-------------------DYG----MFRFCVGDSEHDWRVG--TPQYDFIEHCLSTVD----RKH---QP--WL---------IFATHRVL-----------GY----SSNA-WYA--------------GEGSFEEPEGRENLQRLWQKY-RVDIAFFGHVHNYERTCPMYQSQ-----CMTSEKTHYSG-------TMNGTIFVVAGGGGCH-L--SSYTTAI-----------------------------------------PKWSIYRDYDFGFVKLTAFNHSSLLFEYKKSSD-----S------------------------------------KVYDSFTID---------RDY---------------RDVLRC----------------------------------------------VHDSCFPTTLAT-----------------------------------------------------------------------------------

>ZmPAP2e

----------------------------------------------------------------------------------------------------------------------------------------------------------------------------------------------------------------------------------------MEPDPVAAG-------------------------------------------------------------------------------------------------------------------------------------------------------------------------ASSSPARLALHLIYSSSFFPAGFL-------------------------------------------------LCEKKQVTQLAWCA----SSPQMTWR-------------------------------------------LVEWD-------FFLNLIAPVASRVPYMTAIGNHE---RDYVES------GSVYVTPDLGGE--C----------------GVAYESYFCMPAISKD------------KPWYSI-------------------EQG----SVHFVVMSTEHKWSEM--SEQYKWMNQDLSSVN----RSR---TP--WI---------IFIGH-RP-----------MY----SSHV-GIP--------------VNV--DL-TFVASVEPLLLKH-QVDLVFFGHVHNYERTCVVYKNR-----CKGKPKKDASGIDTYDNNKYTAPVHATVRAGGFS-L--DKFPRIVL----------------------------------------NKWSLSRVSEFGYARVHATRGDMLVQFKKPGDDMNGDMASLSTLLANRKKGHFMKGDAVIVIKGDLKNLEGWVEKVEDETVHIR---------PKISDLPKTLAFNEKELCKYFKPG------DHVKVILGVQEGATGMVVKVEGHVLIILSDTTKEHVLCSSMEAWTNSSMWSFRSSRKLSVLAGDFLLSRACVALAALGNTEVVSLMATAVEHLVTGETMQISTSRNKRVFKRTVSGLVGALFNIILHLQSPYYTA

>ZmPAP2f

----------------------------------------------------------------------------------------------------------------------------------------------------------------------------------------------------------------------------------------MEPDLVAAG-------------------------------------------------------------------------------------------------------------------------------------------------------------------------ASSSPARLALHLIYSSSFFPAGFL-------------------------------------------------LCEKKQVTQLAWCA----SSPQTTWR-------------------------------------------LVEWD-------FFLNLIAPVASRVPYMTTIGNHE---RDYAET------GSVYVTPDSGGE--C----------------EVAYESYFCMPAVSKD------------KPWYSI-------------------EQG----SVHFVVMSTEHKWSEM--SEQYKWMNQDLSSVN----RSR---TP--WI---------IFIGH-RP-----------MY----SSHV-GIP--------------VNV--DL-TFVASVEPLLLKH-QVDLVFFGHVHNYERTCAVYKNR-----CKGKPKKDASGIDTYDKCKYTAPVHATVRAGGFS-L--DKFPRIVL----------------------------------------NKWSLSRVSEFGYARVHATRGDMLVQFVSSRTM------------------------------------------EVLDQFRIV---------------------------KPDPAR----------------------------------------------RLRNKPV----------------------------------------------------------------------------------------

>ZmPAP7d

----------------------------------------------------------------------------------------------------------------MANRSVAMGLALAFV-------------------------------------------------------------------------------------------------------------------------------------------------------------------------------------------------------------------------------------------------------------AMTALCC---------------------APGAAELPRLDHPARSD---GSLKLL--------------------VVGDWG-------------------RKGTHNQSRVADQMGRVGEKL-DIDFVISTGDNFYKN-----------------------------GLKGVRDQAFEE-------SFVDIYTAQSLQKPWYSVLGNHD-----------------YRGNALAQLS--P---------------VLRKIDDRFICMRSFIVNAELV-------DFFFV--------------------DTTPFQLEYWTHPGKHRYDWRGV--APRGNYLANLLKDLDVAMKKST---AR--WK---------IVVGH--------------------HTMR-SVS--------------EHR--DTEELLELLLPVLKDN-GVDFYINGHDHCLEHISSRDSPL----------------------------QYFTSGGGSKA-W--RGVFHPN-----------------------------------------KDKLRFFYDGQGFMSLQLNQDQAHFIFYDVFGN------------------------------------------ILYR--WSS---------------------------RHPQSS----------------------------------------------TYLDEE-----------------------------------------------------------------------------------------

>ZmPAP7c

-----------------------------------------------------------------------------------------------------------------MAQGSRSAAVPAAA----------------------------------------------------------------------------------------------------------------------------------------------------------------------------------------------------------------------------------------------------SFFALLAVAAPLLLCC---------------------APAAAELARLEHPPKDG---GSLSLL--------------------VVGDWG-------------------RKGTFNQSRVAHQMGRVGEQL-SIDFVISTGDNFYEN-----------------------------GLTGTDDEAFEQ-------SFTDIYTAKSLQKPWYLVLGNHD-----------------YRGDALAQLS--P---------------VLRKIDSRFICIKSFVVNAEIV-------EFFFV--------------------DTTPFQLKYWTHPKDDHYDWRGV--APRENYINNLLKDLDGAMKTST---AA--WK---------VAVGH--------------------HTMR-SVS--------------DHG--DTKELLQLLLPVLQAN-GVDFYINGHDHCLEHISSRDSPI----------------------------QYFTSGGGSKA-W--RGVQNPT-----------------------------------------EDDLKFFYDGQGFMSLQLDRSQAKFTFYDVDGK------------------------------------------ALYSYTRSS----------------------LMETGHHLQAS----------------------------------------------GYVNEE-----------------------------------------------------------------------------------------

>ZmPAP257e

-----------------------------------------------------------------------------------------MGNSLVTATYCDLNPMAMQVVQGKWPPPVSYTVIVARL------------------------------------------TQHALAKSQPAREQPPY-----------------------QAGKRNCVKEIPHLLTKEYITCLALICLVYLLPVVPYQHFSQELKQKHNDRTENEVAESNDWMSPGYANA----------------GSSPVPTPPSGKGLKASTKPKATKGQK---------------------------------SGPQTPLGFGSPGNPSTPVGGCRYDSSLGYCC-----------------------LLGLLTKFLNLLKGAPG-GIVDLN------------NAAETLELITAEIG---------------VDDSRHGEVSDDMSILQMGRVGEKL-DIDFVISTGDNFYKN-----------------------------GLKGVHDQAFKE-------SFMDIYTAQSLQKPWYSVLGNHD-----------------YRGNALAQLS--P---------------VLRKIDDRFICMRSFIVNAELV-------DFFFI--------------------DTTPFQLEYWTHPGKHRYDWRGV--APRGKYIANLLKDMDVAMKRST---AR--WK---------IVVGH--------------------HTMR-SVS--------------EHG--DTEELLELLLPVLKDN-GVDFYINGHDHYLEHISSRDSPL----------------------------QYFTSGGGSKA-W--RGVFHPN-----------------------------------------KDKLRFFYDGQGFMSLQLNQDQAHFIFYDVFGN------------------------------------------ILYR--WSS---------------------------RHPQSS----------------------------------------------TYLDEE-----------------------------------------------------------------------------------------

>ZmPAP7a

-----------------------------------------------------------------------------------------------------------------MARVASLLLTVIVA-------------------------------------------------------------------------------------------------------------------------------------------------------------------------------------------------------------------------------------------------AAASLLLLLAPSAAELARV-------------------------------EHPPKND---GSLAIL--------------------AVGDWG-------------------RRGQFNQTLVAQQMGVVGEKL-DIDFVISTGDNIYDD-----------------------------GIANTSDPLFKE-------CFTNIYTAQSLQTPWYIVLGNHD-----------------YTGNALAQQD--P---------------AIREVDSRYLNLAKSFIVNSGIA------DFFLL--------------------DTSPFYLKYW---NSSKYDWRDV--APRDTYIQNLLDDLDDALVQSE---AP--WK---------IVVGH--------------------HPIS-SGC--------------EHG--NTTELQELLRPILEAR-GVDMYVNGHDHCLQHISSRNSPV----------------------------QFMTSGGGSKA-W--AGKFKTT-----------------------------------------SDKIEFIYDGQGFMSMQLSSTEAHLVFYDVAGN------------------------------------------VLHS--YDS---------------------------TKNEEE----------------------------------------------DDGWPAGQQAAVRGSIDRGNL--------------------------------------------------------------------------

>ZmPAP7f

----------------------------------------------------------------------------------MHGVTLIP-------------GVPEKDYARARYRYQNLSKLLPLF----------------------------LTEQSL------HEAKADPAKATTNGVPNGT------------------------GHSYSGTVPSQQNSALTPDGNYRAPLLGGVPSAGYLDT----------TYGYDSTWAQFAWYDGSAYAN------------------GQQRTTTTNRFPSSSFSGNGSSARYQNKSSTTQQMLHSCFCPLCIVFKQAPTYPNRVYPSPRPYTQYENSVKTGLPYGSNGYDSRIYGRC-GLGMDNRYRPRACSGYYGYGNESQDGTIELNRGPRSGRFKNQKLYGHTVTIDPVMVQKRRIYDITNVLEGIG-LIEKKLKNNIRWKGVDDSRPGEFSDDMSILQMGRVGEKL-DIDFVISTGDNFYKN-----------------------------GLKGVRDQAFKE-------LFMDIYIAQSLQKPWYS------------------------------ELV-----------------------------------------------DFFFV--------------------DTTPFQLEYWTHPGKHRYDWRGV--APRGKYIANLLKDMDVAMKRST---AR--WK---------IVVGH--------------------HTMR-SVS--------------EHG--DTEELLELLLPVLKDN-GVDFYINGHDHCLEHISSRDSPL----------------------------QYFTSGGGSKA-W--RGVFHPN-----------------------------------------KDKLRFFYDGQGFMSLQLNQDQAHFIFYDVFGN------------------------------------------ILYR--WSS---------------------------RHPQSS----------------------------------------------TYLNEE-----------------------------------------------------------------------------------------

>ZmPAP7g

--------------------------------------------------------------------------MNRKCLYSRLGTTTLSLRTMKAQISNGRGGCLALKLAEHEGRGGNLSKLLPLF----------------------------LTEQSL------HEAKADPAKATANGVPNGT------------------------GHSYSGTVPSQQNSALTPDGNYRAPLLGGVPSAGYLDT----------TYGYDSTGAQFAWYDGSAYAN------------------GQQRTTTTNRFPSSSFSGNGSSARYQNKSSTTQQMGMQNRRPTTT--SAAPTYPNRMYPSPRPYTQYGNSVKTGLPYGSNGYDSRINGRC-GLGMDNRYRPRACSGYYGYGNESQDGTIELNRGPRSGRFKNQKLYGHTVTIDPVMVQKRRIYDITNVLEGIG-LIEKKLKNNIRWKGVDDSRPGEFSDDMSILQMGRVREKL-DIDFVISTGDNFYKN-----------------------------GLKGVRDQAFKE-------SFMDIYIAQSLQKPWYS------------------------------ELV-----------------------------------------------DFFFV--------------------DTTPFQLEYWTHPGKHRYDWRGV--APRGKYIANLLKDMDVAMKRST---AR--WK---------IVVGH--------------------HTMR-SVS--------------EHG--DTEELLELLLPVLKDN-GVDFYINGHDHCLEHISSRDSPL----------------------------QYLTSRGGSKA-W--RGVFHPN-----------------------------------------KDKLRFFYDGQGFMSLQLNQDQTHFIFYDVFGN------------------------------------------ILYR--WSS---------------------------RHPQSS----------------------------------------------TYLDEE-----------------------------------------------------------------------------------------

>ZmPAP7b

------------------------------------------------------------------------------------------------------------------------------------------------------------------------------------------------------------------------------------------------------------------------------------------------------------------------------------------------------------------------------------------------------------------------------------------------------------------------------------------------------------MGVVGQKL-DIDFVISTGDNIYDD-----------------------------GIANTSDPLFKE-------SFSNIYTANSLQKPWYLVLGNHD-----------------YTGNALAQLD--P---------------AIRKVDSRYTAIAKSFIVNSGIA------DFFLV--------------------DTTPFIVHYW---NNTKFDWRGV--APRDTYIANLLKDLKCALTASK---AP--WK---------IVVGH--------------------HPIS-SAC--------------GHG--NNTELEELLLPVLTTH-GVDMYLNGHDHCLQRVSSRDSRL----------------------------QLLTSGGGSKA-W--AGKFKPT-----------------------------------------LDKVEFLYDGQGFMSMRLSRTEARLAFFDVAGS------------------------------------------VLHC--WTM---------------------------HAQKLT----------------------------------------------TTTTAAAARH-------------------------------------------------------------------------------------

>ZmPAP28a

-----------------------------------------------------------------------------------------------------------MEVAERPMASSHLAALVSLL----------------------------------------------------------------------------------------------------------IPCLLALL------------------------------------------------------------------------------------------------------------------------------LLRLAAVLDPDPDA-----------------------------AVPRIKAAAPLPLRFRHDGAFKIL--------------------QVADMHFGNGATTRCRDVGPEGGGARCSDLNTTRFLRRVIEAE----KPDLIAFTGDNIFGG---------------------------------SATDAAES-------LLRAISPAIEYRVPWAAILGNHD--QESTMTREELMTFMSLMDYSVSQVN--P---------PGFLVHGFGNYHVGIHGPFGSELVNTSLL------NLYFL--------------------DSG----DREMVNGVKTYGWIR---ESQLAWLRSTSLELQ----KKI---HA--PA---------LAFFH-IPIPEVRG----LWY----SGFK-GQY--------------QEGVACSSVHSGVLGTLVSMG-DVKSVFLGHDHLNDFCGNLNGIW-------------------------------FCYGGGFG-Y--HAYGRPHW----------------------------------------PRRARIIYSELKKGQ-RSWLEVDSISTWKLLDD-------------------------------------EKLSKIDEQVIWRH-------------------------------------------------------------------------------STDDSDHRVYL------------------------------------------------------------------------------------

>ZmPAP14

---------------------------------------------------------------------------------------------------------MGGCGLGRGRRTACLLLLLPPL----------------------------------------------------------------------------------------------------------------------------------------------------------------------------------------------------------------------------------------------------LLFAVADAAASGKEKQ-----------------------GVSGKLRFRRES------GTFKVV--------------------QVADMH-YADGRSTACEDVLPSQVAGCTDLNTTAFLYRVFRAE----DPDLVVFTGDNIYGA---------------------------------DSTDAAKS-------MDAAIAPAIDMKLPWAAVIGNHD--QEGTLSREGVMRHLVGMKNTLASFN--P---------EGIEIDGYGNYNLEVSGVEGTSMDEKSVL------NLYFL--------------------DSG----DYSTVPSINGYGWIK---ASQQVWFQQTSSSLQ----AKYMNKNPKQKE-----PAPGLVFFH-IPLPEFSS----FTA----SNFT-GVK--------------QEGISSASINSGFFASMVEAG-DVRAAFVGHDHINDFCGKLSGIQ-------------------------------LCYAGGFG-Y--HAYGKAGW----------------------------------------SRRARVLSVQLEKTDSGEWRGVKSIKTWKRLDD-------------------------------------KHLSTIDSEVLWNR-----------------------------GSNG----------------------------------------------RRGKNPDGS--------------------------------------------------------------------------------------

>ZmPAP16

--------------------------------------------------------------------------------------------------------------MHKWWRSPGPALLAAAL---------------------------------------------------------------------------------------------------------------------------------------------------------------------------------------------------------------------------------------------------AIVVAALRPATEHAAAK------------------------------GRPPLRFGPG-GAFKVA--------------------LFADLH---------YGEDAWTDWGPAQDAASDRVMAAVLDAE----NPDLVVYLGDLVTAN-------------------------------NLPVPNASLY-------WDRAVSAARGRGVPWATVFGNHDDMAFEWPPEWFSPDGVPPLRWPPGPGSG-C---------------GFRGTPRTDLMAAETGANRLLSYSSSGPRELWPGVSNYVLQVLSRGRRARGDGHDHDPALLMYFLDSGGGSYTEVVS--SAQVRWFHTQSQFLN----PDG---RI--PE---------LIFWH-IPSTAYAKVAPKAKSEIRKPCVG-SIN--------------EEEVAPQAAEWGMMDALAKRS-SVKAVFVGHNHGLDWCCPYDGEE------------------REQELWLCFARHTGYGGYG-D-W--------------------------------------------------PR---------GARILEVTEEPFSAVSWIRMEN----------------------------------------GTRHSDVTLTS------------------------------------------------------------------------------------------------------------------------------------------------------------------------------

>ZmPAP28b

-----------------------------------------------------------------------------------------------------------------MQSATRLTLLLCAA------------------------------------------------------------------------------------------------------------------------------------------------------------------------------------------------------------------------------------------------WAAALLYGEMGAYWVSYLAC-----------------------SWPSSSS--SPPN-----NHVKVA--------------------VVADPQ---LMDSTSLGLPSSSVALQAAEFYTDLNMRRSFQSTILPFKPDVVLFLGDHFDGG-------------------------------PYMPDEEWQESLFRFKHIFGLNDQRTKQQIPIYYLPGNHD----------------------IGYSA-------------------FHSVHPEVLSRYEKEFGS----------RNYQF--------------------SAG----KVDFVVVDAQTLDG----AKQSKERSSSWEFIK-TLSPGNT--SN--PK---------VLLTH-IP-----------LYRPDNSPCG-PHRSSPVINQRVSYAALDQGITYQNYLTKETSDLLLSLLKPILVLSGHDH--DQCTVVHSTP-------------------------------------------------------------------------------------------------------------------------------------------------------------------------------------------------------------------------------------------------------------------------------------------------------------------------------------------------------------

#Sequences and alignment used for Figure 2

>AtPAP5

MSLETFPPPAGYNAPEQVHITQGDHNGRGMIISWVTSLNEDGSNVVTYWIASSDGSDNKS

VIATTSSYRYFDYTSGYLHHAIIKELEYKTKYFYELGTGRSTRQFNLTPPKVGPDVPYTF

GVIGDLGQTYASNQTLYNYMSNPKGQAVLFAGDLSYADDHPNHDQSKWDSYGRFVEPSAA

YQPWIWAAGNHEIDYAQSIGETQPFKPYKNRYHVPYRASQNKYTPQNSWLQDEFKKVNRS

ETPWLIVLVHAPWYNSNNYHYMEGESMRVTFEPWFVENKVDIVFAGHVHAYERSERVSNI

QYNITDGMSTPVKDQNAPVYITIGDGGNIEGIANIFTDPQPSYSAFREASFGHALLEIKN

RTHAHYTWHRNKEDEAVIADSIWLKNRYYLPEEETI

>AtPAP6

MKNLVIFAFLFLSITTVINGGITSKFVRQALPSIEMSLDTFPSPGGYNTPEQVHLTQGDH

DGRGMIVSWVTPLNLAGSNVVTYWIATNGSDVKPAKKRAHASTKSYRFYDYSSGFLHHAT

IKGLEYDTKYIYEVGTDKSVRQFSFTTPPKIGPDVPYTFGIIGDLGQTYASNETLYHYMS

NPKGQAVLFAGDLSYADDHPNHDQRKWDTWGRFMEPCAAYQPFIFAAGNHEIDFVPNIGE

PHAFKPYTHRYPNAYKASQSTSPLWYSVRRASAHIIVLSSYSAYGKYTPQYIWLEQELKN

VNREETPWLIVIVHSPWYNSNNYHYMEGESMRVMFESWLVNSKVDLVLSGHVHAYERSER

ISNIKYNITNGLSSPVKDPNAPIYITIGDGGNIEGIANSFVDPQPSYSAYREASFGHAVL

EIMNRTHAQYTWHRNQDNEPVAADSIMLHNRHFFPVEEIVSSNIRA

>AtPAP10

MGRVRKSDFGSIVLVLCCVLNSLLCNGGITSRYVRKLEATVDMPLDSDVFRVPCGYNAPQ

QVHITQGDVEGKAVIVSWVTQEAKGSNKVIYWKENSTKKHKAHGKTNTYKFYNYTSGFIH

HCPIRNLEYDTKYYYVLGVGQTERKFWFFTPPEIGPDVPYTFGLIGDLGQSYDSNITLTH

YENNPTKGQAVLFVGDISYADTYPDHDNRRWDSWGRFAERSTAYQPWIWTTGNHELDFAP

EIGENRPFKPFTHRYRTPYRSSGSTEPFWYSIKRGPAYIIVLASYSAYGKYTPQYQWLEE

EFPKVNRTETPWLIVLMHSPWYNSYDYHYMEGETMRVMYEAWFVKYKVDVVFAGHVHAYE

RSERVSNIAYNVVNGICTPVKDQSAPVYITIGDGGNIEGLATKMTEPQPKYSAFREASFG

HAIFSIKNRTHAHYGWHRNHDGYAVEGDRMWFYNRFWHPVDDSPSCNS

>AtPAP11

MELSHLALVCAAIAFSSIFVVSQAGITSTHARVSEPSEEMSLETFPPPAGYNAPEQVHIT

QGDNAGRAMIISWVMPLNEDGSNVVTYWIASSDGSDNKNAIATTSSYRYFNYTSGYLHHA

TIKKLEYDPSKSRSRCSLHIRYYSDLGQTYASNQTLYNYMSNPKGQAVLFVGDLSYADDH

PNHDQRKWDSYGRFVEPSAAYQPWSWAAGNYEIDYAQSISETQPFKPYKNRYHVPYKASQ

STSPLWYSIKRASTYIIVLSSYSAYDKYTPQNSWLQDELKKVNRSETSWLIVLVHAPWYN

SNNYHYMEGESMRVTFEPWFVENKVDIVFAGHVHAYERSKRISNIHYNITDGMSTPVKDQ

NAPIYITIGDGGNIEGIANSFTDPQPSYSAFREASFGHALLEIKNRTHAHYTWHRNKEDE

AVIADSIWLKKRYYLPEEETA

>AtPAP12

MSSRSDLKIKRVSLIIFLLSVLVEFCYGGFTSEYVRGSDLPDDMPLDSDVFEVPPGPNSP

QQVHVTQGNHEGNGVIISWVTPVKPGSKTVQYWCENEKSRKQAEATVNTYRFFNYTSGYI

HHCLIDDLEFDTKYYYEIGSGKWSRRFWFFIPPKSGPDVPYTFGLIGDLGQTYDSNSTLS

HYEMNPGKGQAVLFVGDLSYADRYPNHDNNRWDTWGRFVERSVAYQPWIWTAGNHEIDFV

PDIGEIEPFKPFMNRYHTPHKASGSISPLWYSIKRASAYIIVMSCYSSYGIYTPQYKWLE

KELQGVNRTETPWLIVLVHSPFYSSYVHHYMEGETLRVMYEQWFVKYKVDVVFAGHVHAY

ERSERVSNIAYNIVNGLCEPISDESAPIYITIGDGGNSEGLLTDMMQPQPKYSAFREASF

GHGLLEIKNRTHAYFSWNRNQDGNAVAADSVWLLNRFWRAQKKTWLDAF

>AtPAP13

MVVKYTMSMSFFVIFASTVTIIVHGFPSTLDGPLNPVTAPLDPNLNPIAFDLPESDPSFV

KPISEFLLPEQISVSLSYSFDSVWISWVTGEYQIGEKDSAPLDPNCVQSIVQYREFDVRR

TRKQNATGHSIVYNQQYSSENGFMNYTSGIIHHVQLTGLKPNTLYRYQCGDPSLSAMSKE

YYFRTMPKSTSENYPHRIVVAGDLGLTYNTSTVLGHILSNHPDLVVLLGGFSYADTYLAN

KTKLDCSSCHCDQNGTSSDCGSCYSSGETYQPRWDYWGRFMEPLTANVPTMMVAGEHEIE

PQTENNLTFAAYSSRFAFPSNESADQYIWLESDLIKINRSETPWVVATWSLPWYSTFKGH

YREAESMRIHLEDLLYNYRVDIVFNSHVDAYERSNRVYNYTLDQCGPVYITTGAGGAGKL

ETQHVDDPGNIPDPSQNYSCRSSGLNSTLEPVKDETCPVKQPEYSAYRESSFGFGILEVK

NETHALWSWNRNQDLYYLAADVIHIVRQPEMCSVCN

>AtPAP15

MTFLLLLLFCFLSPAISSAHSIPSTLDGPFVPVTVPLDTSLRGQAIDLPDTDPRVRRRVI

GFEPEQISLSLSSDHDSIWVSWITGEFQIGKKVKPLDPTSINSVVQFGTLRHSLSHEAKG

HSLVYSQLYPFDGLLNYTSGIIHHVRITGLKPSTIYYYRCGDPSRRAMSKIHHFRTMPVS

SPSSYPGRIAVVGDLGLTYNTTDTISHLIHNSPDLILLIGDVSYANLYLTNGTSSDCYSC

SFPETPIHETYQPRWDYWGRFMENLTSKVPLMVIEGNHEIELQAENKTFEAYSSRFAFPF

NESGSSSTLYYSFNAGGIHFVMLGAYIAYDKSAEQYEWLKKDLAKVDRSVTPWLVASWHP

PWYSSYTAHYREAECMKEAMEELLYSYGTDIVFNGHVHAYERSNRVYNYELDPCGPVYIV

IGDGGNREKMAIEHADDPGKCPEPLTTPDPVMGGFCAWNFTPSDKFCWDRQPDYSALRES

SFGHGILEMKNETWALWTWYRNQDSSSEVGDQIYIVRQPDRCPLHHRLVNHC

>AtPAP19

MGLNHLTLVCSAIALLSIFVVSQAGVTSTHVRVSEPSEEMPLETFPPPACYNAPEQVHIT

QGDHAGRGMIISWVTPLNEDGSNVVTYWIANSDGSDNKSALATTSSYRYFNYTSGYLYHA

TIKGLETLYNYMSNPKGQAVLFAGDLSYADDHPNHDQRKWDSYGRFVEPSAAYQPWIWAA

GNHEIDYAESIPHKVHLHFGTKSNELQLTSSYSPLTQLMDELKKVNRSETPWLIVLVHAP

WYNSNNYHYMEGESMRVTFEPWFVENKVDIVFAGHVHAYERSERISNIQYNITDGMSTPV

KDQNAPVYITIGDGGNIEGIANNFIDPQPSYSAFREASFGHAILEIKNRTHAHYTWHRNK

EDEFIPEAVIADSIWLKNRYYLREEETS

>AtPAP23

MTLLIMITLTSISLLLAAAETIPTTLDGPFKPLTRRFEPSLRRGSDDLPMDHPRLRKRNV

SSDFPEQIALALSTPTSMWVSWVTGDAIVGKDVKPLDPSSIASEVWYGKEKGNYMLKKKG

NATVYSQLYPSDGLLNYTSGIIHHVLIDGLEPETRYYYRCGDSSVPAMSEEISFETLPLP

SKDAYPHRIAFVGDLGLTSNTTTTIDHLMENDPSLVIIVGDLTYANQYRTIGGKGVPCFS

CSFPDAPIRETYQPRWDAWGRFMEPLTSKVPTMVIEGNHEIEPQASGITFKSYSERFAVP

ASESGSNSNFYYSFDAGGVHFVMLGAYVDYNNTGLQYAWLKEDLSKVDRAVTPWLVATMH

PPWYNSYSSHYQEFECMRQEMEELLYQYRVDIVFAGHVHAYERMNRIYNYTLDPCGPVYI

TIGDGGNIEKVDVDFADDPGKCHSSYDLFFFNSLNLSN

>AtPAP25

MRMNKILLVFVFLSIATVINSGTTSNFVRTAQPSTEMSLETFPSPAGHNAPEQVHIVQGD

YNGRGIIISWVTPLNLAGSNVVTYWKAVDGDVKPKKKRGHASTSSYRFYDYTSGFLHHAT

IKGLEYDTKYIYEVGTDGSVRQFSFTSPPKVGPDVPYTFGIIGDLGQTLASNETLYHYMS

NPKGQAVLFPGDLSYADDHPNHDQRKWDSWGRFVEPCAAYQTFIYAAGNHEIDFVPNIGE

PHAFKPYIHRYHNAYKASKSISPLWYSIRRASAHIIVLSSYSAYGKYTPQYVWLEQELKK

VNREETPWLIVMVHSPWYNSNNYHYMEGESMRAMFESWFVNSKVDLVLSGHVHSYERSER

VSNIKYNITNGLSYPVKDPSAPIYITIGDGGNIEGIANSFTDPQPSYSAYREASFGHAVL

EIYNRTHAYYTWHRNQDNEPVAADSIMLHNRYFFPVEELESGNTRA

>AtPAP26

MNHLVIISVFLSSVLLLYRGESGITSSFIRSEWPAVDIPLDHHVFKVPKGYNAPQQVHIT

QGDYDGKAVIISWVTPDEPGSSQVHYGAVQGKYEFVAQGTYHNYTFYKYKSGFIHHCLVS

DLEHDTKYYYKIESGESSREFWFVTPPHVHPDASYKFGIIGDMGQTFNSLSTLEHYMESG

AQAVLFLGDLSYADRYQYNDVGVRWDSWGRFVERSTAYQPWLWSAGNHEVDYMPYMGEVT

PFRNYLQRYTTPYLASKSSSPLWYAVRRASAHIIVLSSYSPFVKYTPQWHWLSEELTRVD

REKTPWLIVLMHVPIYNSNEAHFMEGESMRAAFEEWFVQHKVDVIFAGHVHAYERSYRIS

NVRYNVSSGDRYPVPDKSAPVYITVGDGGNQEGLAGRFTEPQPDYSAFREASYGHSTLDI

KNRTHAIYHWNRNDDGKKVATDEFVLHNQYWGKNIRRRKLKKHYIRSVVGGWIAT

>SbPAP10

MGRHGVDQIGAVAAAFAWVVLLPLLLVGVVCPGAQAGQTSEYRRQLGSAIDMPLDADVFR

PPPGHNAPEQVHITQGNHDGTAMIISWVTTSEPGSSTVIYGTSEDNLNYTANGKHTQYTF

YNYTSGYIHHCTIKKLEFDTKYYYAVGIGQTVRKFWFMTPPESGPDVPYTFGLIGDLGQS

FDSNVTLTHYESNAKAQAVLFVGDLSYADNYPYHDNVRWDTWARFVERNVAYQPWIWTAG

NHEIDFAPELGETKPFKPFSQRYPTPYKASGSTAPYWYSIKRASAYIIVLASYSAYGKYT

PQYKWLEAEFPKVNRSETPWLIVLMHAPWYNSYNYHYMEGETMRVMYEPWFVKYKVDVVF

AGHVHAYERTHRISNVAYNVVNGLCTPISDQSAPVYITIGDGGNQEGLATNMSQPQPRYS

AFREASFGHAILDIKNRTHAYYTWHRNQDGSSVAADSMWFTNRYWEPTDDSADDFQ

>SbPAP30a

MMGDDLLLRRRGGVLFLVLSAVLVLLGGGADAGVTSSYRRKLEATVEMPLDADVFRVPPG

YNAPQQVHITLGDQEGTAMTVSWVTASELGNSTVKFGEKPDPEKMERRAEGTHTRYDYFN

YTSGFIHHCTLKHLKHSTKYYYAMGFGHTVRTFSFTTPPKPGPDVPFKFGLIGDLGQTFD

SNITLSHYDDNGGDAVLYVGDLSYADNHPLHDNNRWDTWGRFMERNAAYQPWIWTAGNHE

LDFAPELGETTPFKPYTHRYPTPYLSSGSTEPFWYSVKLASAHVVVLSSYSAYGKYTPQW

TWLQAELQRVDRTTTPWLVVLMHSPWYNSNNYHYMEGETMRVQFEKWLVDARADVVLAGH

VHSYERSHRVSNVAYDIVNGEATPVRDAGAPVYVTIGDGGNIEGVADNFTRPQPSYSAFR

EASFGHATLEIKNRTHAYYSWHRNHDGAKVVADGVWLTNRAQSYAVFFLDRTKTSSMAID

HQP

>SbPAP30c

MHTVAIQRRRHGAVTLLLVAAAVVLLSAAAQTAAAHRPRGCGVTSKYVRKHQASEDMPLD

ADVFAVPPGRNAPQQVHIGLADQTGTSMFVSWVTVEAEGNSTVLYGLAADKLDLAAEGTI

TRYTYYNYTSGYIHHATLTNLQHGTRYHYAVGVGVGDTVRAFWFTTPPAPGPDVPLRLGL

IGDLGQTADSNSTLAHYESHQGDAVLFVGDLSYADKHPLHDNNRWDTWGRFAERSVAYQP

WVWTTGNHEVDFAPELGELAPFKPFTHRYPTPWKASQSSEPYWYSVKLGPAHIIVLSSYS

AFGKYTPQYKWVEAELKRVDREVTPWLFISTHVPWYNSNNFHYMEGEPMRAQLEKMAVDA

RVDAVFAGHVHAYERTHRYSNIKYNVTDGKCTPIADRRAPVYVVIGDGGNVEGLADELTW

PQPAYSAFREYSFGHAVLDIKNRTHAYYAWYRNHDGNKVTADTMWFTNRYHMPNHDDSMT

MITAKAAC

>SbPAP30b

MSNMLGARRRLGLVHVVLVLFAAALLVADAGVTSSYRRKLEATVEMPLDADVFGVPLGYN

APQQVHITLGDIEGTSMIVSWVTANELGSSTVFYSEASPDPYMMELWAEGTHTRYNYFNY

TSGFIHHCNLTNLKYGTKYYYAMGFGHTVRSFSFTTPPMPGPDVPFKFGLIGDLGQTFDS

NTTLSHYEANGGGAVLYVGDLSYADNRPLHDNTRWDTWARFVERSAAHQPWVWTVGNHEL

DLAPELGEPVPFKPFAHRYPTPRRFAPAAAAPPFWYSVRIASAHVIVLASYSAYGKYTPQ

WKWLRGELARVDRAATPWLIVLVHSPWYSSNGYHYMEGETMRVQFERWLVAAKADLVVAG

HVHAYERSHRVSNVAYDIVNGKCTPVRSRDAPVYVTVGDGGNIEGVADNFTQPQPGYSAF

REASFGHATLEIMNRTHAYYAWHRNQDGAMVVADGVWFTNRYWMPTDDDDTS

>SbPAP26

MRGWGLLVLSLHVLSCLVSGVASGRTSSYVRTEFPSTDIPLDSEWFAIPKGYNAPQQVHI

TQGDYDGKAVIVSWVTPEEPGPSEVFYGKEKQYDQKSEGTTTNYTFYDYKSGYIHHCLVD

GLEYNTKYYYKIGSGDSAREFWFETPPAIDPDASYTFGIIGDLGQTFNSLSTLQHYEKTG

GQTVLFVGDLSYADRYEHNDGIRWDSWGRFVERSTAYQPWIWNTGNHEIEYRPDLGETST

FKPYLHRYMTPYLASKSSSPMWYAVRRASAHIIVLSSYSPFVKYTPQWWWLKNEFKRVDR

EKTPWLIVLMHSPMYNSNEAHYMEGESMRAAFEKWFVKYKVDLVFAGHVHAYERSYRISN

VNYNITSGNRYPVPNKSAPVYITVGDGGNQEGLASRFYDPQPDYSAFREASYGHSLLQLK

NRTHAVYQWNRNHDGNPVPADTVVFHNQYWTSSTRRRRLKKNHFHLENLEDLISLF

>ZmPAP26

MRGWGLLVLSLHVLSCLVSGVASGRTSSYVRTEFPSTDIPLESEWFAIPKGYNAPQQVHI

TQGDYDGKAVIVSWVTPEEPGPSEVFYGKEKLYDQKAEGTTTNYTFYDYKSGYIHHCLVD

GLEYNTKYYYKIGSGNSAREFWFETPPAIDPDASYTFGIIGDLGQTFNSLSTLQHYEKTG

GQTVLFVGDLSYADRYEHNDGIRWDSWGRFVEHSTAYQPWIWNTGNHEIEYRPDLGETSV

FKPYLHRYMTPYLASKSSSPMWYAVRRASAHIIVLSSYSPFVKYTPQWLWLKNEFKRVDR

EKTPWLIVLMHSPMYNSNEAHYMEGESMRAAFEKWFVKYKVDLVFAGHVHAYERSYRISN

VNYNITSGNRYPVPDKSAPVYITVGDGGNQEGLASRFYNPQPDYSAFREASYGHSVLQLK

NRTHAIYQWNRNDDGNPVPADTVMFHNQYWTSSTRRRRLKKNHLHLEDLEDLISLL

>ZmPAP10

MGRHGVDQIGAVAAFVWVTLLPLLLVCVVWPGAQAGHTSEYRRQLGSAIDMPLDADVFRP

PPGYNAPEQVHITQGNHDGTAMIISWVTTSEPGSSTVIYGTSEDNLNYTANGKHTQYTFY

NYTSGYIHHCTIKKLEFDTKYYYAVGIGQTVRKFWFLTPPKSGPDVPYTLGLIGDLGQSF

DSNVTLTHYESNAKAQAVLFVGDLSYADNYPYHDNVRWDTWARFVERSVAYQPWIWTAGN

HEIDFAPELGETKPFKPFSHRYPTPYKASGSTAPYWYSIKRASAYIIVLASYSAYGKYTP

QYKWLEAEFPKVNRSETPWLVVLMHAPWYNSYNYHYMEGETMRVMYEPWFVKYKVDVVFA

GHVHAYERTHRISNVAYNVVNGLCTPIPDQSAPVYITIGDGGNQEGLATNMSQPQPSYSA

FREASFGHAILDIKNRTHAYYTWHRNQDGSAVAADSMWFTNRYWEPTDDSADDFQ

>ZmPAP30a

MMGPLPLRRRVGVLLFLALVLLGGGGGADAGTTSSYRRKLEATVEMPLDADVFRVPPGYN

APQQVHITLGDQEGTAMIVSWVTASEPGNSTVAYGEDPARMERRADGAHTRYDYFNYTSG

FIHHCTLRNLKHATKYYYAMGFGHTVRTFWFTTPPKPGPDVPFKFGLIGDLGQTFDSNIT

LSHYESNGGDAVLYVGDLSYADNHPLHDNNRWDTWARFVERSVAYQPWVWTAGNHELDFA

PELGETTPFKPFAHRYPTPYRAAGSTEPFWYSVKVASAHVVVLASYSAYGKYTPQWAWLQ

AELARVDRKTTPWLVVLTHSPWYNSNNYHYMEGETMRVQFERWLVDAKVDLVLAGHVHSY

ERSHRVSNVAYDIVNGKSTPVRSADAPVYVTIGDGGNIEGIADNFTRPQPGYSAFREASF

GHATLDIKNRTHAYYSWHRNHDGAKVVADGVWFTNRYWMPTDDDTN

>ZmPAP30b

MSNVLRARRRLDLLQVVLFFVAVLLVVVADAGVTSQYRRKLEATVEMPLDADVFRVPPGY

NAPQQVHITLGDQEGTAMIVSWVTANELGSSTVMYSEASPDPEKMELRAEGTHTRYDYFN

YTSGFIHHCTLTNLKHSTKYYYAMGFGHTVRSFCFTTPPMPGPDVPFKFGLIGDLGQTFD

SNTTLSHYEANGGDAVLYVGDLSYADNHPLHDNTRWDTWARFVERSAAHQPWVWTAGNHE

LDLAPELGEHVPFKPFAHRYPTPFWYSVRVASAHVVVLASYSAYGKYTAQWEWLRAELAR

VDRAATPWLIVLVHSPWYSSNGYHYMEGETMRVQFERWIVAAKADLVVAGHVHAYERSHR

VSNVAYDIINARCTPVRTRDAPVYVTVGDGGNIEGIADNFTQPQPSYSAFREASFGHATL

EIRNRTHAYYAWHRNQDGAKVVADGVWLTNRYWMPTDDDIN

>OsPAP10a

MVDRIGAAWWCACAVGMLVVGACLAGETSEYRRQLGSAVDMPLDADVFRAPPGRNAPQQV

HITQGNHDGTAMIISWVTTIEPGSSTVLYGTSEDNLNFSADGKHTQYTFYNYTSGYIHHC

TIKKLEFDTKYYYAVGIGQTVRKFWFRTPPKSGPDVPYTFGLIGDLGQSYDSNITLAHYE

SNSKAQAVLFVGDLCYADNYPYHDNVRWDTWARFVERNVAYQPWIWTAGNHEIDFAPELG

ETKPFKPYSYRYPTPYKASGSTAPFWYSVKRASAYIIVLASYSSYGKYTPQYKWLEAEFP

KVNRSETPWLIVLLHAPWYNSYNYHYMEGESMRVMYEPWFVKYKVDLVFAGHVHAYERTH

RISNVAYNIVNGQCTPVHDQSAPVYITIGDGGNQEGLATNMTAPQPGYSAFRESSFGHAI

LDIKNRTHAYYTWHRNQDGNAVAADSMWFTNRYWQPTDESLDDSQ

>OsPAP10b

MVMEAACVLAVVVVVMAFLSPAARGGVTSTYRRSLQALPDMPIDADVFRPPPGFNAPEQV

HITLGDQTGRAMTVSWVTPKLPDSNVVRYGLRADNLTHTANGTFRRYSFGRKYRSGFIHH

ATLTGLDYGTKYHYAVGSGDTASARSFSFTTPPKPGPDVPYKFGLIGDLGQTFHSNDTLS

HYEACGGDAVLFIGDLSYADNHPGHDNNRWDTWARFVERSVAYQPWIWTTGNHELDFAPE

LGETTPFKPFTNRYPTPFGASGSTRPLWYSVRMASAHVIVLASYAAYGKYTPQWRWLEGE

LRRVDRAVTPWLIVCVHSPWYSSNGYHYMEGESMRVEFERWLVDAKADVVLAGHVHSYER

TRRVSNVAYDIANGMATPVFNRSAPVYINIGDGGNIEGLADDFRWPQPDYSVFREASFGH

ATLQIVNRTHAFYEWHRNSDGVKVVADHAWFTNRYWFPTDTN

>OsPAP10c

MGMLRWGAHLLLLLLAAATWTCAGAGAGVTSEYRRKLEATVDMPLDADVFRVPPGYNAPQ

QTGTAMTVSWVTANELGSNTVRYGSSPEKLDRAAEGSHTRYDYFNYTSGFIHHCTLTGLT

HATKYYYAMGFDHTVRTFSFTTPPKPAPDAPFKFGLIGDLGQTFDSNSTLAHYEANGGDA

VLFVGDLSYADNYPLHDNNRWDTWARFVERSVAYQPWIWTAGNHELDYAPELGETVPFKP

FTHRYPTPYRAAGSTEPFWYSVKIASAHVIVLASYSAYGKYTPQWTWLQEELATRVDRKL

TPWLIVLMHSPWYNSNNYHYMEGETMRVQFERWLVDAKVDVVLAGHVHSYERSRRFANID

YNIVNGKATPAANVDAPVYITIGDGGNIEGIANNFTVPQPAYSAFREASFGHATLEIKNR

THAHYAWHRNHDGAKAVADAVWLTNRYWMPTNDDV

>OsPAP10d

MGFGFTVRSFWFTTPPRPGPDVAFRLGLIGDIGQTFDSNATLTHYEASGGDAVLFMGDLS

YADKYPLHDNNRWDTWGRFSERSVAYQPWIWVAGNHEIDYAPELGETKPFKPFTHRYPTP

HLASASPEPYWYSVKLASVHIIVLSSYSAFAKYTPQWKWLEAELGRVNRSETPWLIMASH

SPWYNSNNFHYMEGESMRAQLEKMAVDARVDLVFAGHVHAYERSFRVSNIRYNITDGLCT

PVRDRRAPVYVTIGDGGNIEGLADEMTWPQPPYSAFREDSFGHAVLDIKNRTHAYYAWYR

NDDGAKVAADAVWFTNRFHMPNHDDSTPTPTKRHYYG

>OsPAP26

MGWRFALLLLHVLLCLVNGVSCGRTSSYVRTEYPSTDIPLESEWFAVPNGYNAPQQVHIT

QGDYNGKAVIVSWVTVAEPGTSEVLYGKNEHQYDQRVEGTVTNYTFYDYKSGYIHHCLVD

GLEYNTKYYYKIGSGDSAREFWFETPPAIDPDASYTFGIIGDLGQTFNSLSTLQHYEKSE

GQTVLFVGDLSYADRYQHNDGVRWDSWGRLVERSTAYQPWIWSAGNHEIEYRPDLGETST

FKPYLHRCHTPYLASKSSSPMWYAVRRASAHIIVLSSYSPFVKYTPQWTWLKYELKHVDR

EKTPWLIVLMHSPMYNSNEAHYMEGESMRAAFEKWFVKYKVDLVFAGHVHAYERSYRISN

INYNITSGNRYPVPDKSAPVYITVGDGGNQEGLASRFSDPQPDYSAFREASYGHSILQLK

NRTHAIYQWNRNDDGKHVPADNVVFHNQYWASNTRRRRLKKKHFHLDQIEDLISVF

>BrPAP10a

MKSDFSSVLLVLGLVLNSLVLFCNGGITSKFVRKVEKSIDMPLHSDVFRVPPGHNAPQQV

HITQGDVEGKAVIVSWVTQEAPGSNTVLYWKEHSSKKHKAHGKTNTYKFYNYTSGYIHHC

TIRNLEYDSKYYYVVGVGQTERKFWFFTPPKVGPDVPYTFGLIGDLGQTFDSNITLTHYE

KSPKKGQAVLFVGDLSYADNHPNHDNNRWDSWGRFAERSTAYQPWIWTTGNHELDFAPEI

GENKPFKPFTHRYRTPYRASGSTEPFWYSIKRGPAYIIVLSSYSAYGKYTPQYTWLEEEF

PKVNRTETPWLIVLNHSPWYNSYDYHYMEGETMRVMYEPWFVKNKVDVVFSGHVHAYERS

ERISNIAYTVVNGICSPVKDQSAPVYITIGDGGNIEGLATKMTEPQPKYSAYREASFGHA

IFSIKNRTHAHYAWHRNQDGYAVEADTMWFYNRFWHPVNDSPSSDS

>BrPAP10b

MGLRLEGPNLFLLLILGMVLNSLVLFCHGGRTSSYVRRLEATVDMPLDSDVFRVPPGYNA

PQQVHITQGDVEGKAVIVSWVTQEAPGSDTVLYWKENSSKKLKAYGKSKTYKFYNYTSGH

IHHCTIRNLEYDTKYYYVVGVGQTEREFYFFTPPEVGPDVPYTFGLIGDLGQSFDSNITL

THYENNPTKGQAVLFVGDFSYADQYPNHDNNRWDTWGRFAERSTAYQPWIWTVGNHELDF

APELGETKPFKPFLNRYRTPYRSSGSTEPFWYSIKRGPAYIIVLASYSAYGKYTPQFKWL

EKEFPKVNRTETPWLIVLMHSPWYNSYDYHYMEGETMRVMYEPWFVKYKVDVVFAGHVHA

YERSERVSNIAYNVVNGICTPVKDKSAPVYITIGDGGNLEGLATRMTEPQPKYSAFREAS

FGHAVLSIKNRTHAYYGWHRNQDGYAVKGDTMWFYNRFWHPIDDSPSE

>BrPAP10c

MKMGRFEGFFLSFVLNSFLLFCHGGTTSRFVRRLEATADMPLDSDVFRVPPGCNAPQQVH

ITQGDLEGKAVIVSWVTQKAKGSNMVLYWKEHSSKMLKAHGKSKTYKFYNYTSGHIHHCT

IRNLEYDTKYYYMVGVGQTERKFWFLTPPKPGPDVPYTFGLIGDLGQSFDSNITLTHYEN

NPLKGQTILFVGDFSYADTYPNHDNNRWDTWGRFVERSTAYQPWIWTVGNHELDFAPQIG

ETKPFKPFKHRYHTPHRSSGSTEPFWYSIKRGPAYIIVLASYSAYGKYTPQYMWLEQEFP

KVNRTETPWLIVLMHSPWYNSYDYHYMEGETMRVMYEPWFVKNKVDVVFAGHVHGYERSE

RISNIAYNVVNGICSPVKDLSAPVYITIGDGGNLEGLATIMTEPQPKYSAFREASFGHAI

FSIKNRTHAYYGWHRNQDGCAVDGDTMWFFNRIWHPIDDSPDDDS

>BrPAP12

MPLDSNVFIPDDRPLHSDVFLPDQKNKDSDVFVPDDMPLDSDVFKVPPGPNTPQQVHITQ

VRYWSENGKLKKLAEATINTYRFFNYTSGYIHHCLIDDLEIFNLFLIQYPGDLGQTYDSN

RTLSHYEMNPGKGQAVLFLGDLSYADLYKFHDNNRWDTWGRFVERSAAYQPWIWTAGNHE

IDFVPDIGETEPFKPFTNRYHTPYKASGSISPLWYSIKRASAYIIVMSCYSSYGVYTPQY

KWLRKEFQRVNRTETPWLIVLVHCPFYHSYVHHYMEGETMRVLYEQWFVKSKVDVVFAGH

VHAYERSYLLCPQVIINY

>BrPAP26

MIRGMEIHRLLVVVVAVLLSLVLNGEGGITSSYVRSEWPAVDIPLDHKVFKVPKGYNAPQ

QVHITQGDYDGKAVIISWVTPDEPGSSTVHYGPMQGEYDFVAKGSYSNYTFYKYKSGYTH

HCLLSGLEYNTKYYYKIESGESSREFWFVTPPHVHPDASYKFGIIGDLGQTFNSLSTLEH

YMRSGAQAVLFLGDLSYADRYQYNDVGVRWDTWGRFVEPSTAYQPWLWSAGNHEVDYMPY

MGEVTPFKNYLERYTTPYLASKSSNPLWYAVRRASAHIIVLSSYSPFVKYTPQWRWLDAE

FKRVDREKTPWLIVLMHSPIYNSNEAHFKEGESMRAAFEEWFVEYRVDVVFAGHVHAYER

SYRVSNVRYNVSSGDRFPVPDTSAPVYITVGDGGNQEGLAGRFMEPQPDYSAFREASYGH

STLDIKNRTHAVYHWNRNDDGKKVATDEFVLYNQYWGKNIRRRKLKKHYIKSVVVDWIAS

>AtPAP5

--------------------------------------------------------------MSLET--FPPP-AGYNAPEQVHITQGDHNGRGMIISWVTSLNEDG------------SNVVTYWIASSDGSD----NKSVIATTSSY--------RY-FDYTSGYLHHAIIKELEYKTKYFYE--LG-TGRS--TRQFNL-TPPKVGPDV-PYTFGVIGDLGQTYASNQTLYNYMSNP-KGQAVLFAGDLSYADDHPN-----------------------------HD-QSKWDSYGRFVEPSAAYQPWIWAAGNHEIDYAQSIGETQPFKPYKNRYHVP--YRASQN-------------------------KYTPQNSWLQDEFK-KVNRSETPWLIVLVHAPWYNSNNYHYMEGESMRVTFEPWFVENKVDIVFAGHVHAYERSERVSNIQYNITDGMSTPVKDQNAPVYITIGDGGNIEGIANIFTD---------------------------------PQPSYSAFREASFGHALLEIKNRTHAHYTWHRNKED----EAVIADSIWLKNR------YY--------LPEEETI-----------

>AtPAP6

-------------MKNLVIFAFLFLSITTVI------------NGGITSKFVRQAL--PSIEMSLDT--FPSP-GGYNTPEQVHLTQGDHDGRGMIVSWVTPLNLAG------------SNVVTYWI-ATNGSDVKPAKKRAHASTKSY--------RF-YDYSSGFLHHATIKGLEYDTKYIYE--VG-TDKS--VRQFSFTTPPKIGPDV-PYTFGIIGDLGQTYASNETLYHYMSNP-KGQAVLFAGDLSYADDHPN-----------------------------HD-QRKWDTWGRFMEPCAAYQPFIFAAGNHEIDFVPNIGEPHAFKPYTHRYPNA--YKASQSTSPLWYSVRRASAHIIVLSSYSAYGKYTPQYIWLEQELK-NVNREETPWLIVIVHSPWYNSNNYHYMEGESMRVMFESWLVNSKVDLVLSGHVHAYERSERISNIKYNITNGLSSPVKDPNAPIYITIGDGGNIEGIANSFVD---------------------------------PQPSYSAYREASFGHAVLEIMNRTHAQYTWHRNQDN----EPVAADSIMLHNR------HF--------FPVEEIVSSNIRA-----

>AtPAP10

------MGRVRKSDFGSIVLVLCCVLNSLLC------------NGGITSRYVRKLE--ATVDMPLDSDVFRVP-CGYNAPQQVHITQGDVEGKAVIVSWVTQ-EAKG------------SNKVIYWK---ENSTK---KHKAHGKTNTY--------KF-YNYTSGFIHHCPIRNLEYDTKYYYV--LG-VGQT--ERKFWFFTPPEIGPDV-PYTFGLIGDLGQSYDSNITLTHYENNPTKGQAVLFVGDISYADTYPD-----------------------------HD-NRRWDSWGRFAERSTAYQPWIWTTGNHELDFAPEIGENRPFKPFTHRYRTP--YRSSGSTEPFWYSIKRGPAYIIVLASYSAYGKYTPQYQWLEEEFP-KVNRTETPWLIVLMHSPWYNSYDYHYMEGETMRVMYEAWFVKYKVDVVFAGHVHAYERSERVSNIAYNVVNGICTPVKDQSAPVYITIGDGGNIEGLATKMTE---------------------------------PQPKYSAFREASFGHAIFSIKNRTHAHYGWHRNHDG----YAVEGDRMWFYNR------FW--------HPVDDSPSCNS-------

>AtPAP11

---------MELSHLALVCAAIAFSSIFVVS------------QAGITSTHARVSE--PSEEMSLET--FPPP-AGYNAPEQVHITQGDNAGRAMIISWVMPLNEDG------------SNVVTYWIASSDGSD----NKNAIATTSSY--------RY-FNYTSGYLHHATIKKLEYD-------------------------PSKSRSRC-SLHIRYYSDLGQTYASNQTLYNYMSNP-KGQAVLFVGDLSYADDHPN-----------------------------HD-QRKWDSYGRFVEPSAAYQPWSWAAGNYEIDYAQSISETQPFKPYKNRYHVP--YKASQSTSPLWYSIKRASTYIIVLSSYSAYDKYTPQNSWLQDELK-KVNRSETSWLIVLVHAPWYNSNNYHYMEGESMRVTFEPWFVENKVDIVFAGHVHAYERSKRISNIHYNITDGMSTPVKDQNAPIYITIGDGGNIEGIANSFTD---------------------------------PQPSYSAFREASFGHALLEIKNRTHAHYTWHRNKED----EAVIADSIWLKKR------YY--------LPEEETA-----------

>AtPAP12

-----MSSRSDLKIKRVSLIIFLLSVLVEFC------------YGGFTSEYVRGSD--LPDDMPLDSDVFEVP-PGPNSPQQVHVTQGNHEGNGVIISWVTP-VKPG------------SKTVQYWC---ENEKS---RKQAEATVNTY--------RF-FNYTSGYIHHCLIDDLEFDTKYYYE--IG-SGKW--SRRFWFFIPPKSGPDV-PYTFGLIGDLGQTYDSNSTLSHYEMNPGKGQAVLFVGDLSYADRYPN-----------------------------HD-NNRWDTWGRFVERSVAYQPWIWTAGNHEIDFVPDIGEIEPFKPFMNRYHTP--HKASGSISPLWYSIKRASAYIIVMSCYSSYGIYTPQYKWLEKELQ-GVNRTETPWLIVLVHSPFYSSYVHHYMEGETLRVMYEQWFVKYKVDVVFAGHVHAYERSERVSNIAYNIVNGLCEPISDESAPIYITIGDGGNSEGLLTDMMQ---------------------------------PQPKYSAFREASFGHGLLEIKNRTHAYFSWNRNQDG----NAVAADSVWLLNR------FW--------RAQKKTWLDAF-------

>AtPAP13

----------MVVKYTMSMSFFVIFASTVTIIVHGFPSTLDGPLNPVTAPLDPNLNP-IAFDLPESDPSFVKPISEFLLPEQISVSL-SYSFDSVWISWVTGEYQIGEKDSAPLDPNCVQSIVQYREFDVRRTR----KQNATGHSIVYNQQYSSENGF-MNYTSGIIHHVQLTGLKPNTLYRYQ--CGDPSLSAMSKEYYFRTMPKSTSENYPHRIVVAGDLGLTYNTSTVLGHILSN--HPDLVVLLGGFSYADTYLA-NKTKLDCSSCHCDQNGTSSDCGSCYSSGETYQPRWDYWGRFMEPLTANVPTMMVAGEHEIE--PQTENNLTFAAYSSRFAFP--SNES-----------------------------ADQYIWLESDLI-KINRSETPWVVATWSLPWYSTFKGHYREAESMRIHLEDLLYNYRVDIVFNSHVDAYERSNRVYN--YTL---------DQCGPVYITTGAGGA-GKLETQHVDDPGNIPDPSQNYSCRSSGLNSTLEPVKDETCPVKQPEYSAYRESSFGFGILEVKNETHALWSWNRNQDL----YYLAADVIHIVRQ-----------------PEMCSVCN---------

>AtPAP15

--------------MTFLLLLLFCFLSPAISSAHSIPSTLDGPFVPVTVPLDTSLRG-QAIDLPDTDPRVRRRVIGFE-PEQISLSL-SSDHDSIWVSWITGEFQIG-KKVKPLDPTSINSVVQFGT---LRHSL---SHEAKGHSLVYSQLYPFD-GL-LNYTSGIIHHVRITGLKPSTIYYYR--CGDPSRRAMSKIHHFRTMPVSSPSSYPGRIAVVGDLGLTYNTTDTISHLIHN--SPDLILLIGDVSYANLYLT-NGTSSDCYSCSFPETPIH----------ETYQPRWDYWGRFMENLTSKVPLMVIEGNHEIELQ---AENKTFEAYSSRFAFP--FNESGSSSTLYYSFNAGGIHFVMLGAYIAYDKSAEQYEWLKKDLA-KVDRSVTPWLVASWHPPWYSSYTAHYREAECMKEAMEELLYSYGTDIVFNGHVHAYERSNRVYN--YEL---------DPCGPVYIVIGDGGNREKMAIEHADDPGKCPEPLTTPDPVMGGFCAWNFTPSDKFCWDRQPDYSALRESSFGHGILEMKNETWALWTWYRNQDS----SSEVGDQIYIVRQ-------------PDRCPLHHRLVNHC-------

>AtPAP19

---------MGLNHLTLVCSAIALLSIFVVS------------QAGVTSTHVRVSE--PSEEMPLET--FPPP-ACYNAPEQVHITQGDHAGRGMIISWVTPLNEDG------------SNVVTYWIANSDGSD----NKSALATTSSY--------RY-FNYTSGYLYHATIKGLE------------------------------------------------------TLYNYMSNP-KGQAVLFAGDLSYADDHPN-----------------------------HD-QRKWDSYGRFVEPSAAYQPWIWAAGNHEIDYAESI-------PHKVHLHFG--TKSNE---------------------LQLTSSYSPLTQ-LMDELK-KVNRSETPWLIVLVHAPWYNSNNYHYMEGESMRVTFEPWFVENKVDIVFAGHVHAYERSERISNIQYNITDGMSTPVKDQNAPVYITIGDGGNIEGIANNFID---------------------------------PQPSYSAFREASFGHAILEIKNRTHAHYTWHRNKEDEFIPEAVIADSIWLKNR------YY--------LREEETS-----------

>AtPAP23

--------------MTLLIMITLTSISLLLAAAETIPTTLDGPFKPLTRRFEPSLRR-GSDDLPMDHPRLRKRNVSSDFPEQIALAL--STPTSMWVSWVTGDAIVG-KDVKPLDPSSIASEVWYGK---EKGNY---MLKKKGNATVYSQLYPSD-GL-LNYTSGIIHHVLIDGLEPETRYYYR--CGDSSVPAMSEEISFETLPLPSKDAYPHRIAFVGDLGLTSNTTTTIDHLMEN--DPSLVIIVGDLTYANQYRTIGGKGVPCFSCSFPDAPIR----------ETYQPRWDAWGRFMEPLTSKVPTMVIEGNHEIE--PQASGIT-FKSYSERFAVP--ASESGSNSNFYYSFDAGGVHFVMLGAYVDYNNTGLQYAWLKEDLS-KVDRAVTPWLVATMHPPWYNSYSSHYQEFECMRQEMEELLYQYRVDIVFAGHVHAYERMNRIYN--YTL---------DPCGPVYITIGDGGNIEKVDVDFADDPGK-----------------------------CHSSY----------------------------------------DLFFFNS-----------------LNLSN-------------

>AtPAP25

------------MRMNKILLVFVFLSIATVI------------NSGTTSNFVRTAQ--PSTEMSLET--FPSP-AGHNAPEQVHIVQGDYNGRGIIISWVTPLNLAG------------SNVVTYWK--AVDGDVKPKKKRGHASTSSY--------RF-YDYTSGFLHHATIKGLEYDTKYIYE--VG-TDGS--VRQFSFTSPPKVGPDV-PYTFGIIGDLGQTLASNETLYHYMSNP-KGQAVLFPGDLSYADDHPN-----------------------------HD-QRKWDSWGRFVEPCAAYQTFIYAAGNHEIDFVPNIGEPHAFKPYIHRYHNA--YKASKSISPLWYSIRRASAHIIVLSSYSAYGKYTPQYVWLEQELK-KVNREETPWLIVMVHSPWYNSNNYHYMEGESMRAMFESWFVNSKVDLVLSGHVHSYERSERVSNIKYNITNGLSYPVKDPSAPIYITIGDGGNIEGIANSFTD---------------------------------PQPSYSAYREASFGHAVLEIYNRTHAYYTWHRNQDN----EPVAADSIMLHNR------YF--------FPVEELESGNTRA-----

>AtPAP26

------------MNHLVIISVFLSSVLLLYRG-----------ESGITSSFIRSEW--PAVDIPLDHHVFKVP-KGYNAPQQVHITQGDYDGKAVIISWVTP-DEPG------------SSQVHYGA---VQGKY---EFVAQGTYHNY--------TF-YKYKSGFIHHCLVSDLEHDTKYYYK--IE-SGES--SREFWFVTPPHVHPDA-SYKFGIIGDMGQTFNSLSTLEHYMES--GAQAVLFLGDLSYADRYQY-----------------------------NDVGVRWDSWGRFVERSTAYQPWLWSAGNHEVDYMPYMGEVTPFRNYLQRYTTP--YLASKSSSPLWYAVRRASAHIIVLSSYSPFVKYTPQWHWLSEELT-RVDREKTPWLIVLMHVPIYNSNEAHFMEGESMRAAFEEWFVQHKVDVIFAGHVHAYERSYRISNVRYNVSSGDRYPVPDKSAPVYITVGDGGNQEGLAGRFTE---------------------------------PQPDYSAFREASYGHSTLDIKNRTHAIYHWNRNDDG----KKVATDEFVLHNQ------YWGKNIRRRKLKKHYIRSVVGGWIAT--

>SbPAP10

MGRHGVDQIGAVAAAFAWVVLLPLLLVGVVCPGA---------QAGQTSEYRRQLG--SAIDMPLDADVFRPP-PGHNAPEQVHITQGNHDGTAMIISWVTT-SEPG------------SSTVIYGT---SEDNL---NYTANGKHTQY--------TF-YNYTSGYIHHCTIKKLEFDTKYYYA--VG-IGQT--VRKFWFMTPPESGPDV-PYTFGLIGDLGQSFDSNVTLTHYESNA-KAQAVLFVGDLSYADNYPY-----------------------------HD-NVRWDTWARFVERNVAYQPWIWTAGNHEIDFAPELGETKPFKPFSQRYPTP--YKASGSTAPYWYSIKRASAYIIVLASYSAYGKYTPQYKWLEAEFP-KVNRSETPWLIVLMHAPWYNSYNYHYMEGETMRVMYEPWFVKYKVDVVFAGHVHAYERTHRISNVAYNVVNGLCTPISDQSAPVYITIGDGGNQEGLATNMSQ---------------------------------PQPRYSAFREASFGHAILDIKNRTHAYYTWHRNQDG----SSVAADSMWFTNR------YW--------EPTDDSADDFQ-------

>SbPAP30a

----MMGDDLLLRRRGGVLFLVLSAVLVLLGGGA---------DAGVTSSYRRKLE--ATVEMPLDADVFRVP-PGYNAPQQVHITLGDQEGTAMTVSWVTA-SELG------------NSTVKFGE-KPDPEKM---ERRAEGTHTRY--------DY-FNYTSGFIHHCTLKHLKHSTKYYYA--MG-FGHT--VRTFSFTTPPKPGPDV-PFKFGLIGDLGQTFDSNITLSHYDDN--GGDAVLYVGDLSYADNHPL-----------------------------HD-NNRWDTWGRFMERNAAYQPWIWTAGNHELDFAPELGETTPFKPYTHRYPTP--YLSSGSTEPFWYSVKLASAHVVVLSSYSAYGKYTPQWTWLQAELQ-RVDRTTTPWLVVLMHSPWYNSNNYHYMEGETMRVQFEKWLVDARADVVLAGHVHSYERSHRVSNVAYDIVNGEATPVRDAGAPVYVTIGDGGNIEGVADNFTR---------------------------------PQPSYSAFREASFGHATLEIKNRTHAYYSWHRNHDG----AKVVADGVWLTNRAQSYAVFF--------LDRTKTSSMAIDHQP---

>SbPAP30c

----MHTVAIQRRRHGAVTLLLVAAAVVLLSAAAQTAAAHRPRGCGVTSKYVRKHQ--ASEDMPLDADVFAVP-PGRNAPQQVHIGLADQTGTSMFVSWVTV-EAEG------------NSTVLYGL---AADKL---DLAAEGTITRY--------TY-YNYTSGYIHHATLTNLQHGTRYHYAVGVG-VGDT--VRAFWFTTPPAPGPDV-PLRLGLIGDLGQTADSNSTLAHYESH--QGDAVLFVGDLSYADKHPL-----------------------------HD-NNRWDTWGRFAERSVAYQPWVWTTGNHEVDFAPELGELAPFKPFTHRYPTP--WKASQSSEPYWYSVKLGPAHIIVLSSYSAFGKYTPQYKWVEAELK-RVDREVTPWLFISTHVPWYNSNNFHYMEGEPMRAQLEKMAVDARVDAVFAGHVHAYERTHRYSNIKYNVTDGKCTPIADRRAPVYVVIGDGGNVEGLADELTW---------------------------------PQPAYSAFREYSFGHAVLDIKNRTHAYYAWYRNHDG----NKVTADTMWFTNR------YH--------MPNHDDSMTMITAKAAC-

>SbPAP30b

----MSNMLGARRRLGLVHVVLVLFAAALLVA-----------DAGVTSSYRRKLE--ATVEMPLDADVFGVP-LGYNAPQQVHITLGDIEGTSMIVSWVTA-NELG------------SSTVFYSEASPDPYMM---ELWAEGTHTRY--------NY-FNYTSGFIHHCNLTNLKYGTKYYYA--MG-FGHT--VRSFSFTTPPMPGPDV-PFKFGLIGDLGQTFDSNTTLSHYEAN--GGGAVLYVGDLSYADNRPL-----------------------------HD-NTRWDTWARFVERSAAHQPWVWTVGNHELDLAPELGEPVPFKPFAHRYPTPRRFAPAAAAPPFWYSVRIASAHVIVLASYSAYGKYTPQWKWLRGELA-RVDRAATPWLIVLVHSPWYSSNGYHYMEGETMRVQFERWLVAAKADLVVAGHVHAYERSHRVSNVAYDIVNGKCTPVRSRDAPVYVTVGDGGNIEGVADNFTQ---------------------------------PQPGYSAFREASFGHATLEIMNRTHAYYAWHRNQDG----AMVVADGVWFTNR------YW--------MPTDDDDTS---------

>SbPAP26

-------------MRGWGLLVLSLHVLSCLVSGV---------ASGRTSSYVRTEF--PSTDIPLDSEWFAIP-KGYNAPQQVHITQGDYDGKAVIVSWVTP-EEPG------------PSEVFYGK----EKQY---DQKSEGTTTNY--------TF-YDYKSGYIHHCLVDGLEYNTKYYYK--IG-SGDS--AREFWFETPPAIDPDA-SYTFGIIGDLGQTFNSLSTLQHYEKT--GGQTVLFVGDLSYADRYEH-----------------------------ND-GIRWDSWGRFVERSTAYQPWIWNTGNHEIEYRPDLGETSTFKPYLHRYMTP--YLASKSSSPMWYAVRRASAHIIVLSSYSPFVKYTPQWWWLKNEFK-RVDREKTPWLIVLMHSPMYNSNEAHYMEGESMRAAFEKWFVKYKVDLVFAGHVHAYERSYRISNVNYNITSGNRYPVPNKSAPVYITVGDGGNQEGLASRFYD---------------------------------PQPDYSAFREASYGHSLLQLKNRTHAVYQWNRNHDG----NPVPADTVVFHNQ------YWTSSTRRRRLKKNHFHLENLEDLISLF

>ZmPAP26

-------------MRGWGLLVLSLHVLSCLVSGV---------ASGRTSSYVRTEF--PSTDIPLESEWFAIP-KGYNAPQQVHITQGDYDGKAVIVSWVTP-EEPG------------PSEVFYGK----EKLY---DQKAEGTTTNY--------TF-YDYKSGYIHHCLVDGLEYNTKYYYK--IG-SGNS--AREFWFETPPAIDPDA-SYTFGIIGDLGQTFNSLSTLQHYEKT--GGQTVLFVGDLSYADRYEH-----------------------------ND-GIRWDSWGRFVEHSTAYQPWIWNTGNHEIEYRPDLGETSVFKPYLHRYMTP--YLASKSSSPMWYAVRRASAHIIVLSSYSPFVKYTPQWLWLKNEFK-RVDREKTPWLIVLMHSPMYNSNEAHYMEGESMRAAFEKWFVKYKVDLVFAGHVHAYERSYRISNVNYNITSGNRYPVPDKSAPVYITVGDGGNQEGLASRFYN---------------------------------PQPDYSAFREASYGHSVLQLKNRTHAIYQWNRNDDG----NPVPADTVMFHNQ------YWTSSTRRRRLKKNHLHLEDLEDLISLL

>ZmPAP10

MGRHGVDQIGAVAAFVWVTLLPLLLVCVVWPGA----------QAGHTSEYRRQLG--SAIDMPLDADVFRPP-PGYNAPEQVHITQGNHDGTAMIISWVTT-SEPG------------SSTVIYGT---SEDNL---NYTANGKHTQY--------TF-YNYTSGYIHHCTIKKLEFDTKYYYA--VG-IGQT--VRKFWFLTPPKSGPDV-PYTLGLIGDLGQSFDSNVTLTHYESNA-KAQAVLFVGDLSYADNYPY-----------------------------HD-NVRWDTWARFVERSVAYQPWIWTAGNHEIDFAPELGETKPFKPFSHRYPTP--YKASGSTAPYWYSIKRASAYIIVLASYSAYGKYTPQYKWLEAEFP-KVNRSETPWLVVLMHAPWYNSYNYHYMEGETMRVMYEPWFVKYKVDVVFAGHVHAYERTHRISNVAYNVVNGLCTPIPDQSAPVYITIGDGGNQEGLATNMSQ---------------------------------PQPSYSAFREASFGHAILDIKNRTHAYYTWHRNQDG----SAVAADSMWFTNR------YW--------EPTDDSADDFQ-------

>ZmPAP30a

------MMGPLPLRRRVGVLLFLALVLLGGGGGA---------DAGTTSSYRRKLE--ATVEMPLDADVFRVP-PGYNAPQQVHITLGDQEGTAMIVSWVTA-SEPG------------NSTVAYGE---DPARM---ERRADGAHTRY--------DY-FNYTSGFIHHCTLRNLKHATKYYYA--MG-FGHT--VRTFWFTTPPKPGPDV-PFKFGLIGDLGQTFDSNITLSHYESN--GGDAVLYVGDLSYADNHPL-----------------------------HD-NNRWDTWARFVERSVAYQPWVWTAGNHELDFAPELGETTPFKPFAHRYPTP--YRAAGSTEPFWYSVKVASAHVVVLASYSAYGKYTPQWAWLQAELA-RVDRKTTPWLVVLTHSPWYNSNNYHYMEGETMRVQFERWLVDAKVDLVLAGHVHSYERSHRVSNVAYDIVNGKSTPVRSADAPVYVTIGDGGNIEGIADNFTR---------------------------------PQPGYSAFREASFGHATLDIKNRTHAYYSWHRNHDG----AKVVADGVWFTNR------YW--------MPTDDDTN----------

>ZmPAP30b

--MSNVLRARRRLDLLQVVLFFVAVLLVVVA------------DAGVTSQYRRKLE--ATVEMPLDADVFRVP-PGYNAPQQVHITLGDQEGTAMIVSWVTA-NELG------------SSTVMYSEASPDPEKM---ELRAEGTHTRY--------DY-FNYTSGFIHHCTLTNLKHSTKYYYA--MG-FGHT--VRSFCFTTPPMPGPDV-PFKFGLIGDLGQTFDSNTTLSHYEAN--GGDAVLYVGDLSYADNHPL-----------------------------HD-NTRWDTWARFVERSAAHQPWVWTAGNHELDLAPELGEHVPFKPFAHRYPTP-----------FWYSVRVASAHVVVLASYSAYGKYTAQWEWLRAELA-RVDRAATPWLIVLVHSPWYSSNGYHYMEGETMRVQFERWIVAAKADLVVAGHVHAYERSHRVSNVAYDIINARCTPVRTRDAPVYVTVGDGGNIEGIADNFTQ---------------------------------PQPSYSAFREASFGHATLEIRNRTHAYYAWHRNQDG----AKVVADGVWLTNR------YW--------MPTDDDIN----------

>OsPAP10a

--------MVDRIGAAWWCACAVGMLVVGAC------------LAGETSEYRRQLG--SAVDMPLDADVFRAP-PGRNAPQQVHITQGNHDGTAMIISWVTT-IEPG------------SSTVLYGT---SEDNL---NFSADGKHTQY--------TF-YNYTSGYIHHCTIKKLEFDTKYYYA--VG-IGQT--VRKFWFRTPPKSGPDV-PYTFGLIGDLGQSYDSNITLAHYESNS-KAQAVLFVGDLCYADNYPY-----------------------------HD-NVRWDTWARFVERNVAYQPWIWTAGNHEIDFAPELGETKPFKPYSYRYPTP--YKASGSTAPFWYSVKRASAYIIVLASYSSYGKYTPQYKWLEAEFP-KVNRSETPWLIVLLHAPWYNSYNYHYMEGESMRVMYEPWFVKYKVDLVFAGHVHAYERTHRISNVAYNIVNGQCTPVHDQSAPVYITIGDGGNQEGLATNMTA---------------------------------PQPGYSAFRESSFGHAILDIKNRTHAYYTWHRNQDG----NAVAADSMWFTNR------YW--------QPTDESLDDSQ-------

>OsPAP10b

--------MVMEAACVLAVVVVVMAFLSPAA------------RGGVTSTYRRSLQ--ALPDMPIDADVFRPP-PGFNAPEQVHITLGDQTGRAMTVSWVTP-KLPD------------SNVVRYGL---RADNL---THTANGTFRRY--------SFGRKYRSGFIHHATLTGLDYGTKYHYA--VG-SGDTASARSFSFTTPPKPGPDV-PYKFGLIGDLGQTFHSNDTLSHYEAC--GGDAVLFIGDLSYADNHPG-----------------------------HD-NNRWDTWARFVERSVAYQPWIWTTGNHELDFAPELGETTPFKPFTNRYPTP--FGASGSTRPLWYSVRMASAHVIVLASYAAYGKYTPQWRWLEGELR-RVDRAVTPWLIVCVHSPWYSSNGYHYMEGESMRVEFERWLVDAKADVVLAGHVHSYERTRRVSNVAYDIANGMATPVFNRSAPVYINIGDGGNIEGLADDFRW---------------------------------PQPDYSVFREASFGHATLQIVNRTHAFYEWHRNSDG----VKVVADHAWFTNR------YW--------FPTDTN------------

>OsPAP10c

---------MGMLRWGAHLLLLLLAAATWTCAGA---------GAGVTSEYRRKLE--ATVDMPLDADVFRVP-PGYNAPQQ--------TGTAMTVSWVTA-NELG------------SNTVRYGS---SPEKL---DRAAEGSHTRY--------DY-FNYTSGFIHHCTLTGLTHATKYYYA--MG-FDHT--VRTFSFTTPPKPAPDA-PFKFGLIGDLGQTFDSNSTLAHYEAN--GGDAVLFVGDLSYADNYPL-----------------------------HD-NNRWDTWARFVERSVAYQPWIWTAGNHELDYAPELGETVPFKPFTHRYPTP--YRAAGSTEPFWYSVKIASAHVIVLASYSAYGKYTPQWTWLQEELATRVDRKLTPWLIVLMHSPWYNSNNYHYMEGETMRVQFERWLVDAKVDVVLAGHVHSYERSRRFANIDYNIVNGKATPAANVDAPVYITIGDGGNIEGIANNFTV---------------------------------PQPAYSAFREASFGHATLEIKNRTHAHYAWHRNHDG----AKAVADAVWLTNR------YW--------MPTNDDV-----------

>OsPAP10d

-------------------------------------------------------------------------------------------------------------------------------------------------------------------------------------------MG-FGFT--VRSFWFTTPPRPGPDV-AFRLGLIGDIGQTFDSNATLTHYEAS--GGDAVLFMGDLSYADKYPL-----------------------------HD-NNRWDTWGRFSERSVAYQPWIWVAGNHEIDYAPELGETKPFKPFTHRYPTP--HLASASPEPYWYSVKLASVHIIVLSSYSAFAKYTPQWKWLEAELG-RVNRSETPWLIMASHSPWYNSNNFHYMEGESMRAQLEKMAVDARVDLVFAGHVHAYERSFRVSNIRYNITDGLCTPVRDRRAPVYVTIGDGGNIEGLADEMTW---------------------------------PQPPYSAFREDSFGHAVLDIKNRTHAYYAWYRNDDG----AKVAADAVWFTNR------FH--------MPNHDDSTPTPTKRHYYG

>OsPAP26

--------------MGWRFALLLLHVLLCLVNGV---------SCGRTSSYVRTEY--PSTDIPLESEWFAVP-NGYNAPQQVHITQGDYNGKAVIVSWVTV-AEPG------------TSEVLYGK---NEHQY---DQRVEGTVTNY--------TF-YDYKSGYIHHCLVDGLEYNTKYYYK--IG-SGDS--AREFWFETPPAIDPDA-SYTFGIIGDLGQTFNSLSTLQHYEKS--EGQTVLFVGDLSYADRYQH-----------------------------ND-GVRWDSWGRLVERSTAYQPWIWSAGNHEIEYRPDLGETSTFKPYLHRCHTP--YLASKSSSPMWYAVRRASAHIIVLSSYSPFVKYTPQWTWLKYELK-HVDREKTPWLIVLMHSPMYNSNEAHYMEGESMRAAFEKWFVKYKVDLVFAGHVHAYERSYRISNINYNITSGNRYPVPDKSAPVYITVGDGGNQEGLASRFSD---------------------------------PQPDYSAFREASYGHSILQLKNRTHAIYQWNRNDDG----KHVPADNVVFHNQ------YWASNTRRRRLKKKHFHLDQIEDLISVF

>BrPAP10a

--------MKSDFSSVLLVLGLVLNSLVLFC------------NGGITSKFVRKVE--KSIDMPLHSDVFRVP-PGHNAPQQVHITQGDVEGKAVIVSWVTQ-EAPG------------SNTVLYWK---EHSSK---KHKAHGKTNTY--------KF-YNYTSGYIHHCTIRNLEYDSKYYYV--VG-VGQT--ERKFWFFTPPKVGPDV-PYTFGLIGDLGQTFDSNITLTHYEKSPKKGQAVLFVGDLSYADNHPN-----------------------------HD-NNRWDSWGRFAERSTAYQPWIWTTGNHELDFAPEIGENKPFKPFTHRYRTP--YRASGSTEPFWYSIKRGPAYIIVLSSYSAYGKYTPQYTWLEEEFP-KVNRTETPWLIVLNHSPWYNSYDYHYMEGETMRVMYEPWFVKNKVDVVFSGHVHAYERSERISNIAYTVVNGICSPVKDQSAPVYITIGDGGNIEGLATKMTE---------------------------------PQPKYSAYREASFGHAIFSIKNRTHAHYAWHRNQDG----YAVEADTMWFYNR------FW--------HPVNDSPSSDS-------

>BrPAP10b

----MGLRLEGPNLFLLLILGMVLNSLVLFC------------HGGRTSSYVRRLE--ATVDMPLDSDVFRVP-PGYNAPQQVHITQGDVEGKAVIVSWVTQ-EAPG------------SDTVLYWK---ENSSK---KLKAYGKSKTY--------KF-YNYTSGHIHHCTIRNLEYDTKYYYV--VG-VGQT--EREFYFFTPPEVGPDV-PYTFGLIGDLGQSFDSNITLTHYENNPTKGQAVLFVGDFSYADQYPN-----------------------------HD-NNRWDTWGRFAERSTAYQPWIWTVGNHELDFAPELGETKPFKPFLNRYRTP--YRSSGSTEPFWYSIKRGPAYIIVLASYSAYGKYTPQFKWLEKEFP-KVNRTETPWLIVLMHSPWYNSYDYHYMEGETMRVMYEPWFVKYKVDVVFAGHVHAYERSERVSNIAYNVVNGICTPVKDKSAPVYITIGDGGNLEGLATRMTE---------------------------------PQPKYSAFREASFGHAVLSIKNRTHAYYGWHRNQDG----YAVKGDTMWFYNR------FW--------HPIDDSPSE---------

>BrPAP10c

---------MKMGRFEGFFLSFVLNSFLLFC------------HGGTTSRFVRRLE--ATADMPLDSDVFRVP-PGCNAPQQVHITQGDLEGKAVIVSWVTQ-KAKG------------SNMVLYWK---EHSSK---MLKAHGKSKTY--------KF-YNYTSGHIHHCTIRNLEYDTKYYYM--VG-VGQT--ERKFWFLTPPKPGPDV-PYTFGLIGDLGQSFDSNITLTHYENNPLKGQTILFVGDFSYADTYPN-----------------------------HD-NNRWDTWGRFVERSTAYQPWIWTVGNHELDFAPQIGETKPFKPFKHRYHTP--HRSSGSTEPFWYSIKRGPAYIIVLASYSAYGKYTPQYMWLEQEFP-KVNRTETPWLIVLMHSPWYNSYDYHYMEGETMRVMYEPWFVKNKVDVVFAGHVHGYERSERISNIAYNVVNGICSPVKDLSAPVYITIGDGGNLEGLATIMTE---------------------------------PQPKYSAFREASFGHAIFSIKNRTHAYYGWHRNQDG----CAVDGDTMWFFNR------IW--------HPIDDSPDDDS-------

>BrPAP12

--MPLDSNVFIPDDRPLHSDVFL------------------------PDQKNKDSDVFVPDDMPLDSDVFKVP-PGPNTPQQVHITQ-----------------------------------VRYWS---ENGKL---KKLAEATINTY--------RF-FNYTSGYIHHCLIDDLEI---------------------FNLFL-------I-QYP----GDLGQTYDSNRTLSHYEMNPGKGQAVLFLGDLSYADLYKF-----------------------------HD-NNRWDTWGRFVERSAAYQPWIWTAGNHEIDFVPDIGETEPFKPFTNRYHTP--YKASGSISPLWYSIKRASAYIIVMSCYSSYGVYTPQYKWLRKEFQ-RVNRTETPWLIVLVHCPFYHSYVHHYMEGETMRVLYEQWFVKSKVDVVFAGHVHAYERSYLLC-------------------------------------------------------------------------PQ--------------------------------------------VIINY------------------------------------

>BrPAP26

------MIRGMEIHRLLVVVVAVLLSLVLNG------------EGGITSSYVRSEW--PAVDIPLDHKVFKVP-KGYNAPQQVHITQGDYDGKAVIISWVTP-DEPG------------SSTVHYGP---MQGEY---DFVAKGSYSNY--------TF-YKYKSGYTHHCLLSGLEYNTKYYYK--IE-SGES--SREFWFVTPPHVHPDA-SYKFGIIGDLGQTFNSLSTLEHYMRS--GAQAVLFLGDLSYADRYQY-----------------------------NDVGVRWDTWGRFVEPSTAYQPWLWSAGNHEVDYMPYMGEVTPFKNYLERYTTP--YLASKSSNPLWYAVRRASAHIIVLSSYSPFVKYTPQWRWLDAEFK-RVDREKTPWLIVLMHSPIYNSNEAHFKEGESMRAAFEEWFVEYRVDVVFAGHVHAYERSYRVSNVRYNVSSGDRFPVPDTSAPVYITVGDGGNQEGLAGRFME---------------------------------PQPDYSAFREASYGHSTLDIKNRTHAVYHWNRNDDG----KKVATDEFVLYNQ------YWGKNIRRRKLKKHYIKSVVVDWIAS--
