# Supplementary material for: The maize (Zea mays ssp. mays var. B73) genome encodes 33 members of the purple acid phosphatase family
Source: Front Plant Sci. 2015 May 19;6:341. doi: 10.3389/fpls.2015.00341 (PMC4436580; doi:10.3389/fpls.2015.00341)
Supplement: Supplementary file 2 [file Table2.PDF]

The maize (Zea mays ssp. mays var. B73) genome encodes 33 members of the purple acid phosphatase gene family. Eliécer González Muñoz, Aida-Odette Avendaño-Vázquez, Ricardo Aarón Chávez Montes, Stefan de Folter, Liliana Andrés-Hernández, Cei Abreu-Goodger and Ruairidh James Hay Sawers. Laboratorio Nacional de Genómica para la Biodiversidad (LANGEBIO), Centro de Investigación y de Estudios Avanzados del Instituto Politécnico Nacional (CINVESTAV-IPN), Irapuato C.P. 36821, Guanajuato, México. rsawers@langebio.cinvestav.mx

Supplementary Table S2. Table of subcellular localization, PHR1 binding sites and conserved residues for all identified putative maize PAP proteins.

The splice variant column indicates the name of the annotated protein(s) for the corresponding gene identifier.

When more than one variant exists, protein identifiers corresponding to primary transcripts are indicated in bold.

Predicted subcellular localization: S, secreted; M, mitochondria; C, chloroplast; O: other.

The length column indicates the length of the protein in aminoacid residues.

PAP conserved residues are those identified by Li *et al.*, 2002.

gene alias	gene id	subcellular localization	PHR1 binding sites		splice variant	length (aa)	PAP conserved residues				
			number	position (bp)			G**D**XG	G**D**XX**Y**	G**N**H(D/E)	VXX**H**	G**H**X**H**
ZmPAP7e	AC202435.3	O	1	-446	AC202435.3_FGP003	508		GDNFY	GNHD	VVGH	GHDH
ZmPAP2f	AC207043.3	O	0		AC207043.3_FGP004	320			GNHE	FIGH	GHVH
ZmPAP26	AC211394.4	S	1	-2352	AC211394.4_FGP004	476	GDLG	GDLSY	GNHE	VLMH	GHVH
ZmPAP2e	GRMZM2G007754	C	1	-824	GRMZM2G007754_P01	508			GNHE	FIGH	GHVH
ZmPAP23	GRMZM2G014193	M	1	-199	**GRMZM2G014193_P01**	567	GDLG	GDMTY	GNHE	AAWH	GHVH
					GRMZM2G014193_P02	565	GDLG	GDMTY	GNHE	AAWH	GHVH
					GRMZM2G014193_P03	566	GDLG	GDMTY	GNHE	AAWH	GHVH
ZmPAP30a	GRMZM2G073860	S	1	-256	GRMZM2G073860_P01	466	GDLG	GDLSY	GNHE	VLTH	GHVH
ZmPAP30b	GRMZM2G077466	S	0		GRMZM2G077466_P01	461	GDLG	GDLSY	GNHE	VLVH	GHVH
ZmPAP10	GRMZM2G093101	S	2	-232, -2463	**GRMZM2G093101_P01**	475	GDLG	GDLSY	GNHE	VLMH	GHVH
					GRMZM2G093101_P02	268	GDLG	GDLSY	GNHE		
ZmPAP28a	GRMZM2G096363	S			GRMZM2G096363_P01	396		GDNIf	GNHD	AFFH	GHDH
ZmPAP28b	GRMZM2G104676	S			GRMZM2G104676_P01	298		GDHFd	GNHD	ILTH	
ZmPAP2a	GRMZM2G106600	S	2	-138, -2268	GRMZM2G106600_P01	654	GDMG	GDISY	GNHE	FQGH	GHVH
ZmPAP7b	GRMZM2G109071	O	0		GRMZM2G109071_P01	253		GDNIY	GNHD	VVGH	
					**GRMZM2G109071_P02**	272		GDNIY	GNHD	VVGH	GHDH
					GRMZM2G109071_P03	235		GDNIY	GNHD	VVGH	
ZmPAP14	GRMZM2G109405	M			**GRMZM2G109405_P01**	397		GDNIY	GNHD	VFFH	GHDH
					GRMZM2G109405_P02	393		GDNIY	GNHD	VFFH	GHDH
					GRMZM2G109405_P03	393		GDNIY	GNHD	VFFH	GHDH
ZmPAP21b	GRMZM2G111425	O	0		GRMZM2G111425_P01	409	GDLG	GDLSY	GNHE	VLMH	cHTH
ZmPAP13	GRMZM2G134054	S	1	-412	**GRMZM2G134054_P01**	545	GDLG	GDVCY	GNHE	AGWH	GHVH
					GRMZM2G134054_P02	520	GDLG	GDVCY	GNHE	AGWH	GHVH
ZmPAP24b	GRMZM2G136453	S	0		**GRMZM2G136453_P01**	626	GDMG	GDMPY	GNHE	FATH	GHVH
					GRMZM2G136453_P02	90					
ZmPAP2d	GRMZM2G138698	C	2	-498, -791	GRMZM2G138698_P01	273					
					GRMZM2G138698_P02	634	GDMG	GDISY	GNHE	FIGH	GHVH
					**GRMZM2G138698_P03**	641	GDMG	GDISY	GNHE	FIGH	GHVH
ZmPAP2b	GRMZM2G138756	M	1	-618	GRMZM2G138756_P01	148					GHVH
					**GRMZM2G138756_P02**	651	GDMG	GDISY	GNHE	FIGH	GHVH

The maize (Zea mays ssp. mays var. B73) genome encodes 33 members of the purple acid phosphatase gene family. Eliécer González Muñoz, Aida-Odette Avendaño-Vázquez, Ricardo Aarón Chávez Montes, Stefan de Folter, Liliana Andrés-Hernández, Cei Abreu-Goodger and Ruairidh James Hay Sawers. Laboratorio Nacional de Genómica para la Biodiversidad (LANGEBIO), Centro de Investigación y de Estudios Avanzados del Instituto Politécnico Nacional (CINVESTAV-IPN), Irapuato C.P. 36821, Guanajuato, México. rsawers@langebio.cinvestav.mx

Supplementary Table S2. Table of subcellular localization, PHR1 binding sites and conserved residues for all identified putative maize PAP proteins (continued).

gene alias	gene id	subcellular localization	PHR1 binding sites		splice variant	length (aa)	PAP conserved residues				
			number	position (bp)			G**D**XG	G**D**XX**Y**	G**NH**(D/E)	VXX**H**	G**H**X**H**
ZmPAP7a	GRMZM2G141584	S	0		GRMZM2G141584_P01	348	GDWG	GDNIY	GNHD	VVGH	GHDH
ZmPAP7d	GRMZM2G152447	S	4	-436, -666, -708, -1811	**GRMZM2G152447_P01**	334	GDWG	GDNFY	GNHD	VVGH	GHDH
					GRMZM2G152447_P02	226			GNHD	VVGH	GHDH
ZmPAP7c	GRMZM2G152477	S	0		**GRMZM2G152477_P01**	349	GDWG	GDNFY	GNHD	AVGH	GHDH
					GRMZM2G152477_P02	173	GDWG	GDNFY	GNHD		
					GRMZM2G152477_P03	277	GrVG	GDNFY	GNHD	AVGH	GHDH
ZmPAP24a	GRMZM2G157027	S	0		GRMZM2G157027_P01	611	GDMG	GDITY	GNHE	FIAH	GHVH
ZmPAP18	GRMZM2G174549	S	0		GRMZM2G174549_P01	460	GDLG	GDLSY	GNHE	VLLH	GHVH
ZmPAP1c	GRMZM2G315848	M	1	-191	**GRMZM2G315848_P01**	617	GDMG	GDICY	GNHE	FLAH	GHVH
					GRMZM2G315848_P02	574	GDMG	GDICY	GNHE	FLAH	GHVH
					GRMZM2G315848_P03	93					
ZmPAP2c	GRMZM2G326625	O	2	-2038, -2140	GRMZM2G326625_P01	363	GDMG	GDISY	GNHE	FIGH	GHVH
ZmPAP7f	GRMZM2G351232	O	0		**GRMZM2G351232_P01**	543		GDNFY		VVGH	GHDH
					GRMZM2G351232_P02	138				VVGH	GHDH
ZmPAP16	GRMZM2G366607	M			GRMZM2G366607_P01	391		GDLVt	GNHD	IFWH	GHNH
ZmPAP1a	GRMZM2G386998	M	2	-391, -451	GRMZM2G386998_P01	669	GDMG	GDITY	GNHE	FLAH	GHVH
ZmPAP7g	GRMZM2G404769	M	1	-1368	**GRMZM2G404769_P01**	562		GDNFY		VVGH	GHDH
					GRMZM2G404769_P02	453		GDNFY	GNHD		
ZmPAP21c	GRMZM2G434170	S	4	-326, -1676, -1773, -1819	GRMZM2G434170_P01	447	GDLG	GDLSY	GNHE	ALVH	GHVH
ZmPAP21a	GRMZM5G831009	S	0		GRMZM5G831009_P01	470				VLLH	GHVH
					**GRMZM5G831009_P02**	452	GDLG	GDLSY	GNHE	VLLH	GHVH
ZmPAP1b	GRMZM5G868679	M	1	-2147	GRMZM5G868679_P01	619	GDMG	GDICY	GNHE	FLAH	GHVH
ZmPAP21d	GRMZM5G881649	S	2	-1462, -2357	GRMZM5G881649_P01	369	GDLG	GDLSY	GNHE	ALVH	

The maize (Zea mays ssp. mays var. B73) genome encodes 33 members of the purple acid phosphatase gene family. Eliécer González Muñoz, Aida-Odette Avendaño-Vázquez, Ricardo Aarón Chávez Montes, Stefan de Folter, Liliana Andrés-Hernández, Cei Abreu-Goodger and Ruairidh James Hay Sawers. Laboratorio Nacional de Genómica para la Biodiversidad (LANGEBIO), Centro de Investigación y de Estudios Avanzados del Instituto Politécnico Nacional (CINVESTAV-IPN), Irapuato C.P. 36821, Guanajuato, México. rsawers@langebio.cinvestav.mx

Supplementary Table S2. Table of subcellular localization, PHR1 binding sites and conserved residues for all identified putative maize PAP proteins (continued). Identified sequences not considered as PAPs.

gene id	splice variant	length (aa)	PAP conserved residues				
			GDXG	GDXXY	GNH(D/E)	VXXH	GHXH
AC209374.4	AC209374.4_FGP002	225					
	AC209374.4_FGP007	245			GNHE	FlGd	GHVH
GRMZM2G019019	GRMZM2G019019_P01	139				iFWH	
GRMZM2G046436	GRMZM2G046436_P01	310					
GRMZM2G076062	GRMZM2G076062_P01	690		GsNGY	GNes		
	GRMZM2G076062_P02	691		GsNGY	GNes		
GRMZM2G076989	GRMZM2G076989_P01	59					
	GRMZM2G076989_P02	47					GHIH
	GRMZM2G076989_P03	171					GHIH
GRMZM2G143984	GRMZM2G143984_P01	271					GHVH
GRMZM2G150236	GRMZM2G150236_P01	156			GNHD		
GRMZM2G306712	GRMZM2G306712_P01	92				FLVy	
GRMZM2G342815	GRMZM2G342815_P01	299					
GRMZM2G373887	GRMZM2G373887_P01	676					
GRMZM2G375011	GRMZM2G375011_P01	188			vNHE	FLQg	
GRMZM2G404941	GRMZM2G404941_P01	459					
GRMZM2G405770	GRMZM2G405770_P01	109					
