# Supplementary material for: The maize (Zea mays ssp. mays var. B73) genome encodes 33 members of the purple acid phosphatase family
Source: Front Plant Sci. 2015 May 19;6:341. doi: 10.3389/fpls.2015.00341 (PMC4436580; doi:10.3389/fpls.2015.00341)
Supplement: Supplementary file 3 [file Table3.PDF]

The maize (Zea mays ssp. mays var. B73) genome encodes 33 members of the purple acid phosphatase gene family. Eliécer González Muñoz, Aida-Odette Avendaño-Vázquez, Ricardo Aarón Chávez Montes, Stefan de Folter, Liliana Andrés-Hernández, Cei Abreu-Goodger and Ruairidh James Hay Sawers. Laboratorio Nacional de Genómica para la Biodiversidad (LANGEBIO), Centro de Investigación y de Estudios Avanzados del Instituto Politécnico Nacional (CINVESTAV-IPN), Irapuato C.P. 36821, Guanajuato, México. rsawers@langebio.cinvestav.mx

Supplementary Table S3. Bowtie2 alignment statistics for leaf and root samples under Pi sufficient and Pi deficient conditions.

The transcripts identified column indicates the number of transcripts with at least one effective count after eXpress processing. The Phytozome 10 maize transcripts file, version 6a, contains 88,744 sequences.

Organ	Treatment	File	Total reads	Reads aligned 1 time	Reads aligned > 1 times	% alignment	transcripts identified	transcripts identified (% of total)
leaf	Pi 1000 uM	B73_L_1000_B1.fastq	14,015,881	4,168,378	8,533,782	90.63	45,244	50.98
		B73_L_1000_B2.fastq	15,511,771	4,406,765	9,594,573	90.26	50,252	56.63
	Pi 10 uM	B73_L_10_B1.fastq	15,425,125	4,422,639	9,447,271	89.92	49,702	56.01
		B73_L_10_B2.fastq	24,269,289	6,876,456	14,914,671	89.79	51,120	57.60
root	Pi 1000 uM	B73_R_1000_B1.fastq	14,321,714	1,162,401	2,606,145	26.31	45,299	51.04
		B73_R_1000_B2.fastq	14,524,498	4,048,632	8,560,554	86.81	45,556	51.33
	Pi 10 uM	B73_R_10_B1.fastq	14,870,626	4,107,513	8,327,248	83.62	46,347	52.23
		B73_R_10_B2.fastq	15,876,137	4,326,735	9,331,339	86.03	49,272	55.52
