# Supplementary material for: The maize (Zea mays ssp. mays var. B73) genome encodes 33 members of the purple acid phosphatase family
Source: Front Plant Sci. 2015 May 19;6:341. doi: 10.3389/fpls.2015.00341 (PMC4436580; doi:10.3389/fpls.2015.00341)
Supplement: Supplementary file 4 [file Table4.PDF]

The maize (Zea mays ssp. mays var. B73) genome encodes 33 members of the purple acid phosphatase gene family. Eliécer González Muñoz, Aida-Odette Avendaño-Vázquez, Ricardo Aarón Chávez Montes, Stefan de Folter, Liliana Andrés-Hernández, Cei Abreu-Goodger and Ruairidh James Hay Sawers. Laboratorio Nacional de Genómica para la Biodiversidad (LANGEBIO), Centro de Investigación y de Estudios Avanzados del Instituto Politécnico Nacional (CINVESTAV-IPN), Irapuato C.P. 36821, Guanajuato, México. rsawers@langebio.cinvestav.mx

Supplementary Table S4. Differential expression for all identified putative maize PAPs in response to low soil phosphorous.

When more than one transcript exists, primary transcripts are indicated in bold. R1/L1 samples correspond to sufficient (1000 µM) soil phosphorous and R2/L2 samples correspond to low (10 µM) soil phosphorous. CPM: counts per million library counts (integer values). edgeR columns are the result of an edgeR analysis on the effective counts data (see Materials and Methods). Bonferroni-corrected pvalues (Bf) were calculated using the PValues for all 72 putative ZmPAP transcripts. Differentially expressed transcripts (Bf <= 0.05) are highlighted green (leaf) or orange (root and leaf).

			root								leaf							
			CPM				edgeR				CPM				edgeR			
**gene alias**	**gene id**	**transcript**	**R1.1**	**R1.2**	**R2.1**	**R2.2**	**logFC**	**logCPM**	**PValue**	**Bf**	**L1.1**	**L1.2**	**L2.1**	**L2.2**	**logFC**	**logCPM**	**PValue**	**Bf**
ZmPAP7e	AC202435.3	AC202435.3_FGT003	0	0	0	0	1,00	1,00	1,00	1	0	0	0	0	1,00	1,00	1,00	1
ZmPAP2f	AC207043.3	AC207043.3_FGT004	0	0	0	0	1,00	1,00	1,00	1	0	0	0	0	1,00	1,00	1,00	1
ZmPAP26	AC211394.4	AC211394.4_FGT004	149	68	58	61	-0,73	6,35	0,07	1	63	72	80	87	0,23	6,35	0,57	1
ZmPAP2e	GRMZM2G007754	GRMZM2G007754_T01	0	0	0	0	1,00	1,00	1,00	1	0	0	0	0	1,00	1,00	1,00	1
ZmPAP23	GRMZM2G014193	**GRMZM2G014193_T01**	9	5	7	8	0,20	3,31	0,76	1	6	5	12	17	1,27	3,31	0,03	1
		GRMZM2G014193_T02	8	5	5	4	-0,38	3,18	0,54	1	7	5	13	13	0,85	3,18	0,16	1
		GRMZM2G014193_T03	2	7	7	9	0,75	2,77	0,33	1	3	5	5	10	0,78	2,77	0,31	1
ZmPAP30a	GRMZM2G073860	GRMZM2G073860_T01	20	17	36	35	1,04	4,21	0,02	1	4	5	17	21	1,90	4,21	4,40E-05	0,003
ZmPAP30b	GRMZM2G077466	GRMZM2G077466_T01	0	0	0	0	1,00	1,00	1,00	1	0	0	0	0	1,00	1,00	1,00	1
ZmPAP10	GRMZM2G093101	**GRMZM2G093101_T01**	46	37	72	88	1,04	6,42	0,004	0,277	17	16	168	179	3,27	6,42	1,96E-17	1,41E-15
		GRMZM2G093101_T02	0	0	0	0	5,13E-15	-2,25	1,00	1	0	0	0	0	5,12	-2,25	0,29	1
ZmPAP28a	GRMZM2G096363	GRMZM2G096363_T01	16	19	17	23	0,29	3,47	0,61	1	6	5	5	5	-0,18	3,47	0,76	1
ZmPAP28b	GRMZM2G104676	GRMZM2G104676_T01	13	10	6	12	-0,23	2,67	0,74	1	2	2	3	4	0,35	2,67	0,64	1
ZmPAP2a	GRMZM2G106600	GRMZM2G106600_T01	71	64	88	98	0,56	6,30	0,13	1	36	53	97	104	1,10	6,30	0,00	0,199
ZmPAP7b	GRMZM2G109071	GRMZM2G109071_T01	1	0	0	1	1,09	-0,91	0,57	1	0	0	0	0	-3,27	-0,91	0,22	1
		**GRMZM2G109071_T02**	0	0	0	0	2,69	-1,86	0,25	1	0	0	0	0	-2,57	-1,86	1,00	1
		GRMZM2G109071_T03	0	0	0	0	1,00	1,00	1,00	1	0	0	0	0	1,00	1,00	1,00	1
ZmPAP14	GRMZM2G109405	**GRMZM2G109405_T01**	122	90	59	57	-0,76	5,35	0,10	1	11	28	9	11	-1,05	5,35	0,03	1
		GRMZM2G109405_T02	0	0	0	0	2,45	-1,85	0,44	1	0	0	0	0	-0,23	-1,85	1,00	1
		GRMZM2G109405_T03	9	15	2	6	-1,38	2,27	0,14	1	0	4	1	2	-0,22	2,27	0,83	1
ZmPAP21b	GRMZM2G111425	GRMZM2G111425_T01	0	0	0	0	-0,65	-1,52	0,74	1	0	0	0	0	4,68	-1,52	0,16	1
ZmPAP13	GRMZM2G134054	**GRMZM2G134054_T01**	14	68	70	89	0,99	6,43	0,08	1	13	12	175	184	3,70	6,43	5,92E-09	4,26E-07
		GRMZM2G134054_T02	18	10	131	116	3,23	5,96	6,00E-13	4,32E-11	0	0	102	114	10,02	5,96	1,46E-47	1,05E-45
ZmPAP24b	GRMZM2G136453	**GRMZM2G136453_T01**	116	92	112	101	0,14	7,05	0,76	1	10	31	257	272	3,59	7,05	4,74E-12	3,41E-10
		GRMZM2G136453_T02	0	0	0	0	5,05	-1,96	0,23	1	0	0	0	0	-0,61	-1,96	1,00	1

The maize (Zea mays ssp. mays var. B73) genome encodes 33 members of the purple acid phosphatase gene family. Eliécer González Muñoz, Aida-Odette Avendaño-Vázquez, Ricardo Aarón Chávez Montes, Stefan de Folter, Liliana Andrés-Hernández, Cei Abreu-Goodger and Ruairidh James Hay Sawers. Laboratorio Nacional de Genómica para la Biodiversidad (LANGEBIO), Centro de Investigación y de Estudios Avanzados del Instituto Politécnico Nacional (CINVESTAV-IPN), Irapuato C.P. 36821, Guanajuato, México. rsawers@langebio.cinvestav.mx

Supplementary Table S4. Differential expression for all identified putative maize PAPs in response to low soil phosphorous (continued).

			root								leaf							
			CPM				edgeR				CPM				edgeR			
**gene alias**	**gene id**	**transcript**	**R1.1**	**R1.2**	**R2.1**	**R2.2**	**logFC**	**logCPM**	**PValue**	**Bf**	**L1.1**	**L1.2**	**L2.1**	**L2.2**	**logFC**	**logCPM**	**PValue**	**Bf**
ZmPAP2d	GRMZM2G138698	GRMZM2G138698_T01	0	0	0	0	1,00	1,00	1,00	1	0	0	0	0	1,00	1,00	1,00	1
		GRMZM2G138698_T02	0	0	1	3	2,46	0,19	0,06	1	0	0	0	1	0,69	0,19	0,63	1
		**GRMZM2G138698_T03**	0	0	0	0	1,00	1,00	1,00	1	0	0	0	0	1,00	1,00	1,00	1
ZmPAP2b	GRMZM2G138756	GRMZM2G138756_T01	0	0	11	15	10,46	2,92	3,23E-11	2,32E-09	0	0	14	14	6,42	2,92	3,84E-10	2,77E-08
		**GRMZM2G138756_T02**	0	3	2	8	1,44	2,96	0,12	1	0	3	17	16	3,08	2,96	1,79E-03	0,129
ZmPAP7a	GRMZM2G141584	GRMZM2G141584_T01	0	0	0	0	2,16	-2,90	1,00	1	0	0	0	0	-5,13E-15	-2,90	1,00	1
ZmPAP7d	GRMZM2G152447	**GRMZM2G152447_T01**	180	198	445	397	1,25	8,62	0,00	0,137	47	94	749	805	3,37	8,62	7,91E-15	5,70E-13
		GRMZM2G152447_T02	0	2	0	2	0,58	3,54	0,66	1	0	2	32	32	4,39	3,54	4,04E-03	0,291
ZmPAP7c	GRMZM2G152477	**GRMZM2G152477_T01**	129	143	185	171	0,49	6,81	0,29	1	9	25	138	147	2,97	6,81	1,45E-09	1,04E-07
		GRMZM2G152477_T02	0	0	0	0	0,76	-0,03	0,62	1	0	0	2	2	8,32	-0,03	1,40E-04	0,010
		GRMZM2G152477_T03	3	6	6	10	0,87	2,50	0,28	1	0	1	10	5	2,66	2,50	1,91E-03	0,138
ZmPAP24a	GRMZM2G157027	GRMZM2G157027_T01	23	18	23	24	0,27	3,57	0,66	1	4	1	6	7	1,24	3,57	0,05	1
ZmPAP18	GRMZM2G174549	GRMZM2G174549_T01	149	119	140	124	0,09	6,36	0,81	1	34	46	51	59	0,37	6,36	0,31	1
ZmPAP1c	GRMZM2G315848	**GRMZM2G315848_T01**	58	73	134	146	1,18	6,31	0,00	0,091	25	25	94	97	1,83	6,31	9,17E-07	6,60E-05
		GRMZM2G315848_T02	0	0	0	0	5,13E-15	-2,83	1,00	1	0	0	0	0	2,79	-2,83	1,00	1
		GRMZM2G315848_T03	0	0	0	0	5,13E-15	-2,67	1,00	1	0	0	0	0	3,81	-2,67	0,45	1
ZmPAP2c	GRMZM2G326625	GRMZM2G326625_T01	0	1	2	2	1,00	0,36	0,43	1	0	0	0	1	0,65	0,36	0,64	1
ZmPAP7f	GRMZM2G351232	**GRMZM2G351232_T01**	0	0	0	0	1,00	1,00	1,00	1	0	0	0	0	1,00	1,00	1,00	1
		GRMZM2G351232_T02	0	0	0	0	4,05	-1,14	0,33	1	0	0	1	0	3,61	-1,14	0,10	1
ZmPAP16	GRMZM2G366607	GRMZM2G366607_T01	8	10	37	26	1,85	4,83	4,37E-05	0,003	2	2	63	56	4,40	4,83	2,75E-18	1,98E-16
ZmPAP1a	GRMZM2G386998	GRMZM2G386998_T01	40	33	33	36	0,01	4,24	0,98	1	9	8	5	8	-0,52	4,24	0,26	1
ZmPAP7g	GRMZM2G404769	**GRMZM2G404769_T01**	0	0	0	0	-4,32	-2,74	0,49	1	0	0	0	0	-5,13E-15	-2,74	1,00	1
		GRMZM2G404769_T02	0	0	0	0	5,13E-15	-1,83	1,00	1	0	0	0	0	3,39	-1,83	0,16	1
ZmPAP21c	GRMZM2G434170	GRMZM2G434170_T01	4	5	6	7	0,59	1,77	0,50	1	0	0	2	3	5,27	1,77	2,18E-05	0,002
ZmPAP21a	GRMZM5G831009	GRMZM5G831009_T01	0	0	0	0	-0,96	-2,21	0,72	1	0	0	0	0	-5,13E-15	-2,21	1,00	1
		**GRMZM5G831009_T02**	4	4	2	2	-0,89	0,52	0,46	1	0	0	0	0	-5,13E-15	0,52	1,00	1
ZmPAP1b	GRMZM5G868679	GRMZM5G868679_T01	112	65	51	55	-0,61	5,06	0,14	1	5	6	14	16	1,33	5,06	0,00	0,149
ZmPAP21d	GRMZM5G881649	GRMZM5G881649_T01	0	3	8	8	2,01	2,09	0,03	1	0	0	5	6	7,31	2,09	4,81E-08	3,46E-06

The maize (Zea mays ssp. mays var. B73) genome encodes 33 members of the purple acid phosphatase gene family. Eliécer González Muñoz, Aida-Odette Avendaño-Vázquez, Ricardo Aarón Chávez Montes, Stefan de Folter, Liliana Andrés-Hernández, Cei Abreu-Goodger and Ruairidh James Hay Sawers. Laboratorio Nacional de Genómica para la Biodiversidad (LANGEBIO), Centro de Investigación y de Estudios Avanzados del Instituto Politécnico Nacional (CINVESTAV-IPN), Irapuato C.P. 36821, Guanajuato, México. rsawers@langebio.cinvestav.mx

Supplementary Table S4. Differential expression for all identified putative maize PAPs in response to low soil phosphorous (continued). Identified sequences not considered as PAPs.

		root								leaf							
		CPM				edgeR				CPM				edgeR			
**gene id**	**transcript**	**R1.1**	**R1.2**	**R2.1**	**R2.2**	**logFC**	**logCPM**	**PValue**	**Bf**	**L1.1**	**L1.2**	**L2.1**	**L2.2**	**logFC**	**logCPM**	**PValue**	**Bf**
AC209374.4	AC209374.4_FGT002	19	18	29	23	0,59	4,90	0,15	1	31	27	32	35	0,15	4,90	0,71	1
	**AC209374.4_FGT007**	0	0	0	1	5,81	-1,79	0,22	1	0	0	0	0	4,39	-1,79	0,41	1
GRMZM2G019019	GRMZM2G019019_T01	0	0	0	0	4,69	-2,49	0,38	1	0	0	0	0	-5,13E-15	-2,49	1,00	1
GRMZM2G046436	GRMZM2G046436_T01	0	0	0	0	4,04	-2,64	0,47	1	0	0	0	0	-5,13E-15	-2,64	1,00	1
GRMZM2G076062	GRMZM2G076062_T01	99	152	145	150	0,32	6,46	0,43	1	54	44	45	74	0,20	6,46	0,63	1
	**GRMZM2G076062_T02**	105	110	178	171	0,79	6,26	0,04	1	19	32	42	43	0,65	6,26	0,09	1
GRMZM2G076989	GRMZM2G076989_T01	15	15	8	7	-0,85	3,84	0,44	1	30	0	17	9	-0,26	3,84	0,81	1
	GRMZM2G076989_T02	0	0	0	0	1,00	1,00	1,00	1	0	0	0	0	1,00	1,00	1,00	1
	**GRMZM2G076989_T03**	1	1	5	1	0,87	3,27	0,30	1	11	30	4	4	-2,23	3,27	0,01	0,400
GRMZM2G143984	GRMZM2G143984_T01	0	0	0	0	3,74	-2,64	0,54	1	0	0	0	0	2,02	-2,64	1,00	1
GRMZM2G150236	GRMZM2G150236_T01	4	2	7	5	0,97	1,84	0,25	1	2	2	2	2	0,01	1,84	1,00	1
GRMZM2G306712	GRMZM2G306712_T01	0	0	0	0	1,00	1,00	1,00	1	0	0	0	0	1,00	1,00	1,00	1
GRMZM2G342815	GRMZM2G342815_T01	0	0	0	0	5,13E-15	-2,76	1,00	1	0	0	0	0	3,29	-2,76	0,70	1
GRMZM2G373887	GRMZM2G373887_T01	1	0	0	0	-1,55	-0,96	0,38	1	0	0	0	0	5,72	-0,96	0,05	1
GRMZM2G375011	GRMZM2G375011_T01	1	1	1	2	0,28	0,53	0,83	1	0	1	0	1	0,11	0,53	0,95	1
GRMZM2G404941	GRMZM2G404941_T01	1	0	0	0	-2,38	-0,22	0,35	1	1	0	0	1	0,91	-0,22	0,71	1
GRMZM2G405770	GRMZM2G405770_T01	0	0	0	0	1,00	1,00	1,00	1	0	0	0	0	1,00	1,00	1,00	1
